# Precise transcript targeting by CRISPR-Csm complexes

**DOI:** 10.1038/s41587-022-01649-9

**Published:** 2023-01-23

**Authors:** David Colognori, Marena Trinidad, Jennifer A. Doudna

**Affiliations:** 1 Department of Molecular and Cell Biology, University of California, Berkeley, CA USA; 2 Innovative Genomics Institute, University of California, Berkeley, CA USA; 3 Howard Hughes Medical Institute, University of California, Berkeley, CA USA; 4 Department of Chemistry, University of California, Berkeley, CA USA; 5 California Institute for Quantitative Biosciences (QB3), University of California, Berkeley, CA USA; 6 Molecular Biophysics and Integrated Bioimaging Division, Lawrence Berkeley National Laboratory, Berkeley, CA USA; 7 Gladstone Institutes, San Francisco, CA USA

**Keywords:** Expression systems, Functional genomics

## Abstract

Robust and precise transcript targeting in mammalian cells remains a difficult challenge using existing approaches due to inefficiency, imprecision and subcellular compartmentalization. Here we show that the clustered regularly interspaced short palindromic repeats (CRISPR)-Csm complex, a multiprotein effector from type III CRISPR immune systems in prokaryotes, provides surgical RNA ablation of both nuclear and cytoplasmic transcripts. As part of the most widely occurring CRISPR adaptive immune pathway, CRISPR-Csm uses a programmable RNA-guided mechanism to find and degrade target RNA molecules without inducing indiscriminate *trans*-cleavage of cellular RNAs, giving it an important advantage over the CRISPR-Cas13 family of enzymes. Using single-vector delivery of the *Streptococcus thermophilus* Csm complex, we observe high-efficiency RNA knockdown (90–99%) and minimal off-target effects in human cells, outperforming existing technologies including short hairpin RNA- and Cas13-mediated knockdown. We also find that catalytically inactivated Csm achieves specific and durable RNA binding, a property we harness for live-cell RNA imaging. These results establish the feasibility and efficacy of multiprotein CRISPR-Cas effector complexes as RNA-targeting tools in eukaryotes.

## Abstract (web summary)

The bacterial Csm complex efficiently knocks down eukaryotic nuclear and cytoplasmic RNAs.

## Main

The ability to alter RNA and protein levels in cells and organisms without making permanent changes to DNA has proven invaluable for both basic research and therapeutics. For the past two decades, targeted RNA knockdown (KD) in eukaryotes has been accomplished using RNA interference (RNAi), an approach whereby small interfering RNAs (siRNAs) direct endogenous Argonaute nucleases to cleave complementary target RNAs^1,2^. However, RNAi can cause unintended cleavage of targets carrying partial sequence complementarity, especially when this complementarity occurs within the nucleating ‘seed’ region of the siRNA^3–5^. Furthermore, siRNAs are inefficient at targeting nuclear RNAs because the RNAi machinery localizes primarily to the cytoplasm^6,7^. Finally, RNAi is incompatible with certain eukaryotic model systems, including budding yeast that lacks RNAi machinery^8,9^ and zebrafish embryos that suffer from nonspecific developmental defects^10,11^. Thus, there has been ongoing interest in developing new RNA KD tools with higher specificity and broader targeting capability.

**Fig. 1:**
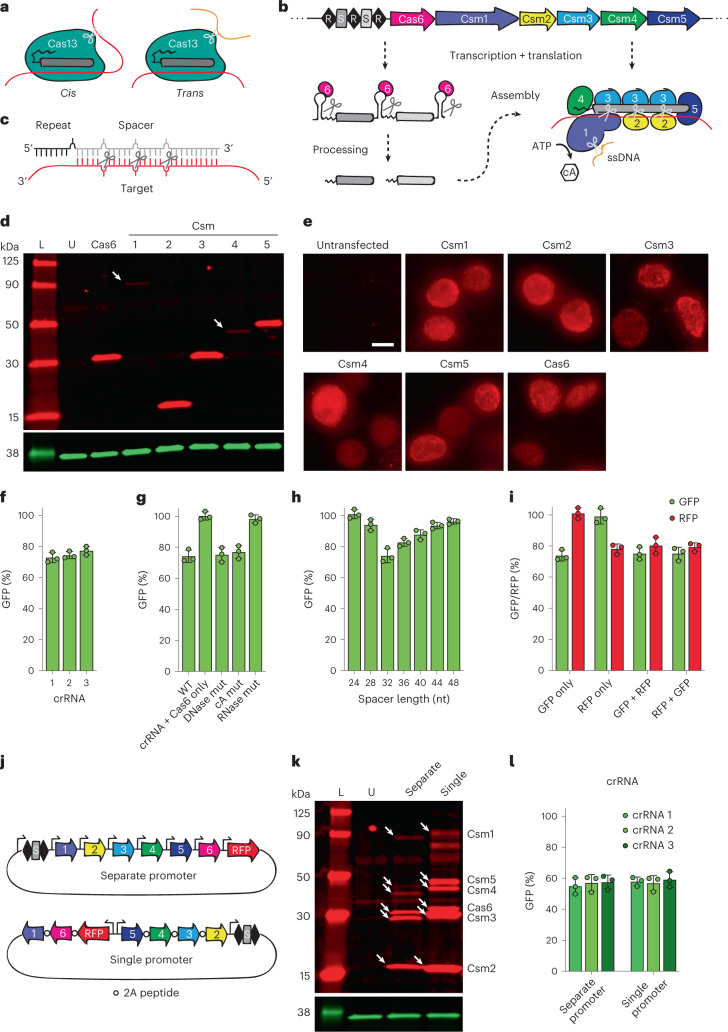
An all-in-one type III CRISPR-Cas system in human cells. **a**, Diagram showing *cis*- and *trans*-cleavage of Cas13. **b**, Diagram showing *S. thermophilus* type III-A CRISPR-Cas locus. crRNAs are transcribed from the CRISPR array, processed by Cas6 and assemble with Csm proteins. **c**, Close-up of crRNA:target binding, showing the 6-nt cleavage pattern. **d**, Western blot showing proper size and expression of Cas/Csm proteins (red) in HEK293T cells. Csm1 and Csm4 are less stable when expressed separately^72^. GAPDH (glyceraldehyde-3-phosphate dehydrogenase) shown as loading control (green). Arrows indicate faint bands. L, ladder; U, untransfected. One of two replicates with similar results is shown. **e**, Immunofluorescence showing expression and nuclear localization of Cas/Csm proteins in HEK293T cells. Scale bar, 10 μm. One of two replicates with similar results is shown. **f**, Relative GFP fluorescence (= MFI targeting crRNA/MFI nontargeting crRNA) of HEK293T-GFP cells transfected with plasmids expressing Cas6, Csm1-5 and the indicated GFP-targeting crRNA, measured by flow cytometry. Error bars indicate mean ± s.d. of three biological replicates. **g**, Same as **f**, but with the indicated Csm mutants (or crRNA + Cas6 only). GFP crRNA 1 was used to target GFP. Error bars indicate mean ± s.d. of three biological replicates. **h**, Same as **f**, but with GFP crRNA 1 adjusted to the indicated spacer length. Error bars indicate mean ± s.d. of three biological replicates. **i**, Relative GFP and RFP fluorescence of HEK293T-GFP/RFP cells transfected with plasmids expressing Cas6, Csm1-5 and the indicated crRNAs (individual or multiplexed), measured by flow cytometry. GFP crRNA 1 was used to target GFP. RFP-targeting crRNA is listed in Supplementary Table 1. Error bars indicate mean ± s.d. of three biological replicates. **j**, Diagram showing all-in-one delivery vector designs. **k**, Western blot showing proper size and expression of Cas/Csm proteins (red) in HEK293T cells. GAPDH is shown as loading control (green). Arrows indicate each subunit. One of two replicates with similar results is shown. **l**, Relative GFP fluorescence of HEK293T-GFP cells transfected with the indicated delivery vectors and expressing the indicated GFP-targeting crRNAs, measured by flow cytometry. Error bars indicate mean ± s.d. of three biological replicates. Source data

Clustered regularly interspaced short palindromic repeats (CRISPR)-CRISPR-associated proteins (Cas), which comprise adaptive defense systems against infectious agents in prokaryotes^12,13^, operate as programmable DNA or RNA nucleases^14–16^. Similar to RNAi, Cas nucleases use small RNAs, or CRISPR RNAs (crRNAs), to recognize nucleic acid targets via base-pairing complementarity. One such nuclease, Cas13, has gained attention as a new RNA-cleavage tool for use in eukaryotes^17–19^. However, unlike Argonaute proteins that cut only complementary RNAs in *cis*^20^, Cas13 also degrades nearby noncomplementary RNAs in *trans*^18,21^ (Fig. 1a). This is because the nuclease domains of Cas13 are located away from the crRNA:target binding pocket on an exposed surface of the protein^22–25^. Cas13’s *trans*-cleavage activity is readily detectable in vitro, where it has been exploited for viral RNA detection tools^21,26–29^. In bacteria, *trans*-cleavage leads to stalled cell growth or cell death (abortive infection)^30^, which is now believed to be Cas13’s primary mode of defensive action against viral infection. Only recently, however, has evidence mounted that Cas13 exhibits *trans*-cleavage activity in eukaryotic cells, causing cytotoxicity and/or cell death^31–35^. The convolution of *cis*- and *trans*-cutting effects has made it difficult to interpret results obtained using Cas13, and call into question its utility as a tool for specific RNA KD.

Despite their higher prevalence in nature^36^, multisubunit Cas effectors have been harnessed only rarely as tools in eukaryotes (with few exceptions^37–39^) compared to single-subunit effectors, due to their component complexity. Nonetheless, the well-studied biochemical and structural properties of type III RNA-targeting CRISPR-Csm complexes make them of particular interest for potential transcript targeting tools. The multiprotein Csm complex comprises five subunits (Csm1-5) in varying stoichiometries and relies on an additional protein, Cas6, for processing the precursor crRNA^40–47^ (Fig. 1b). The crRNA lies at the core of the complex, with Csm1 and Csm4 binding the 5′ end, Csm5 binding the 3′ end and multiple copies of Csm2 and Csm3 wrapping around the center. The complex contains a groove along its length into which target RNAs can enter and hybridize to the variable spacer region of the crRNA. Csm1 and Csm4 specifically recognize the 5′ region of the crRNA derived from the CRISPR repeat. Each Csm3 subunit has ribonuclease (RNase) activity, leading to multiple cleavage sites within the target RNA spaced six nucleotides (nt) apart (Fig. 1c). Csm1 functions as a nonspecific single-stranded DNase (ssDNase)^48,49^ and a cyclic oligoadenylate (cA) synthase^50,51^ (Fig. 1b). The ssDNase activity is thought to defend against actively transcribed (R-looped) or ssDNA foreign genomes^48,49^, while the latter acts as a second messenger that activates downstream effectors in *trans*, such as the RNase Csm6 (refs. ^50,51^). Notably, all three catalytic activities are performed by independent domains of the Csm complex and can be individually ablated.

Csm is an attractive RNA KD tool over current methods. A self-contained system found only in prokaryotes, it can be orthogonally introduced into eukaryotes without intersecting host RNA regulatory pathways. Furthermore, unlike RNAi, it can be localized to the nucleus and used to target nuclear noncoding RNAs and pre-mRNAs. Compared to Cas13, Csm cleaves only in *cis* within the crRNA:target complementary region and thus does not suffer from *trans*-cleavage activity^40^. Additionally, unlike Cas13, Csm-mediated RNA cleavage does not preferentially occur at a particular nt base (for example, U)^18,27^ nor is directly influenced by sequence flanking the target (for example, tag:antitag complementarity)^43,52^. In this work, we demonstrate the utility of the Csm system as a highly efficient, specific and versatile RNA KD tool in eukaryotes.

## Results

### An all-in-one type III CRISPR-Cas system in human cells

We chose the type III-A Csm complex from *Streptococcus* *thermophilus* for several reasons as follows: (1) it has been extensively characterized biochemically, structurally and in bacteria^40–47^, (2) it functions optimally at 37 °C, (3) it has been demonstrated to work in zebrafish embryos and human cell culture upon ribonucleoprotein (RNP) delivery^53,54^ and (4) it has fewer components than the analogous type III-B Cmr complex^55^. We began by verifying proper expression of each individual protein component (Csm1-5 and Cas6) in immortalized human embryonic kidney (HEK293T) cells. Proteins were human codon optimized, N-terminally FLAG-tagged for detection and expressed from a cytomegalovirus promoter. While RNAi operates in the cytoplasm where mRNAs mainly reside, we chose to localize Cas6 and each Csm component to the nucleus through the addition of an N-terminal SV40 nuclear localization signal so as to target nuclear RNAs and pre-mRNAs before export. Following transient transfection, Western blot (Fig. 1d) and immunofluorescence staining (Fig. 1e) verified proper size, expression and nuclear localization of each protein.

To test our system, we targeted enhanced green fluorescent protein (eGFP; henceforth ‘GFP’) mRNA in a GFP-expressing HEK293T cell line. Seven plasmids individually expressing Csm1-5, Cas6 and either a GFP-targeting or nontargeting crRNA from a U6 promoter were cotransfected into cells, and GFP fluorescence assayed by flow cytometry 48 h post transfection (Supplementary Fig. 1a). Note that this strategy does not allow any means to select cells into which all plasmids were successfully delivered and will thus under-report KD efficiency. GFP KD was calculated by dividing the mean fluorescence intensity (MFI) of cells transfected with the GFP-targeting crRNA by that of cells transfected with the nontargeting crRNA (Supplementary Fig. 1b). Approximately 25% KD was observed using any of three crRNAs targeting different regions of the GFP ORF (Fig. 1f). Notably, no KD was seen after transfecting the GFP-targeting crRNA and its processing factor (Cas6) alone (Fig. 1g), indicating that KD was not due to an antisense RNA effect. Furthermore, whereas ablating DNase (H15A, D15A) or cA synthase (D577A, D578A) activities in Csm1 did not noticeably affect GFP KD, ablating RNase activity (D33A) in Csm3 abolished it (Fig. 1g), indicating RNase activity is responsible for the observed KD.

Next, we examined crRNA parameters. Naturally occurring spacers for *Sth*Csm crRNAs range from ~30 to 45 nt in length, although in vitro, spacers as short as 27 nt are sufficient to trigger all three catalytic activities^42^. We varied the GFP-targeting spacer length from 24 nt to 48 nt in increments of four and assayed GFP KD. A length of 32 nt yielded the highest KD for the crRNA tested (Fig. 1h), with little to no KD seen for lengths ≤28 nt, and diminishing KD seen for lengths ≥36 nt. A more large-scale analysis must be performed to determine whether optimal spacer length differs from sequence to sequence. Next, we examined the potential to multiplex crRNAs against several targets. We encoded two crRNAs within a single array—one targeting GFP and the other targeting mCherry (henceforth ‘red fluorescent protein (RFP)’)—and examined KD of GFP and RFP in a HEK293T cell line expressing both (Fig. 1i). Approximately 25% KD was achieved for both GFP and RFP regardless of the order of crRNAs in the array (GFP–RFP or RFP–GFP), comparable to KD efficiency when targeting GFP or RFP alone. Together, these results demonstrate broad multiplexing capability for the Csm system.

With the Csm system up and running, we sought to simplify its delivery by consolidating all components into a single vector. For this, we pursued the following two approaches concurrently: (1) expression of each protein from separate promoters or (2) expression of all proteins from a single bidirectional promoter separated by 2A peptides (Fig. 1j). We also included RFP in the plasmid backbone to allow identification of transfected cells and thus more accurate measurement of KD efficiency (Supplementary Fig. 1c). After reconfirming proper expression of all protein components by Western blot for both plasmids (Fig. 1k), we found both strategies (after optimizing the order of proteins in the single-promoter arrangement) led to ~50% GFP KD in transfected cells (Fig. 1l). In summary, the single-promoter design is well-equipped for promoter-swapping and thus use in specific cell types or other eukaryotic systems, while the modular design of the separate-promoter vector allows for easy swapping or modification of individual Csm components. All further experiments were performed using the separate-promoter vector.

### Robust KD of endogenous nuclear and cytoplasmic RNAs

**Fig. 2:**
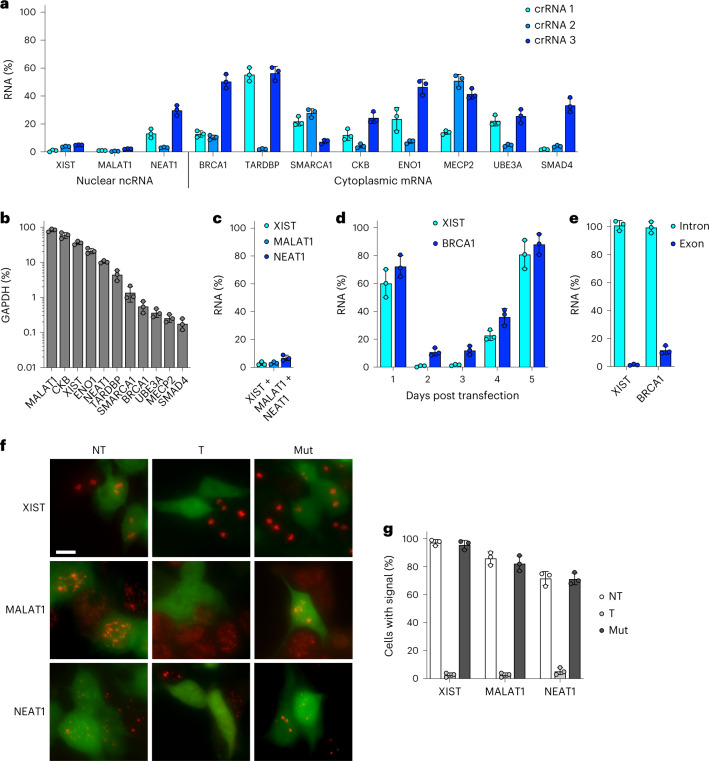
Robust KD of endogenous nuclear and cytoplasmic RNAs. **a**, Relative RNA abundance (normalized to nontargeting crRNA) of the indicated targets in HEK293T cells transfected with all-in-one plasmid expressing Cas/Csm proteins and the indicated crRNAs, measured by RT-qPCR. Error bars indicate mean ± s.d. of three biological replicates. **b**, Relative RNA abundance (normalized to GAPDH) of the indicated targets in untransfected HEK293T cells, measured by RT-qPCR. Error bars indicate mean ± s.d. of three biological replicates. **c**, Relative RNA abundance (normalized to nontargeting crRNA) of the indicated targets in HEK293T cells transfected with all-in-one plasmid expressing Cas/Csm proteins and the indicated crRNAs (multiplexed), measured by RT-qPCR. XIST crRNA 1, MALAT1 crRNA 1 and NEAT1 crRNA 2 were used to target XIST, MALAT1 and NEAT1, respectively. Error bars indicate mean ± s.d. of three biological replicates. **d**, Relative RNA abundance (normalized to nontargeting crRNA) of XIST and BRCA1 in HEK293T cells at the indicated times post transfection with all-in-one plasmid, measured by RT-qPCR. XIST crRNA 1 and BRCA1 crRNA 2 were used to target XIST and BRCA1, respectively. Error bars indicate mean ± s.d. of three biological replicates. **e**, Relative RNA abundance (normalized to nontargeting crRNA) of XIST and BRCA1 in HEK293T cells transfected with all-in-one plasmid expressing Cas/Csm proteins and intron- or exon-targeting crRNAs, measured by RT-qPCR. XIST crRNA 1 and BRCA1 crRNA 2 were used to target XIST and BRCA1 exons, respectively. Intron-targeting crRNAs are listed in Supplementary Table 1. Error bars indicate mean ± s.d. of three biological replicates. **f**, RNA FISH (red) for the indicated targets in HEK293T cells transfected with all-in-one plasmid expressing targeting (T) or nontargeting (NT) crRNA and RNase-active or -inactive (Mut) Cas/Csm proteins. Untransfected cells serve as internal control for transfected (green) cells. XIST crRNA 1, MALAT1 crRNA 1 and NEAT1 crRNA 2 were used to target XIST, MALAT1 and NEAT1, respectively. Scale bar, 10 μm. **g**, Quantification of **f**. One hundred transfected cells were counted for each condition. Error bars indicate mean ± s.d. of three biological replicates.

Thus far, we have only used Csm to KD highly overexpressed, heterologous GFP/RFP transgenes and assayed KD at the protein level (half-life >24 h^56^), which may not accurately reflect abundance at the RNA level. We thus sought to target endogenous transcripts and assay RNA KD directly. We chose to target a panel of three nuclear noncoding RNAs (XIST, MALAT1 and NEAT1) and eight cytoplasmic mRNAs (BRCA1, TARDBP, SMARCA1, CKB, ENO1, MECP2, UBE3A and SMAD4) (Fig. 2a) of varying abundances (Fig. 2b), testing three individual crRNAs for each. HEK293T cells were transfected with all-in-one vector, transfected (RFP-positive) cells were isolated by FACS after 48 h, total cell RNA was extracted and RNA KD was assayed by RT-qPCR (Supplementary Figs. 1c and 2a,c). To our surprise, we achieved >90% KD for all eleven RNAs with at least one crRNA, compared to nontargeting crRNA control (Fig. 2a). We also confirmed multiplexed KD for three of the RNAs (XIST, MALAT1 and NEAT1) (Fig. 2c). These results demonstrate Csm to be a highly robust and efficient RNA KD tool for not only cytoplasmic but also nuclear RNAs, which are typically recalcitrant to KD by conventional RNAi methods^6^.

To examine KD kinetics, we repeated the above RT-qPCR experiment for two of the RNA targets (XIST and BRCA1) across a 5-d time course. KD peaked 2–3 d post transfection and waned thereafter (Fig. 2d), as might be expected from the transient transfection method used to deliver Csm into cells. We also compared KD efficiency of crRNAs targeting intronic versus exonic regions for the same two RNAs (Fig. 2e). Targeting introns did not lead to any noticeable reduction in the mature transcript, possibly because introns are excised from the pre-mRNA more rapidly than they are cleaved by Csm.

To corroborate RNA KD with an orthogonal method, we performed RNA fluorescent in situ hybridization (FISH) for all three nuclear noncoding RNAs, which are easily visualized and display characteristic morphologies. HEK293T cells were transfected with Csm plasmid carrying a GFP reporter (to identify transfected cells) and either a targeting or nontargeting crRNA and assayed by RNA FISH after 48 h (Supplementary Fig. 2b,c). XIST, MALAT1 and NEAT1 were all readily detected when delivering a nontargeting crRNA control (Fig. 2f,g). By contrast, use of a single targeting crRNA abolished all visible signals for each target RNA in transfected (GFP-positive) cells, whereas signal was still detected in untransfected (GFP-negative) cells. For further validation, delivery of targeting crRNA with catalytically inactivated Csm (RNase mut) fully restored the detection of each target RNA. Thus, we demonstrate robust KD of endogenous transcripts using active Csm complexes by both molecular and microscopy-based techniques.

### RNA KD with minimal off-targets or cytotoxicity

**Fig. 3:**
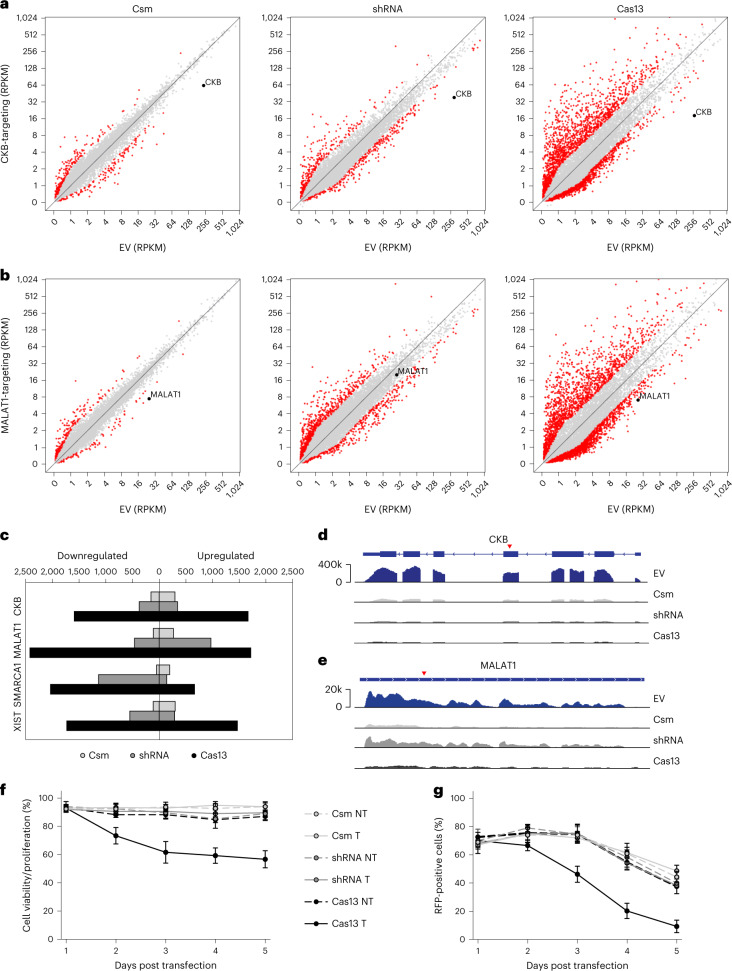
RNA KD with minimal off-targets or cytotoxicity. **a**,**b**, Scatterplots showing differential transcript levels between HEK293T cells transfected with plasmid expressing Csm, Cas13 or shRNA targeting CKB (**a**) or MALAT1 (**b**) versus EV control. Target transcript indicated in black; off-targets (≥2-fold change) indicated in red. **c**, Quantification of upregulated or downregulated transcripts (≥2-fold change) for each sample. CKB crRNA 1, MALAT1 crRNA 2, SMARCA1 crRNA 1 and XIST crRNA 1 were used to target CKB, MALAT1, SMARCA1 and XIST, respectively. **d**,**e**, RNA-seq read coverage across target transcripts CKB (**d**) or MALAT1 (**e**). Red arrow indicates location of crRNA/shRNA target site. **f**, Relative cell viability and proliferation (normalized to EV control) of HEK293T cells at the indicated times post transfection with the indicated targeting (T) or nontargeting (NT) plasmids, measured by WST-1 assay. CKB crRNA 1 was used for targeting. Error bars indicate mean ± s.d. of three biological replicates. **g**, Relative abundance of RFP-positive (transfected) HEK293T cells at the indicated times post transfection with the indicated targeting (T) or nontargeting (NT) plasmids, measured by flow cytometry. CKB crRNA 1 was used for targeting. Error bars indicate mean ± s.d. of three biological replicates.

Next, we performed RNA sequencing (RNA-seq) to examine the potential off-target effects of Csm-mediated KD in cells. For comparison with other established KD technologies, RNA-seq was also performed for Cas13 (RfxCas13d) and RNAi (short hairpin RNA (shRNA))-mediated KD using crRNAs/shRNAs targeting the same complementary sequence^57–59^. KD was performed for 48 h, after which transfected cells were enriched by FACS and sequenced (Supplementary Fig. 3a). Scatterplots comparing transcript levels between nontargeting crRNA and empty vector (EV) control samples for Csm revealed few upregulated or downregulated transcripts (defined as ≥2-fold change, indicated in red) (Supplementary Fig. 3b), suggesting Csm expression itself does not substantially perturb the cellular environment. When targeting CKB, MALAT1, SMARCA1 or XIST, Csm-mediated KD led to significant depletion of the target transcript with few other altered transcripts (Fig. 3a,b and Supplementary Fig. 3c,d). Meanwhile, Cas13 samples showed significant KD of the target transcript while also affecting hundreds of nontarget transcripts. shRNA samples showed variable KD depending on whether the target was cytoplasmic (CKB, SMARCA1) or nuclear (MALAT1, XIST), with an intermediate amount of altered nontarget transcripts. Similar trends were seen for all four targets (Fig. 3c). Examination of RNA-seq read coverage across the target confirmed depletion was transcript-wide and not only localized near the site of Csm cleavage (red arrow), likely due to cellular exonucleotic degradation pathways^60,61^ (Fig. 3d,e and Supplementary Fig 3e,f). We also examined whether Csm-mediated RNA-targeting induces any collateral changes at the DNA level due to its separate DNase activity. DNA-sequencing across the entire *CKB* locus did not reveal any noticeable differences between targeting and nontargeting samples at a sequencing depth of ~1 million reads (Supplementary Fig. 3g). Alternatively, DNase activity can be removed without affecting RNase activity (Fig. 1g)^53^. Hence, Csm-mediated RNA KD shows minimal off-target effects in human cells.

Other RNA-targeting CRISPR-Cas systems such as Cas13 suffer from severe cytotoxic effects due to inherent *trans*-cleavage activity^31–35^. Type III systems do not exhibit *trans*-activity^40^ and are thus poised to offer robust RNA KD without toxicity. To check this, we tracked cell proliferation/viability using the WST-1 assay across a time course after transfecting cells with targeting or nontargeting Csm, Cas13 or shRNA constructs (Fig. 3f). Whereas cells that received targeting Cas13 constructs exhibited a significant decrease in proliferation/viability, those that received Csm or shRNA constructs were unaffected. This decrease in proliferation/viability by WST-1 assay was also seen by a more rapid decrease over time in the proportion of RFP-positive (transfected) cells within the targeting Cas13-treated population compared to the Csm- or shRNA-treated population (Fig. 3g). Taken together, these results suggest that, unlike Cas13, Csm-mediated KD has minimal toxicity in cells.

### Live-cell RNA imaging without genetic manipulation

**Fig. 4:**
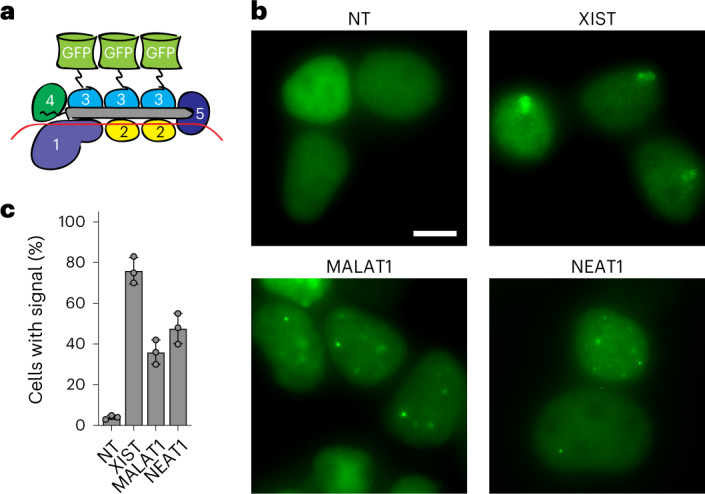
Live-cell RNA imaging without genetic manipulation. **a**, Diagram showing Csm3-GFP fusion complex used for live-cell imaging. **b**, Live-cell fluorescence imaging of HEK293T cells transfected with plasmid expressing Csm3-GFP fusion complex and the indicated crRNAs (Supplementary Table 1). NT, nontargeting. Scale bar, 10 μm. **c**, Quantification of **b**. One hundred transfected cells were counted for each condition. Error bars indicate mean ± s.d. of three biological replicates.

Tracking RNA in live cells remains a difficult task, often requiring genetic insertion of aptamer sequences into the target, which is both laborious and potentially disruptive to RNA function and/or regulation^62^. Fluorescently tagged programmable RNA-binding proteins such as catalytically inactivated Cas13 have recently been adopted for such purposes^17,63,64^. We asked whether the Csm complex could similarly be used to track RNA targets in live cells. To test this, we fused GFP to catalytically inactivated Csm3 (Fig. 4a), the most abundant Csm subunit (≥3 per complex), thereby allowing multivalent display. To visualize XIST RNA, we targeted a repetitive region with a single crRNA predicted to bind eight times per transcript, allowing increased signal. HEK293T cells were transfected with Csm-GFP plasmid and assayed by live-cell fluorescence microscopy after 48 h (Supplementary Fig. 4a). Whereas a nontargeting control crRNA led to only background nuclear fluorescence, the XIST-targeting crRNA led to a strong cloud-like signal in most cells (Fig. 4b,c), phenocopying what we previously observed by XIST RNA FISH (Fig. 2f). Using the same approach, we were able to visualize MALAT1 and NEAT1 transcripts, even with crRNAs predicted to bind only once per target (Fig. 4b,c). Multiplexing several crRNAs against the same target may further improve signal over background, especially for lower abundance transcripts. Thus, fluorescently tagged Csm can be used for easy visualization of RNA in living cells.

## Discussion

Here we have shown that the type III-A Csm complex from *S. thermophilus* is a powerful tool for eukaryotic RNA KD. Both nuclear noncoding RNAs and cytoplasmic mRNAs were able to be knocked down with high efficiency (90–99%) and specificity (~10-fold fewer off-targets than Cas13), outperforming competing RNA KD technologies. More notably, KD was not accompanied by detectable cytotoxicity, unlike Cas13-based methods that suffer from inherent *trans*-cleavage activity^31–33,35^.

Recently, *St*Csm was shown to be effective at depleting GFP or viral RNA upon delivery of bacterially purified RNP into zebrafish embryos or human cells, respectively^53,54^. While demonstrating proof of principle, RNP delivery of multisubunit CRISPR-Cas effectors is not ideal for several reasons as follows: (1) it is often difficult and short-lived compared to DNA-delivery methods, (2) the RNP may be unstable and prone to disassembly and (3) for every new crRNA, the entire RNP must be repurified from bacteria or reconstituted from individually purified subunits in the proper ratio. We have overcome these hurdles of multicomponent CRISPR-Cas systems by encoding all necessary parts in a single deliverable plasmid.

More recently, a single-protein type III effector, Cas7–11, was characterized and used for RNA KD in eukaryotes^32^. This effector is interesting from an evolutionary and structural standpoint in that it appears to have arisen from fusion of the canonical type III subunits into one large polypeptide. While simpler to introduce into eukaryotes, Cas7–11’s demonstrated RNA KD efficiency was only 25–75% for most targets (without enriching for transfected cells), making it somewhat less practical as a tool. Meanwhile, two new reports of naturally occurring and engineered high-fidelity Cas13 variants claim to have mitigated *trans-*cleavage activity (and thus cytotoxicity) while preserving on-target activity^35,57^—although a mechanistic explanation for this remains unclear. Cas7–11 and high-fidelity Cas13s await further characterization before widespread use.

A key advantage of our approach over RNAi is the ability to target transcripts in the nucleus. We were able to achieve >95% KD for three biologically significant nuclear ncRNAs (XIST, MALAT1 and NEAT1). Nuclear RNAs are notoriously difficult to KD, often requiring expensive chemically modified antisense oligos to direct RNase H-mediated cleavage^6^. However, the increased stability of these oligos often leads to unexpected off-target hybridization and cytotoxic effects. Aside from long ncRNAs, nuclear targeting may prove useful for the study of other ncRNA species such as eRNAs, tRNAs, rRNAs, circRNAs, miRNAs and snoRNAs. For instance, it will be interesting to see whether targeting introns containing miRNA or snoRNA clusters enables their degradation before processing/maturation, or whether targeting particular exons alters the abundance of mRNA splice isoforms.

Another advantage of our system is its ease of multiplexing. Multiple spacers can be cloned into the CRISPR array and processed into individual crRNAs by Cas6. This allows for pooled screening, either by encoding crRNAs against multiple targets at once or encoding multiple crRNAs against the same target. The latter may enable robust KD on the first try without the need to individually screen multiple crRNAs against a target. An unexpected observation was the titratable nature of KD with increasing spacer length. This may allow for easy tunability of KD (rather than all-or-none) when studying concentration-dependent effects of gene products.

Csm-mediated RNA KD appears to be robust. We were able to achieve significant KD for nearly all targets tested, with at least one of three crRNAs per target yielding >90% KD. Because, like other RNA-targeting CRISPR-Cas systems, Csm does not have any PAM requirement for target site selection, the only criteria we used were that the target be a unique sequence in the human transcriptome and the spacer avoid stretches of ≥5 consecutive Ts, which might cause premature Pol III transcriptional termination within the crRNA sequence^65^. The observed variability in KD efficiency from one crRNA to another may in part be explained by differences in target site accessibility due to local RNA secondary structure or protein occupancy^66^. A more large-scale analysis must be performed to determine optimal spacer design criteria, and to test how different factors (for example, melting temperature, GC content and target site availability) influence KD efficiency.

We showed that fluorescently tagged, catalytically inactivated Csm can be used for live-cell RNA visualization. By fusing GFP to the most abundant subunit (Csm3), we were able to achieve multivalent display (≥3x GFP per complex), which may offer unique advantages over single-subunit effectors such as Cas13. Beyond GFP, other proteins of interest may be fused to the various Csm subunits to achieve assembly or tethering at a desired stoichiometric ratio. Thus, as a multisubunit complex, Csm offers the benefits of split-protein systems without the engineering effort. Catalytically inactivated Csm might also be useful for disrupting RNA structural motifs or RNA-protein interactions without manipulation at the DNA level.

Finally, this work utilized only the RNase activity of Csm while ignoring its DNase and cA synthase activities. In prokaryotes, cA signaling appears to be the main defensive strategy employed by type III systems^67^, leading to the activation of various downstream effectors^50,51^. These effectors range from RNases to DNases, proteases and transcription factors^68–71^. cA molecules and reliant pathways are currently not known to exist in eukaryotes and thus could be introduced in an orthogonal manner. By bringing type III systems to eukaryotes, we have paved the way for co-introduction of related *trans*-effectors that can be activated in an RNA sequence-dependent manner (Supplementary Fig. 4b). This has important implications for the development of RNA diagnostics, screens and synthetic circuits in vivo.

## Methods

### Cell lines and culture conditions

HEK293T, HEK293T-GFP and HEK293T-GFP/RFP cells (UC Berkeley Cell Culture Facility) were grown in medium containing DMEM, high glucose, GlutaMAX supplement, sodium pyruvate (Thermo Fisher Scientific), 10% FBS (Sigma), 25 mM HEPES pH 7.2–7.5 (Thermo Fisher Scientific), 1× MEM nonessential amino acids (Thermo Fisher Scientific), 1× Pen/Strep (Thermo Fisher Scientific) and 0.1 mM βME (Thermo Fisher Scientific) at 37 °C with 5% CO_2_. All cell lines were verified to be mycoplasma-free (abm, PCR mycoplasma detection kit).

### Plasmid construction and cloning

CRISPR-Cas/Csm sequences were derived from *S. thermophilus* strain ND03 (NCBI). Protein sequences were human codon optimized using online tools (GenScript), synthesized as gene blocks (IDT), modified using PCR and cloned into custom eukaryotic expression vectors (derived from pUC19) by Golden Gate assembly, Gibson assembly (NEB), or Gibson assembly Ultra (Synthetic Genomics). Plasmids were verified by Sanger or whole-plasmid sequencing. All cloning was performed in Stbl3 *Escherichia coli* (Thermo Fisher Scientific) to prevent recombination between repetitive sequences. crRNA/shRNA sequences are provided in Supplementary Table 1. Plasmid sequences are provided in Supplementary Table 2.

### DNA transfections

According to the manufacturer’s instructions, 1 × 10^6^ HEK293T cells were transfected with 2.5 μg plasmid DNA using 7.5 μl FuGENE HD transfection reagent in 6-well plates. Following transfection, cells were grown for 48 h to allow plasmid expression and RNA KD to occur, unless otherwise stated.

### Flow cytometry

Cell fluorescence was assayed on an Attune NxT acoustic focusing cytometer (Thermo Fisher Scientific) equipped with 488 nm excitation laser and 530/30 emission filter (eGFP), and 561 nm excitation laser and 620/15 emission filter (mCherry). Data were analyzed using Attune Cytometric Software v5.1.1 and FlowJo v10.7.1.

### FACS

Cells were sorted by fluorescence on a Sony Cell Sorter SH800Z (100 μm sorting chip) equipped with 488 nm excitation laser and 525/50 emission filter (eGFP), and 561 nm excitation laser and 600/60 emission filter (mCherry). Data were analyzed using Sony Cell Sorter Software v2.1.5.

### RT-qPCR

Total cell RNA was extracted using TRIzol Reagent (Thermo Fisher Scientific) as per the manufacturer’s instructions. Genomic DNA was removed using TURBO DNase (Thermo Fisher Scientific). After inactivating TURBO DNase with DNase Inactivating Reagent, 1 μg DNase-free RNA was reverse transcribed using SuperScript III Reverse Transcriptase (Thermo Fisher Scientific) with random primers (Promega) as per manufacturer’s instructions. qPCR was performed using iTaq Universal SYBR Green Supermix (Bio-Rad) in a CFX96 Real-Time PCR Detection System (Bio-Rad). Gene-specific primer pairs used to detect mature transcripts are listed in Supplementary Table 1. Relative amount of a given target RNA under targeting versus nontargeting conditions was calculated using the formula 2^-((Ct_Target_–Ct_GAPDH_)_Targeting crRNA_ – (Ct_Target_–Ct_GAPDH_)_NT crRNA_). No-RT and no-template controls were run alongside all RT-qPCR experiments.

### Cell viability and proliferation assay

The WST-1 assay was used to quantify cell viability and proliferation. Cells transfected with Csm, Cas13 or shRNA plasmids were grown in 96-well plates until the indicated timepoints, incubated with WST-1 reagent (Sigma) at 37 °C for 1 h as per manufacturer’s instructions, and absorbance measured using a Cytation five microplate reader (BioTek Instruments) at 450 nm with 600 nm reference.

### Microscopy

For wide-field fluorescent imaging, cells were observed on a Zeiss Axio Observer Z1 inverted fluorescence microscope, equipped with 63/1.4 NA oil DIC and 100×/1.4 NA oil Ph3 Plan Apochromat objective lenses, ORCA-Flash4.0 camera (Hamamatsu) and ZEN 2012 software. Images represent max-intensity z-projections and were generated using ZEN 2012 (Zeiss) and FIJI (ImageJ) software. For live-cell imaging, cells were grown on chambered 1.5 coverglasses (Nunc Lab-Tek 2) in medium lacking phenol red (Thermo Fisher Scientific) and imaged directly on the inverted fluorescent microscope.

### RNA FISH

Cells were grown on glass coverslips and rinsed in PBS. They were fixed in 4% paraformaldehyde for 10 min at room temp and then permeabilized in PBS/0.5% Triton X-100 for 10 min. Cells were dehydrated in a series of 70%, 80%, 90% and 100% ethanol for 5 min each. Labeled oligo probe pool (10 nM final) was added to hybridization buffer containing 25% formamide, 2× SSC, 10% dextran sulfate and nonspecific competitor (0.1 mg ml^−^^1^ human Cot-1 DNA (Thermo Fisher Scientific)). Hybridization was performed in a humidified chamber at 37 °C overnight. After being washed once in 25% formamide/2× SSC at 37 °C for 20 min and three times in 2× SSC at 37 °C for 5 min each, cells were mounted for wide-field fluorescent imaging. Nuclei were counter-stained with Hoechst 33342 (Life Technologies).

### FISH probes

XIST oligo FISH probes were designed against the ‘Repeat D’ region of human XIST RNA and synthesized by IDT carrying a 5′ Cy3 dye modification (see Supplementary Table 1 for sequences). MALAT1 and NEAT1 oligo FISH probes were ordered from LGC Biosearch Technologies (SMF-2035-1, SMF-2036-1) carrying a Quasar 570 dye modification.

### Immunofluorescence

Cells were grown on glass coverslips and rinsed in PBS. They were fixed in 4% paraformaldehyde for 10 min and then permeabilized in PBS/0.5% Triton X-100 for 10 min at room temp. Cells were blocked with blocking buffer (PBS/0.05% Tween-20 containing 1% BSA) for 1 h, incubated with primary antibody in blocking buffer for 1 h, washed three times with PBS/0.05% Tween-20 for 5 min each, incubated with dye-conjugated secondary antibody in blocking buffer for 1 h at room temp and washed three times again with PBS/0.05% Tween-20 for 5 min each. Cells were mounted for wide-field fluorescent imaging and nuclei were counter-stained with Hoechst 33342 (Life Technologies).

### Western blot

Cells were washed once with PBS and lysed in cold RIPA lysis buffer (50 mM Tris pH 7.5, 150 mM NaCl, 1% NP-40, 1% sodium deoxycholate, 0.1% SDS, 1× protease inhibitor cocktail (Sigma)). Lysate was sonicated (Qsonica Q800 Sonicator) in polystyrene tubes at 50% power setting, 30 s on/30 s off for a total sonication time of 5 min at 4 ºC. After removing debris by centrifugation at 16,000*g* for 10 min, protein concentration in the supernatant was measured (Pierce BCA Assay Kit). 20–50 μg protein lysate was denatured in 1× Laemmli buffer at 95 ºC for 10 min and resolved by SDS-PAGE. Protein was transferred to Immun-Blot LF PVDF membrane (Bio-Rad). The membrane was blocked with blocking buffer (PBS/0.05% Tween-20 containing 5% milk) for 1 h at room temp, incubated with primary antibody in blocking buffer overnight at 4 ºC, washed three times with PBS/0.05% Tween-20 for 5 min each, incubated with dye-conjugated secondary antibody in blocking buffer for 1 h at room temp and washed three times again with PBS/0.05% Tween-20 for 5 min each. Protein bands were visualized on an LI-COR Odyssey CLx with Image Studio v5.2 software using 700 nm and 800 nm channels.

### Antibodies

For Western blot, the following primary antibodies were used: mice anti-FLAG (Sigma, F1804, Lot SLCC6485; 1:2,000 dilution) and rabbit anti-GAPDH (Cell Signaling Technology, 14C10, Lot 14, 1:5,000 dilution); the following secondary antibodies were used: IRDye 680RD goat antimouse (LI-COR, 926-68070, Lot C90910-21; 1:20,000 dilution), IRDye 800CW goat antirabbit (LI-COR, 926-32211, Lot C90723-19; 1:20,000 dilution). For immunofluorescence, the following primary antibodies were used: mice anti-FLAG (Sigma, F1804, Lot SLCC6485; 1:500 dilution); the following secondary antibodies were used: Alexa Fluor 555 goat antimouse (Invitrogen, A21424, Lot 2123594; 1:2,000 dilution).

### DNA-seq

Cells were lysed with Laird lysis buffer (10 mM Tris pH 8, 5 mM EDTA pH 8, 200 mM NaCl, 0.2% SDS, 0.2 mg ml^−1^ proteinase K) at 55 °C for 2 h and genomic DNA extracted with phenol-chloroform. The *CKB* locus was amplified from genomic DNA by PCR (primer sequences listed in Supplementary Table 1) using PrimeSTAR GXL DNA polymerase (Takara Bio). The full-length PCR amplicon was purified from agarose gel and sheared by sonication to 200–400 bp fragments using a Qsonica Q800 Sonicator at 50% power setting, 30 s on/30 s off, for a total sonication time of 8 min at 4 °C. DNA libraries were prepared using NEBNext Ultra II DNA Library Prep Kit for Illumina, as per manufacturer’s instructions. Libraries were sequenced in-house (Center for Translational Genomics, UC Berkeley) on an iSeq100 with a 150 bp paired-end run configuration to a depth of ~1 million reads each, with one biological replicate per sample.

### DNA-seq analysis

Reads were aligned to the *CKB* (ENSG00000166165) gene locus with BWA MEM (v0.7.17) and PCR duplicates were removed with Picard Tools (v2.21.9). Mismatches and indels at each position were tabulated with Pysamstats (v1.1.2).

### RNA-seq

Total cell RNA was extracted using TRIzol Reagent (Thermo Fisher Scientific). PolyA mRNA was isolated using NEBNext PolyA mRNA Isolation Module (NEB), as per the manufacturer’s instructions. Strand-specific cDNA libraries were prepared using NEBNext Ultra II Directional RNA Library Prep Kit for Illumina, as per the manufacturer’s instructions. Libraries were sequenced by Novogene, Genewiz or UCSF Center for Advanced Technology on a NovaSeq6000 with a 150 bp paired-end run configuration to a depth of ~30 million reads each, with one biological replicate per sample.

### RNA-seq analysis

Reads were assessed for sequencing quality with FastQC (v0.11.9), then adapters and low-quality bases were trimmed with CutAdapt (v4.1). Samples were aligned to the GRCh38 reference genome (GENCODE Release 39) with STAR (v2.7.10a) and uniquely mapped reads were used to generate a strand-specific count matrix with featureCounts (v2.0.3). EdgeR (v3.36.0) calcNormFactors was used to normalize samples according to seven housekeeping genes (UBC, HMBS, TBP, GAPDH, HPRT1, RPL13A and ACTB) before calculating RPKM. Transcripts with a ≥2-fold change relative to EV control were considered upregulated or downregulated.

### Statistical analysis

All graphs display the mean and standard deviation of three biological replicates. For RNA-seq analysis, no statistical parameters were applied given one biological replicate.

### Reporting summary

Further information on research design is available in the Nature Portfolio Reporting Summary linked to this article.

## Online content

Any methods, additional references, Nature Portfolio reporting summaries, source data, extended data, supplementary information, acknowledgements, peer review information; details of author contributions and competing interests; and statements of data and code availability are available at 10.1038/s41587-022-01649-9.

## Supplementary information

Supplementary Information Supplementary Figs. 1–4 and Supplementary Tables 1 and 2.

Reporting Summary

## Data Availability

RNA-seq and DNA-seq datasets have been deposited at GEO (accession number GSE220741)^73^ and SRA (accession number PRJNA911336)^74^, respectively. Essential plasmids have been deposited at Addgene (plasmid ID numbers 195237–195242). Microscope image files have been deposited at figshare (10.6084/m9.figshare.21714815.v1). The GRCh38 reference genome is publicly available (GENCODE Release 39). Source data are provided with this paper, including unprocessed blots.

## Source data

Source Data Fig. 1 Unprocessed western blots.

## Code availability

All codes are available upon request.
