# Supplementary material for: Precise transcript targeting by CRISPR-Csm complexes
Source: Nat Biotechnol. 2023 Jan 23;41(9):1256–64. doi: 10.1038/s41587-022-01649-9 (PMC10497410; doi:10.1038/s41587-022-01649-9)
Supplement: Supplementary file 1 — Supplementary Figs. 1–4 and Supplementary Tables 1 and 2. [file 41587_2022_1649_MOESM1_ESM.pdf]

nature biotechnology

Article https://doi.org/10.1038/s41587-022-01649-9

Precise transcript targeting by CRISPR-Csm complexes

In the format provided by the authors and unedited

Supplementary Information

Precise transcript targeting by CRISPR-Csm complexes

David Colognori, Marena Trinidad, and Jennifer A. Doudna

Supplementary Figures:

Supplementary Figure 1. Additional information regarding flow cytometry and FACS experiments.

Supplementary Figure 2. Additional information regarding RT-qPCR and RNA FISH experiments.

Supplementary Figure 3. Additional information regarding RNA-sequencing experiments.

Supplementary Figure 4. Additional information regarding live-cell RNA imaging experiments.

Supplementary Tables:

Supplementary Table 1. qPCR primer, crRNA, shRNA, and FISH probe sequences.

Supplementary Table 2. Plasmid sequences.

a

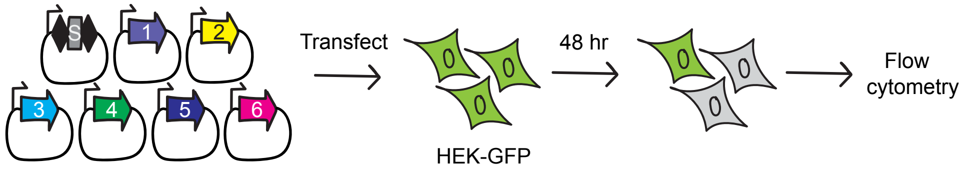
S
1
2
3
4
5
6
Transfect
HEK-GFP
48 hr
Flow cytometry

b

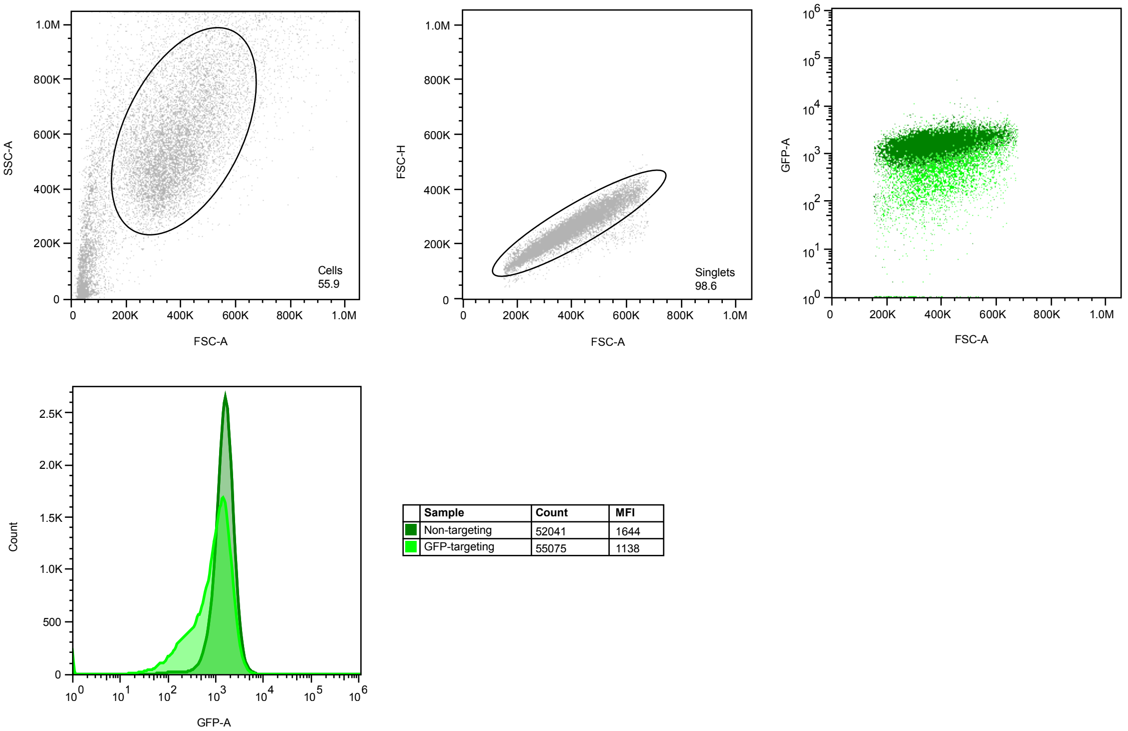
SSC-A
FSC-A
Cells 55.9
FSC-H
Singlets 98.6
GFP-A
Count
Sample | Count | MFI
Non-targeting | 52041 | 1644
GFP-targeting | 55075 | 1138

c

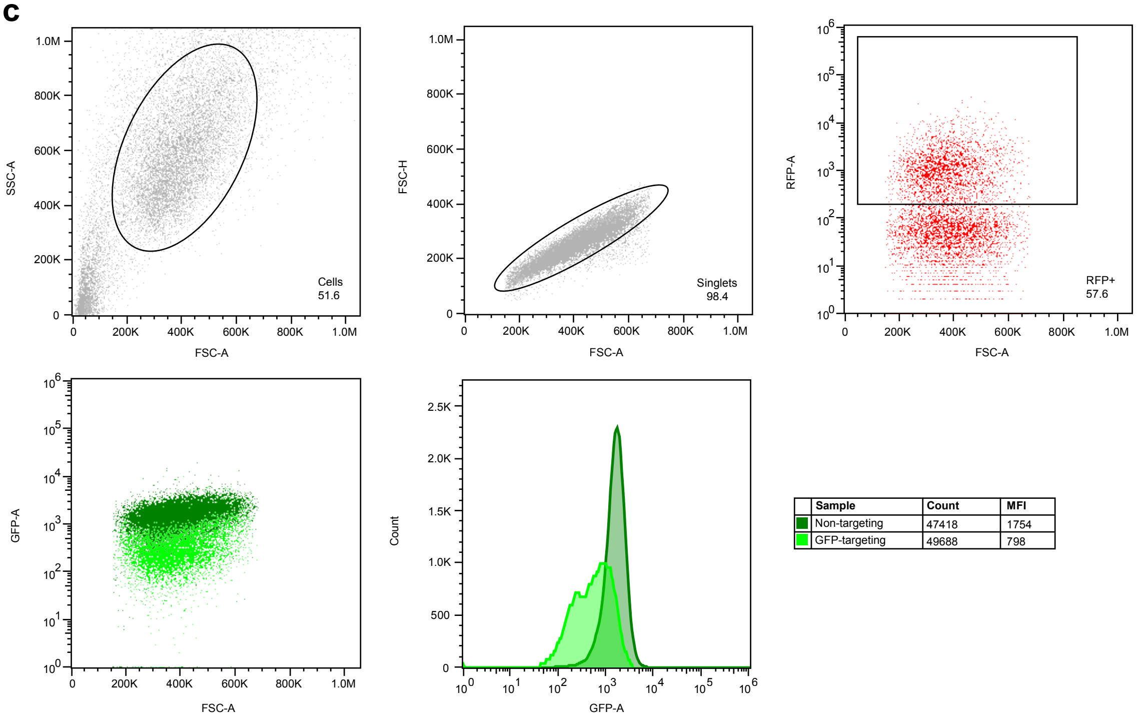
SSC-A
FSC-A
Cells 51.6
FSC-H
Singlets 98.4
RFP-A
RFP+ 57.6
GFP-A
Count
Sample | Count | MFI
Non-targeting | 47418 | 1754
GFP-targeting | 49688 | 798

Supplementary Figure 1. Additional information regarding flow cytometry and FACS experiments.

a, Diagram showing workflow for flow cytometry experiments. Delivery plasmids and recipient cell lines are indicated in each experiment. **b**, Diagram showing gating strategy for flow cytometry experiments. **c**, Diagram showing gating strategy for flow cytometry and FACS experiments in which transfected (RFP-positive) cells were enriched.

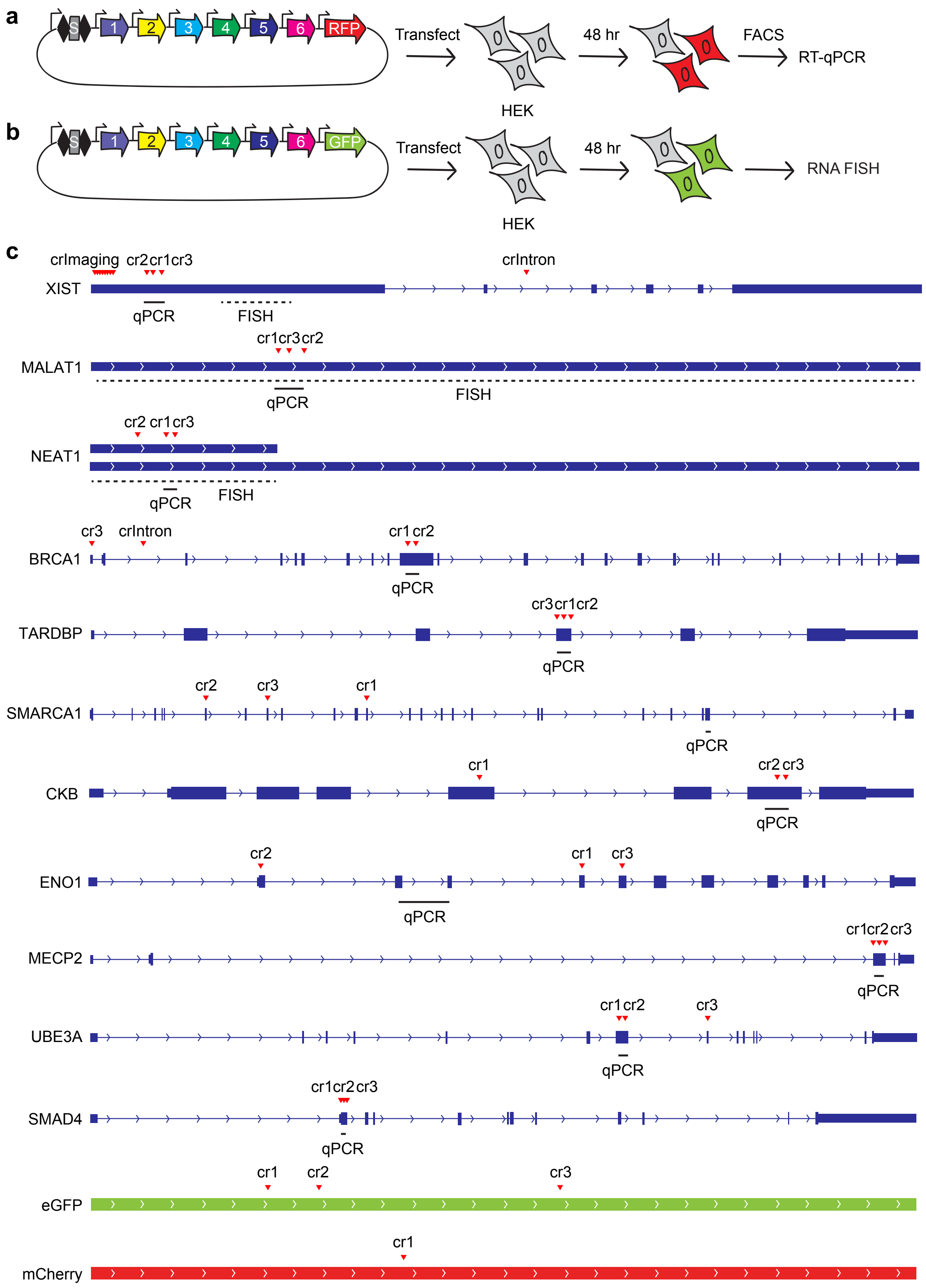
a
S
1
2
3
4
5
6
RFP
Transfect
HEK
48 hr
FACS
RT-qPCR
b
S
1
2
3
4
5
6
GFP
Transfect
HEK
48 hr
RNA FISH
c
crImaging
cr2 cr1 cr3
crIntron
XIST
qPCR
FISH
cr1 cr3 cr2
MALAT1
qPCR
FISH
cr2 cr1 cr3
NEAT1
qPCR
FISH
cr3
crIntron
cr1 cr2
BRCA1
qPCR
cr3 cr1 cr2
TARDBP
qPCR
cr2
cr3
cr1
SMARCA1
qPCR
cr1
cr2 cr3
CKB
qPCR
cr2
cr1
cr3
ENO1
qPCR
cr1 cr2 cr3
MECP2
qPCR
cr1 cr2
cr3
UBE3A
qPCR
cr1 cr2 cr3
SMAD4
qPCR
cr1
cr2
cr3
eGFP
cr1
mCherry

Supplementary Figure 2. Additional information regarding RT-qPCR and RNA FISH experiments.

a, Diagram showing workflow for RT-qPCR experiments. **b**, Diagram showing workflow for RNA FISH experiments. **c**, Diagram showing location of crRNA target site (red arrow), qPCR amplicon (solid black line), and FISH probe (dashed black line) for each transcript. Transcripts are shown 5’ to 3’, with thinner blocks representing UTR regions, thicker blocks representing coding regions, and lines representing intronic regions. Transcripts not to scale.

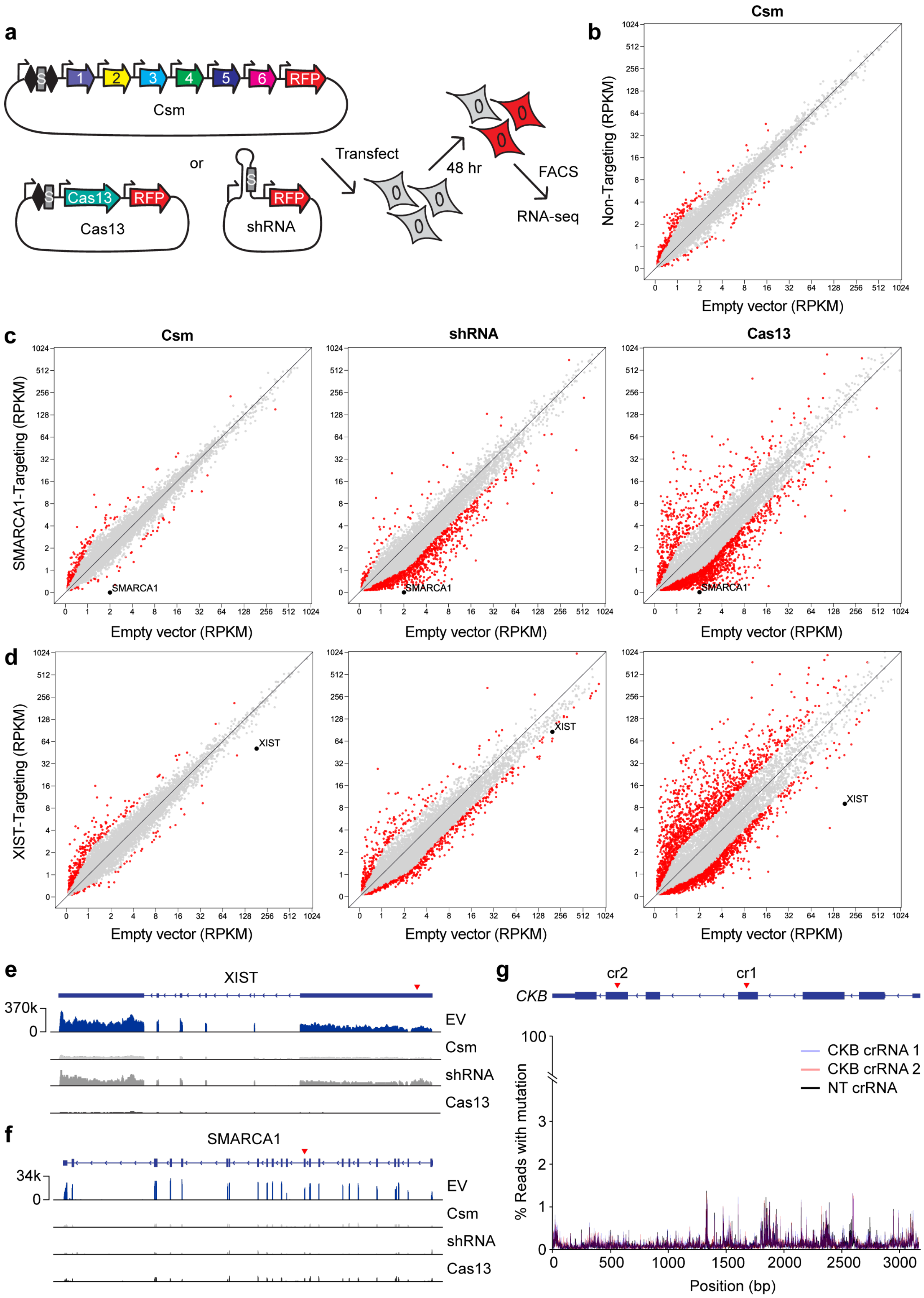

a
S
1
2
3
4
5
6
RFP
Csm
or
Cas13
shRNA
Transfect
48 hr
FACS
RNA-seq
b
Csm
Non-Targeting (RPKM)
Empty vector (RPKM)
0
1
2
4
8
16
32
64
128
256
512
1024
c
Csm
shRNA
Cas13
SMARCA1-Targeting (RPKM)
SMARCA1
Empty vector (RPKM)
d
XIST-Targeting (RPKM)
XIST
Empty vector (RPKM)
e
XIST
370k
0
EV
Csm
shRNA
Cas13
f
SMARCA1
34k
0
EV
Csm
shRNA
Cas13
g
cr2
cr1
CKB
100
3
2
1
0
% Reads with mutation
CKB crRNA 1
CKB crRNA 2
NT crRNA
0
500
1000
1500
2000
2500
3000
Position (bp)

Supplementary Figure 3. Additional information regarding RNA-sequencing experiments.

a, Diagram showing workflow for RNA-seq experiments. **b**, Scatterplot showing differential transcript levels between HEK293T cells transfected with plasmid expressing Csm with non-targeting crRNA versus empty vector control. Up- or down-regulated transcripts (≥2-fold change) indicated in red. **c**, **d**, Scatterplots showing differential transcript levels between HEK293T cells transfected with plasmid expressing Csm, Cas13, or shRNA targeting SMARCA1 (**b**) or XIST (**c**), versus empty vector control. Target transcript indicated in black; off-targets (≥2-fold change) indicated in red. **e**, **f**, RNA-seq read coverage across target transcripts SMARCA1 (**d**) or XIST (**e**). Red arrow indicates location of crRNA/shRNA target site; EV, empty vector. **g**, Plot showing % reads with mutation compared to reference genome across the *CKB* locus in HEK293T cells transfected with all-in-one plasmid expressing Cas/Csm proteins and the indicated crRNAs, assayed by genomic PCR followed by DNA-seq. Red arrow indicates location of crRNA target site; NT, non-targeting.

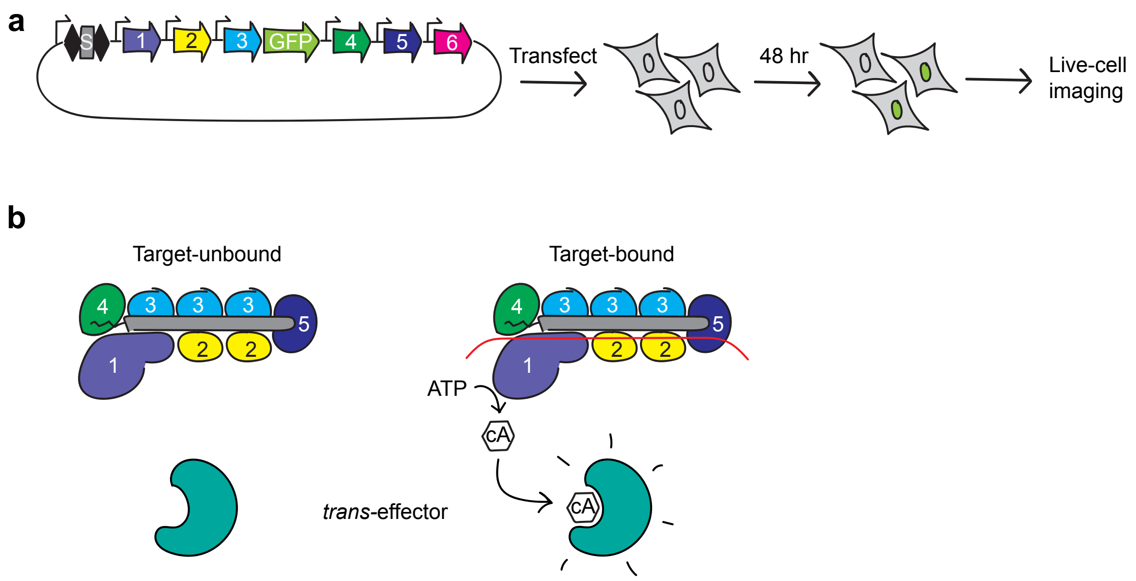
a
S
1
2
3
GFP
4
5
6
Transfect
48 hr
Live-cell imaging
b
Target-unbound
Target-bound
4
3
3
3
5
1
2
2
ATP
cA
trans-effector
cA

Supplementary Figure 4. Additional information regarding live-cell RNA imaging experiments.

a, Diagram showing workflow for live-cell RNA imaging experiments. **b**, Diagram showing target-dependent activation of downstream effectors by the Csm complex.

Supplementary Table 1. qPCR primer, crRNA, shRNA, and FISH probe sequences.

Target	qPCR primer F	qPCR primer R
**XIST**	GTTGTATCGGGAGGCAGTAAGAATCATCTTT	GAAAAGCACACAGCAAAGACAAAGAGGC
**MALAT1**	ACTAGCATTAATTGACAGCTGACCCAGG	GCTACCTTCATCACCAAATTGCACTCG
**NEAT1**	GCTTAGGAGGAGGAAGTTCTCCAATGT	CTCCATCTGCAAGCTCCATCTACAAG
**BRCA1**	TACATCAGGCCTTCATCCTGAGGATTTTATC	ACAATTAGGTGGGCTTAGATTTCTACTGACTACTA
**TARDBP**	GTCAAGAAAGATCTTAAGACTGGTCATTCAAAGGG	CTTAGAATTAGGAAGTTTGCAGTCACACCATC
**SMARCA1**	GATAAACCAGTCAAATCTAAACTGGGGAGCA	GATACAAGGCTCCATTTCATCAGTTGCG
**CKB**	TTAAGCACCTCCGAGAACTTCTCATGC	TTGAAACTCTCTTCAAGTCTAAGGACTATGAGTTCA
**ENO1**	GGAACTCATTAATATACTTAATGGGTCTGGAGACG	CTTCAACTGGTATCTATGAGGCCCTAGAG
**MECP2**	GGAAGAAAAGTCAGAAGACCAGGACC	TTGATCAAATACACATCATACTTCCCAGCAGAG
**UBE3A**	ATATTGATGCCATTAGAAGGGTCTACACCAGAT	CTTTGCAAAATAATGGCAAAGCCATTTCCAG
**SMAD4**	ATGGACAATATGTCTATTACGAATACACCAACAAG TAATG	CTGAAGCCTCCCATCCAATGTTCTCT
**GAPDH**	CCAGAACATCATCCCTGCCTCTACTG	GGAAATGAGCTTGACAAAGTGGTCGTTG

Target	Genomic PCR primer F	Genomic PCR primer R
**CKB**	AATGGAATGAATGGGCTATAAATAGCCGCC	CTTGTCCCATCTCACAGAAGGCGAG

Target	Csm crRNA 1	Csm crRNA 2	Csm crRNA 3
**XIST**	GCCACTTGAACACTGCGACAGA ACTGGATCCG	TTGGACAACCTAACAAAGCACAGC CCGCCATG	CGCACATGTCCACCACCATGCTAA CCACTTAA
**MALAT1**	AGCTTCCTTCACCAAATCGCAC TGGCTCCTGG	GCCGCCTGCTACCTTCATCACCAA ATTGCACT	CCTAGCTTCACCACCAAATCGTTA GCGCTCCT
**NEAT1**	CCGGATGCATCTGCTGTGGACT TTTTAAGATT	CACCATTACCAACAATACCGACTC CAACAGCC	GAAGATGCAGCATCTGAAAACCTT TACCCCAG
**BRCA1**	GGTTAGGATTTTTCTCATTCTG AATAGAATCA	ATTGTGGATATTTAATTCGAGTTC CATATTGC	GAGCAGAGGGTGAAGGCCTCCTGA GCGCAGGG
**TARDBP**	CTTGTGTTTCATATTCCGTAAA ACGAACAAAG	GTCCATCTATCATATGTCGCTGTG ACATTACT	CTTTGAATGACCAGTCTTAAGATC TTTCTTGA
**SMARCA1**	GTCCATCCAGTCGACAATACTC ATAACCACGC	CCGACAAAACAAATGACACGGAGA GATGGGAC	AAAAGTTGAGTAAGGCCCACAGTT CATGCAGG
**CKB**	GCTTGTCGAAGAGGAAGTGGTC GTCGATGAGC	TTGATATGCACACCTGCCCCGCAGC CCGGTGCC	CGAGAACTTCTCATGCTTGCCCAG GTTGGGCA
**ENO1**	CATCCATCTCGATCATCAGTTT GTCAATCTTC	TTCCCGCGAGAGTCAAAGATCTCC CTGGCATG	GACCCCCTTCTCAACGGCACCAGC TTTGCAGA
**MECP2**	CAGAGTGGTGGGCTGATGGCTG CACGGGCTCA	GGCAGAAGCTTCCGGCACAGCCGG GGCGGAGC	CAAATACACATCATACTTCCCAGC AGAGCGGC
**UBE3A**	CTCGAGAGTATACATTGTGATA CGTCAAGTCA	GAGATTTCTATTCTCCATTACGAT AATGAACA	AAATTCCACATACAACTGCTTCTT CAAGTCTG
**SMAD4**	TCCACCTTGTCTATGGCACATC AAACTATGCA	AGCTTCTTTACCAAACTTTCAATT GCTCTTTT	TCCAATGTTCTCTGTATGGTAACA CATTTACT
**GFP**	CAGCTTGCCGGTGGTGCAGATG AACTTCAGGG	TGAAGCACTGCACGCCGTAGGTCA GGGTGGTC	AGGATGTTGCCGTCCTCCTTGAAG TCGATGCC
**RFP**	CTTGAAGCCCTCGGGGAAGGAC AGCTTCAAGT		
**NT**	TCTCCGAACGTGTCACGTCTTT AGCGACTAAA		

Target	Csm crRNA intronic
**XIST**	GTCAGTAAATGAACCTTTCCTATCCCACGTGT
**BRCA1**	CCAGTCATGATCATTCCTGATCACATATTAAG

Target	Cas13 crRNA
**XIST**	GCCACTTGAACACTGCGACAGAACTGGATC
**MALAT1**	GCCGCCTGCTACCTTCATCACCAAATTGCA
**CKB**	GCTTGTCGAAGAGGAAGTGGTCGTCGATGA
**SMAD4**	TCCACCTTGTCTATGGCACATCAAACTATG
**NT**	TCTCCGAACGTGTCACGTCTTTAGCGACTA

**Target**	**shRNA**
**XIST**	GTTCTGTCGCAGTGTTCAAGTcctgacccaACTTGAACACTGCGACAGAAC
**MALAT1**	GTGATGAAGGTAGCAGGCGGcctgacccaGCCGCCTGCTACCTTCATCAC
**CKB**	GACCACTTCCTCTTCGACAAGcctgacccaCTTGTCGAAGAGGAAGTGGTC
**SMAD4**	GATGTGCCATAGACAAGGTGGcctgacccaCCACCTTGTCTATGGCACATC
**NT**	GCTAAAGACGTGACACGTTCGcctgacccaCGAACGTGTCACGTCTTTAGC

**Spacer length**	**Csm crRNA (GFP)**
**24 nt**	CAGCTTGCCGGTGGTGCAGATGAA
**28 nt**	CAGCTTGCCGGTGGTGCAGATGAACTTC
**32 nt**	CAGCTTGCCGGTGGTGCAGATGAACTTCAGGG
**36 nt**	CAGCTTGCCGGTGGTGCAGATGAACTTCAGGGTCAG
**40 nt**	CAGCTTGCCGGTGGTGCAGATGAACTTCAGGGTCAGCTTG
**44 nt**	CAGCTTGCCGGTGGTGCAGATGAACTTCAGGGTCAGCTTGCCGT
**48 nt**	CAGCTTGCCGGTGGTGCAGATGAACTTCAGGGTCAGCTTGCCGTAGGT

**Target**	**Live-cell imaging Csm crRNA**
**XIST**	AAAAGCAGGTATCCGCGGCCCCGATGGGCAAA
**MALAT1**	AGCTTCCTTCACCAAATCGCACTGGCTCCTGG
**NEAT1**	CACCATTACCAACAATACCGACTCCAACAGCC

**Target**	**RNA FISH probe**
**XIST**	/5Cy3/GGGCACTCCCTGCTGGAAGGGAA; /5Cy3/AATTGTGCACCTTGACTGTCCAAA; /5Cy3/TCTGAGAGTAGGACCTTATTCA; /5Cy3/TCAGCACCCCTGCTGTACTGCAAA
**MALAT1**	SMF-2035-1 (LGC Biosearch Technologies)
**NEAT1**	SMF-2036-1 (LGC Biosearch Technologies)

Supplementary Table 2. Plasmid sequences.

**Plasmid**	**pDAC338**
**Description**	Expression of Csm1
**Utility**	
**Features**	Pcmv-FLAG-NLS-Csm1-pA
**Sequence**	ACATGTGAGCAAAAGGCCAGCAAAAGGCCAGGAACCGTAAAAAGGCCGCGTTGCTGGCGTTTTTCCATAGG<br>CTCCGCCCCCCTGACGAGCATCACAAAAATCGACGCTCAAGTCAGAGGTGGCGAAACCCGACAGGACTATA<br>AAGATACCAGGCGTTTCCCCCTGGAAGCTCCCTCGTGCGCTCTCCTGTTCCGACCCTGCCGCTTACCGGAT<br>ACCTGTCCGCCTTTCTCCCTTCGGGAAGCGTGGCGCTTTCTCATAGCTCACGCTGTAGGTATCTCAGTTCG<br>GTGTAGGTCGTTCGCTCCAAGCTGGGCTGTGTGCACGAACCCCCCGTTCAGCCCGACCGCTGCGCCTTATC<br>CGGTAACTATCGTCTTGAGTCCAACCCGGTAAGACACGACTTATCGCCACTGGCAGCAGCCACTGGTAACA<br>GGATTAGCAGAGCGAGGTATGTAGGCGGTGCTACAGAGTTCTTGAAGTGGTGGCCTAACTACGGCTACACT<br>AGAAGAACAGTATTTGGTATCTGCGCTCTGCTGAAGCCAGTTACCTTCGGAAAAAGAGTTGGTAGCTCTTG<br>ATCCGGCAAACAAACCACCGCTGGTAGCGGTGGTTTTTTTGTTTGCAAGCAGCAGATTACGCGCAGAAAAA<br>AAGGATCTCAAGAAGATCCTTTGATCTTTTCTACGGGGTCTGACGCTCAGTGGAACGAAAACTCACGTTAA<br>GGGATTTTGGTCATGAGATTATCAAAAAGGATCTTCACCTAGATCCTTTTAAATTAAAAATGAAGTTTTAA<br>ATCAATCTAAAGTATATATGAGTAAACTTGGTCTGACAGTTACCAATGCTTAATCAGTGAGGCACCTATCT<br>CAGCGATCTGTCTATTTCGTTCATCCATAGTTGCCTGACTCCCCGTCGTGTAGATAACTACGATACGGGAG<br>GGCTTACCATCTGGCCCCAGTGCTGCAATGATACCGCGAGACCCACGCTCACCGGCTCCAGATTTATCAGC<br>AATAAACCAGCCAGCCGGAAGGGCCGAGCGCAGAAGTGGTCCTGCAACTTTATCCGCCTCCATCCAGTCTA<br>TTAATTGTTGCCGGGAAGCTAGAGTAAGTAGTTCGCCAGTTAATAGTTTGCGCAACGTTGTTGCCATTGCT<br>ACAGGCATCGTGGTGTCACGCTCGTCGTTTGGTATGGCTTCATTCAGCTCCGGTTCCCAACGATCAAGGCG<br>AGTTACATGATCCCCCATGTTGTGCAAAAAAGCGGTTAGCTCCTTCGGTCCTCCGATCGTTGTCAGAAGTA<br>AGTTGGCCGCAGTGTTATCACTCATGGTTATGGCAGCACTGCATAATTCTCTTACTGTCATGCCATCCGTA<br>AGATGCTTTTCTGTGACTGGTGAGTACTCAACCAAGTCATTCTGAGAATAGTGTATGCGGCGACCGAGTTG<br>CTCTTGCCCGGCGTCAATACGGGATAATACCGCGCCACATAGCAGAACTTTAAAAGTGCTCATCATTGGAA<br>AACGTTCTTCGGGGCGAAAACTCTCAAGGATCTTACCGCTGTTGAGATCCAGTTCGATGTAACCCACTCGT<br>GCACCCAACTGATCTTCAGCATCTTTTACTTTCACCAGCGTTTCTGGGTGAGCAAAAACAGGAAGGCAAAA<br>TGCCGCAAAAAAGGGAATAAGGGCGACACGGAAATGTTGAATACTCATACTCTTCCTTTTTCAATATTATT<br>GAAGCATTTATCAGGGTTATTGTCTCATGAGCGGATACATATTTGAATGTATTTAGAAAAATAAACAAATA<br>GGGGTTCCGCGCACATTTCCCCGAAAAGTGCCACCTGACGTCGGATCCgacattgattattgactagttat<br>taatagtaatcaattacggggtcattagttcatagcccatatatggagttccgCGTTACATAACTTACGGT<br>AAATGGCCCGCCTGGCTGACCGCCCAACGACCCCCGCCCATTGACGTCAATAATGACGTATGTTCCCATAG<br>TAACGCCAATAGGGACTTTCCATTGACGTCAATGGGTGGAGTATTTACGGTAAACTGCCCACTTGGCAGTA<br>CATCAAGTGTATCATATGCCAAGTACGCCCCCTATTGACGTCAATGACGGTAAATGGCCCGCCTGGCATTA<br>TGCCCAGTACATGACCTTATGGGACTTTCCTACTTGGCAGTACATCTACGTATTAGTCATCGCTATTACCA<br>TGGTGATGCGGTTTTGGCAGTACATCAATGGGCGTGGATAGCGGTTTGACTCACGGGGATTTCCAAGTCTC<br>CACCCCATTGACGTCAATGGGAGTTTGTTTTGGCACCAAAATCAACGGGACTTTCCAAAATGTCGTAACAA<br>CTCCGCCCCATTGACGCAAATGGGCGGTAGGCGTGTACGGTGGGAGGTCTATATAAGCAGAGCTCGTTTAG<br>TGAACCGTCAGATCTCTAGAgccgccaccATGGATTACAAAGACGATGACGATAAGATGGCGCCTAAGAAG<br>AAACGCAAAGTGCGGGGCATGAAGAAAGAAAAGATTGATCTGTTTTACGGAGCCCTGCTGCACGACATCGG<br>AAAGGTCATCCAGCGAGCAACCGGAGAGCGGAAGAAACACGCACTTGTGGGCGCCGACTGGTTCGACGAGA<br>TCGCCGACAACCAAGTCATCTCGGATCAGATCCGGTACCATATGGCCAACTACCAGTCTGATAAGCTCGGC<br>AACGATCACCTGGCTTACATCACCTACATTGCCGACAACATCGCCTCCGGTGTCGACCGCCGGCAATCCAA<br>CGAAGAGTCAGACGAAGATACCTCCGCAAAGATCTGGGACACCTACACGAACCAGGCCGACATCTTTAACG<br>TGTTCGGAGCGCAGACCGATAAGCGGTACTTCAAGCCTACCGTGCTGAATCTCAAGTCGAAGCCCAACTTC<br>GCGTCCGCCACTTACGAACCCTTTAGCAAGGGCGATTACGCTGCCATCGCCACCCGGATTAAGAACGAACT<br>GGCCGAGTTCGAGTTCAACCAAGTCCAGATTGACTCCCTGCTCAACCTTTTCGAGGCTACTCTCTCCTTCG<br>TGCCGTCAAGCACCAACACTAAGGAAATCGCCGACATCTCCCTGGCCGACCATTCCCGCTTGACTGCTGCC<br>TTCGCTCTGGCGATCTACGACTACCTGGAGGACAAGGGTCGGCACAACTACAAAGAGGACCTGTTCACCAA<br>AGTGTCAGCGTTCTATGAAGAAGAAGCCTTCCTGCTGGCCTCCTTCGACCTGTCGGGAATCCAGGACTTTA<br>TCTACAACATTAACATCGCAACTAACGGCGCGGCGAAGCAGCTGAAGGCCCGGAGCCTCTACCTGGACTTT<br>ATGTCCGAGTACATCGCCGATAGCCTGCTGGACAAGCTGGGACTGAACAGGGCTAACATGCTTTACGTCGG<br>CGGCGGACACGCCTACTTCGTCCTGGCCAACACCGAAAAGACTGTGGAAACCCTGGTGCAGTTTGAGAAGG<br>ATTTCAACCAGTTCCTGTTGGCAAACTTCCAGACCCGCCTCTATGTGGCCTTTGGCTGGGGTTCCTTCGCG<br>GCCAAGGACATCATGTCCGAGCTGAATAGCCCCGAGTCCTACCGCCAAGTGTACCAAAAGGCTTCGCGCAT<br>GATCTCCAAAAAGAAAATCTCCAGATACGACTACCAGACACTGATGCTCCTGAATCGCGGTGGAAAGTCCT<br>CAGAGAGAGAGTGCGAGATTTGCCACTCCGTGGAGAACCTGGTGTCCTACCACGACCAGAAAGTCTGTGAC<br>ATTTGCCGGGGACTGTACCAGTTCTCGAAAGAAATTGCCCATGACCACTTCATCATTACCGAAAATGAGGG<br>GCTGCCGATTGGACCAAACGCGTGCTTAAAGGGCGTGGCATTCGAAAAGCTGTCCCAAGAAGCGTTCAGCC<br>GGGTCTACGTGAAGAATGACTATAAGGCCGGTACCGTGAAGGCTACGCATGTGTTCGTGGGGGATTACCAG

	TGCGACGAGATCTACAACTACGCCGCCCTGAGCAAGAACGAGAACGGCCTAGGCATCAAGAGACTGGCCGT<br>GGTCCGGCTCGACGTGGATGACTTGGGCGCCGCCTTCATGGCCGGTTTCAGCCAGCAGGGAAACGGACAAT<br>ACTCCACTCTGTCAAGATCGGCCACATTCTCCCGGAGCATGTCGCTGTTCTTCAAAGTGTACATTAACCAG<br>TTCGCCTCCGACAAGAAGCTGAGCATTATCTACGCGGGCGGCGATGACGTGTTCGCCATTGGATCGTGGCA<br>GGATATCATCGCGTTCACTGTGGAACTTCGCGAAAACTTCATCAAGTGGACCAACGGGAAGCTCACCCTCT<br>CCGCGGGGATAGGGTTGTTCGCCGACAAGACTCCTATTAGCCTGATGGCTCACCAGACCGGGGAACTGGAA<br>GAGGCCGCCAAGGGCAACGAAAAGGACTCCATCTCGCTGTTCTCAAGCGACTACACTTTCAAGTTTGATAG<br>GTTCATCACTAACGTGTACGACGACAAACTGGAACAGATTAGATACTTCTTCAACCATCAAGACGAGAGGG<br>GAAAGAACTTCATCTATAAGCTTATTGAGCTTTTGAGGAACCACGACCGCATGAATATGGCACGCCTCGCC<br>TATTACCTCACTCGCCTGGAAGAACTGACCCGGGAGACTGACAGGGACAAGTTCAAGACCTTCAAGAACCT<br>GTTCTACTCCTGGTACACCAACAAGAACGATAAGGACCGGAAGGAAGCCGAGCTCGCGCTCCTGCTGTACA<br>TCTACGAAATCAGAAAGGATTAAcggcaataaaaagacagaataaaacgcacggtgttgggtcgtttgttc<br>AAGCTC

**Plasmid**	**pDAC803**
**Description**	Expression of Csm1(DNase mut)
**Utility**	
**Features**	Pcmv-FLAG-NLS-Csm1(DNase mut)-pA
**Sequence**	ACATGTGAGCAAAAGGCCAGCAAAAGGCCAGGAACCGTAAAAAGGCCGCGTTGCTGGCGTTTTTCCATAGG<br>CTCCGCCCCCCTGACGAGCATCACAAAAATCGACGCTCAAGTCAGAGGTGGCGAAACCCGACAGGACTATA<br>AAGATACCAGGCGTTTCCCCCTGGAAGCTCCCTCGTGCGCTCTCCTGTTCCGACCCTGCCGCTTACCGGAT<br>ACCTGTCCGCCTTTCTCCCTTCGGGAAGCGTGGCGCTTTCTCATAGCTCACGCTGTAGGTATCTCAGTTCG<br>GTGTAGGTCGTTCGCTCCAAGCTGGGCTGTGTGCACGAACCCCCCGTTCAGCCCGACCGCTGCGCCTTATC<br>CGGTAACTATCGTCTTGAGTCCAACCCGGTAAGACACGACTTATCGCCACTGGCAGCAGCCACTGGTAACA<br>GGATTAGCAGAGCGAGGTATGTAGGCGGTGCTACAGAGTTCTTGAAGTGGTGGCCTAACTACGGCTACACT<br>AGAAGAACAGTATTTGGTATCTGCGCTCTGCTGAAGCCAGTTACCTTCGGAAAAAGAGTTGGTAGCTCTTG<br>ATCCGGCAAACAAACCACCGCTGGTAGCGGTGGTTTTTTTGTTTGCAAGCAGCAGATTACGCGCAGAAAAA<br>AAGGATCTCAAGAAGATCCTTTGATCTTTTCTACGGGGTCTGACGCTCAGTGGAACGAAAACTCACGTTAA<br>GGGATTTTGGTCATGAGATTATCAAAAAGGATCTTCACCTAGATCCTTTTAAATTAAAAATGAAGTTTTAA<br>ATCAATCTAAAGTATATATGAGTAAACTTGGTCTGACAGTTACCAATGCTTAATCAGTGAGGCACCTATCT<br>CAGCGATCTGTCTATTTCGTTCATCCATAGTTGCCTGACTCCCCGTCGTGTAGATAACTACGATACGGGAG<br>GGCTTACCATCTGGCCCCAGTGCTGCAATGATACCGCGAGACCCACGCTCACCGGCTCCAGATTTATCAGC<br>AATAAACCAGCCAGCCGGAAGGGCCGAGCGCAGAAGTGGTCCTGCAACTTTATCCGCCTCCATCCAGTCTA<br>TTAATTGTTGCCGGGAAGCTAGAGTAAGTAGTTCGCCAGTTAATAGTTTGCGCAACGTTGTTGCCATTGCT<br>ACAGGCATCGTGGTGTCACGCTCGTCGTTTGGTATGGCTTCATTCAGCTCCGGTTCCCAACGATCAAGGCG<br>AGTTACATGATCCCCCATGTTGTGCAAAAAAGCGGTTAGCTCCTTCGGTCCTCCGATCGTTGTCAGAAGTA<br>AGTTGGCCGCAGTGTTATCACTCATGGTTATGGCAGCACTGCATAATTCTCTTACTGTCATGCCATCCGTA<br>AGATGCTTTTCTGTGACTGGTGAGTACTCAACCAAGTCATTCTGAGAATAGTGTATGCGGCGACCGAGTTG<br>CTCTTGCCCGGCGTCAATACGGGATAATACCGCGCCACATAGCAGAACTTTAAAAGTGCTCATCATTGGAA<br>AACGTTCTTCGGGGCGAAAACTCTCAAGGATCTTACCGCTGTTGAGATCCAGTTCGATGTAACCCACTCGT<br>GCACCCAACTGATCTTCAGCATCTTTTACTTTCACCAGCGTTTCTGGGTGAGCAAAAACAGGAAGGCAAAA<br>TGCCGCAAAAAAGGGAATAAGGGCGACACGGAAATGTTGAATACTCATACTCTTCCTTTTTCAATATTATT<br>GAAGCATTTATCAGGGTTATTGTCTCATGAGCGGATACATATTTGAATGTATTTAGAAAAATAAACAAATA<br>GGGGTTCCGCGCACATTTCCCCGAAAAGTGCCACCTGACGTCGGATCCgacattgattattgactagttat<br>taatagtaatcaattacggggtcattagttcatagcccatatatggagttccgCGTTACATAACTTACGGT<br>AAATGGCCCGCCTGGCTGACCGCCCAACGACCCCCGCCCATTGACGTCAATAATGACGTATGTTCCCATAG<br>TAACGCCAATAGGGACTTTCCATTGACGTCAATGGGTGGAGTATTTACGGTAAACTGCCCACTTGGCAGTA<br>CATCAAGTGTATCATATGCCAAGTACGCCCCCTATTGACGTCAATGACGGTAAATGGCCCGCCTGGCATTA<br>TGCCCAGTACATGACCTTATGGGACTTTCCTACTTGGCAGTACATCTACGTATTAGTCATCGCTATTACCA<br>TGGTGATGCGGTTTTGGCAGTACATCAATGGGCGTGGATAGCGGTTTGACTCACGGGGATTTCCAAGTCTC<br>CACCCCATTGACGTCAATGGGAGTTTGTTTTGGCACCAAAATCAACGGGACTTTCCAAAATGTCGTAACAA<br>CTCCGCCCCATTGACGCAAATGGGCGGTAGGCGTGTACGGTGGGAGGTCTATATAAGCAGAGCTCGTTTAG<br>TGAACCGTCAGATCTCTAGAgccgccaccATGGATTACAAAGACGATGACGATAAGATGGCGCCTAAGAAG<br>AAACGCAAAGTGCGGGGCATGAAGAAAGAAAAGATTGATCTGTTTTACGGAGCCCTGCTGGCCGCCATCGG<br>AAAGGTCATCCAGCGAGCAACCGGAGAGCGGAAGAAACACGCACTTGTGGGCGCCGACTGGTTCGACGAGA<br>TCGCCGACAACCAAGTCATCTCGGATCAGATCCGGTACCATATGGCCAACTACCAGTCTGATAAGCTCGGC<br>AACGATCACCTGGCTTACATCACCTACATTGCCGACAACATCGCCTCCGGTGTCGACCGCCGGCAATCCAA<br>CGAAGAGTCAGACGAAGATACCTCCGCAAAGATCTGGGACACCTACACGAACCAGGCCGACATCTTTAACG<br>TGTTCGGAGCGCAGACCGATAAGCGGTACTTCAAGCCTACCGTGCTGAATCTCAAGTCGAAGCCCAACTTC<br>GCGTCCGCCACTTACGAACCCTTTAGCAAGGGCGATTACGCTGCCATCGCCACCCGGATTAAGAACGAACT<br>GGCCGAGTTCGAGTTCAACCAAGTCCAGATTGACTCCCTGCTCAACCTTTTCGAGGCTACTCTCTCCTTCG<br>TGCCGTCAAGCACCAACACTAAGGAAATCGCCGACATCTCCCTGGCCGACCATTCCCGCTTGACTGCTGCC<br>TTCGCTCTGGCGATCTACGACTACCTGGAGGACAAGGGTCGGCACAACTACAAAGAGGACCTGTTCACCAA

	AGTGTCAGCGTTCTATGAAGAAGAAGCCTTCCTGCTGGCCTCCTTCGACCTGTCGGGAATCCAGGACTTTA<br>TCTACAACATTAACATCGCAACTAACGGCGCGGCGAAGCAGCTGAAGGCCCGGAGCCTCTACCTGGACTTT<br>ATGTCCGAGTACATCGCCGATAGCCTGCTGGACAAGCTGGGACTGAACAGGGCTAACATGCTTTACGTCGG<br>CGGCGGACACGCCTACTTCGTCCTGGCCAACACCGAAAAGACTGTGGAAACCCTGGTGCAGTTTGAGAAGG<br>ATTTCAACCAGTTCCTGTTGGCAAACTTCCAGACCCGCCTCTATGTGGCCTTTGGCTGGGGTTCCTTCGCG<br>GCCAAGGACATCATGTCCGAGCTGAATAGCCCCGAGTCCTACCGCCAAGTGTACCAAAAGGCTTCGCGCAT<br>GATCTCCAAAAAGAAAATCTCCAGATACGACTACCAGACACTGATGCTCCTGAATCGCGGTGGAAAGTCCT<br>CAGAGAGAGAGTGCGAGATTTGCCACTCCGTGGAGAACCTGGTGTCCTACCACGACCAGAAAGTCTGTGAC<br>ATTTGCCGGGGACTGTACCAGTTCTCGAAAGAAATTGCCCATGACCACTTCATCATTACCGAAAATGAGGG<br>GCTGCCGATTGGACCAAACGCGTGCTTAAAGGGCGTGGCATTCGAAAAGCTGTCCCAAGAAGCGTTCAGCC<br>GGGTCTACGTGAAGAATGACTATAAGGCCGGTACCGTGAAGGCTACGCATGTGTTCGTGGGGGATTACCAG<br>TGCGACGAGATCTACAACTACGCCGCCCTGAGCAAGAACGAGAACGGCCTAGGCATCAAGAGACTGGCCGT<br>GGTCCGGCTCGACGTGGATGACTTGGGCGCCGCCTTCATGGCCGGTTTCAGCCAGCAGGGAAACGGACAAT<br>ACTCCACTCTGTCAAGATCGGCCACATTCTCCCGGAGCATGTCGCTGTTCTTCAAAGTGTACATTAACCAG<br>TTCGCCTCCGACAAGAAGCTGAGCATTATCTACGCGGGCGGCGATGACGTGTTCGCCATTGGATCGTGGCA<br>GGATATCATCGCGTTCACTGTGGAACTTCGCGAAAACTTCATCAAGTGGACCAACGGGAAGCTCACCCTCT<br>CCGCGGGGATAGGGTTGTTCGCCGACAAGACTCCTATTAGCCTGATGGCTCACCAGACCGGGGAACTGGAA<br>GAGGCCGCCAAGGGCAACGAAAAGGACTCCATCTCGCTGTTCTCAAGCGACTACACTTTCAAGTTTGATAG<br>GTTCATCACTAACGTGTACGACGACAAACTGGAACAGATTAGATACTTCTTCAACCATCAAGACGAGAGGG<br>GAAAGAACTTCATCTATAAGCTTATTGAGCTTTTGAGGAACCACGACCGCATGAATATGGCACGCCTCGCC<br>TATTACCTCACTCGCCTGGAAGAACTGACCCGGGAGACTGACAGGGACAAGTTCAAGACCTTCAAGAACCT<br>GTTCTACTCCTGGTACACCAACAAGAACGATAAGGACCGGAAGGAAGCCGAGCTCGCGCTCCTGCTGTACA<br>TCTACGAAATCAGAAAGGATTAAcggcaataaaaagacagaataaaacgcacggtgttgggtcgtttgttc<br>AAGCTC

**Plasmid**	**pDAC804**
**Description**	Expression of Csm1(cA mut)
**Utility**	
**Features**	Pcmv-FLAG-NLS-Csm1(cA mut)-pA
**Sequence**	ACATGTGAGCAAAAGGCCAGCAAAAGGCCAGGAACCGTAAAAAGGCCGCGTTGCTGGCGTTTTTCCATAGG<br>CTCCGCCCCCCTGACGAGCATCACAAAAATCGACGCTCAAGTCAGAGGTGGCGAAACCCGACAGGACTATA<br>AAGATACCAGGCGTTTCCCCCTGGAAGCTCCCTCGTGCGCTCTCCTGTTCCGACCCTGCCGCTTACCGGAT<br>ACCTGTCCGCCTTTCTCCCTTCGGGAAGCGTGGCGCTTTCTCATAGCTCACGCTGTAGGTATCTCAGTTCG<br>GTGTAGGTCGTTCGCTCCAAGCTGGGCTGTGTGCACGAACCCCCCGTTCAGCCCGACCGCTGCGCCTTATC<br>CGGTAACTATCGTCTTGAGTCCAACCCGGTAAGACACGACTTATCGCCACTGGCAGCAGCCACTGGTAACA<br>GGATTAGCAGAGCGAGGTATGTAGGCGGTGCTACAGAGTTCTTGAAGTGGTGGCCTAACTACGGCTACACT<br>AGAAGAACAGTATTTGGTATCTGCGCTCTGCTGAAGCCAGTTACCTTCGGAAAAAGAGTTGGTAGCTCTTG<br>ATCCGGCAAACAAACCACCGCTGGTAGCGGTGGTTTTTTTGTTTGCAAGCAGCAGATTACGCGCAGAAAAA<br>AAGGATCTCAAGAAGATCCTTTGATCTTTTCTACGGGGTCTGACGCTCAGTGGAACGAAAACTCACGTTAA<br>GGGATTTTGGTCATGAGATTATCAAAAAGGATCTTCACCTAGATCCTTTTAAATTAAAAATGAAGTTTTAA<br>ATCAATCTAAAGTATATATGAGTAAACTTGGTCTGACAGTTACCAATGCTTAATCAGTGAGGCACCTATCT<br>CAGCGATCTGTCTATTTCGTTCATCCATAGTTGCCTGACTCCCCGTCGTGTAGATAACTACGATACGGGAG<br>GGCTTACCATCTGGCCCCAGTGCTGCAATGATACCGCGAGACCCACGCTCACCGGCTCCAGATTTATCAGC<br>AATAAACCAGCCAGCCGGAAGGGCCGAGCGCAGAAGTGGTCCTGCAACTTTATCCGCCTCCATCCAGTCTA<br>TTAATTGTTGCCGGGAAGCTAGAGTAAGTAGTTCGCCAGTTAATAGTTTGCGCAACGTTGTTGCCATTGCT<br>ACAGGCATCGTGGTGTCACGCTCGTCGTTTGGTATGGCTTCATTCAGCTCCGGTTCCCAACGATCAAGGCG<br>AGTTACATGATCCCCCATGTTGTGCAAAAAAGCGGTTAGCTCCTTCGGTCCTCCGATCGTTGTCAGAAGTA<br>AGTTGGCCGCAGTGTTATCACTCATGGTTATGGCAGCACTGCATAATTCTCTTACTGTCATGCCATCCGTA<br>AGATGCTTTTCTGTGACTGGTGAGTACTCAACCAAGTCATTCTGAGAATAGTGTATGCGGCGACCGAGTTG<br>CTCTTGCCCGGCGTCAATACGGGATAATACCGCGCCACATAGCAGAACTTTAAAAGTGCTCATCATTGGAA<br>AACGTTCTTCGGGGCGAAAACTCTCAAGGATCTTACCGCTGTTGAGATCCAGTTCGATGTAACCCACTCGT<br>GCACCCAACTGATCTTCAGCATCTTTTACTTTCACCAGCGTTTCTGGGTGAGCAAAAACAGGAAGGCAAAA<br>TGCCGCAAAAAAGGGAATAAGGGCGACACGGAAATGTTGAATACTCATACTCTTCCTTTTTCAATATTATT<br>GAAGCATTTATCAGGGTTATTGTCTCATGAGCGGATACATATTTGAATGTATTTAGAAAAATAAACAAATA<br>GGGGTTCCGCGCACATTTCCCCGAAAAGTGCCACCTGACGTCGGATCCgacattgattattgactagttat<br>taatagtaatcaattacggggtcattagttcatagcccatatatggagttccgCGTTACATAACTTACGGT<br>AAATGGCCCGCCTGGCTGACCGCCCAACGACCCCCGCCCATTGACGTCAATAATGACGTATGTTCCCATAG<br>TAACGCCAATAGGGACTTTCCATTGACGTCAATGGGTGGAGTATTTACGGTAAACTGCCCACTTGGCAGTA<br>CATCAAGTGTATCATATGCCAAGTACGCCCCCTATTGACGTCAATGACGGTAAATGGCCCGCCTGGCATTA<br>TGCCCAGTACATGACCTTATGGGACTTTCCTACTTGGCAGTACATCTACGTATTAGTCATCGCTATTACCA<br>TGGTGATGCGGTTTTGGCAGTACATCAATGGGCGTGGATAGCGGTTTGACTCACGGGGATTTCCAAGTCTC<br>CACCCCATTGACGTCAATGGGAGTTTGTTTTGGCACCAAAATCAACGGGACTTTCCAAAATGTCGTAACAA<br>CTCCGCCCCATTGACGCAAATGGGCGGTAGGCGTGTACGGTGGGAGGTCTATATAAGCAGAGCTCGTTTAG

	TGAACCGTCAGATCTCTAGAgccgccaccATGGATTACAAAGACGATGACGATAAGATGGCGCCTAAGAAG<br>AAACGCAAAGTGCGGGGCATGAAGAAAGAAAAGATTGATCTGTTTTACGGAGCCCTGCTGCACGACATCGG<br>AAAGGTCATCCAGCGAGCAACCGGAGAGCGGAAGAAACACGCACTTGTGGGCGCCGACTGGTTCGACGAGA<br>TCGCCGACAACCAAGTCATCTCGGATCAGATCCGGTACCATATGGCCAACTACCAGTCTGATAAGCTCGGC<br>AACGATCACCTGGCTTACATCACCTACATTGCCGACAACATCGCCTCCGGTGTCGACCGCCGGCAATCCAA<br>CGAAGAGTCAGACGAAGATACCTCCGCAAAGATCTGGGACACCTACACGAACCAGGCCGACATCTTTAACG<br>TGTTCGGAGCGCAGACCGATAAGCGGTACTTCAAGCCTACCGTGCTGAATCTCAAGTCGAAGCCCAACTTC<br>GCGTCCGCCACTTACGAACCCTTTAGCAAGGGCGATTACGCTGCCATCGCCACCCGGATTAAGAACGAACT<br>GGCCGAGTTCGAGTTCAACCAAGTCCAGATTGACTCCCTGCTCAACCTTTTCGAGGCTACTCTCTCCTTCG<br>TGCCGTCAAGCACCAACACTAAGGAAATCGCCGACATCTCCCTGGCCGACCATTCCCGCTTGACTGCTGCC<br>TTCGCTCTGGCGATCTACGACTACCTGGAGGACAAGGGTCGGCACAACTACAAAGAGGACCTGTTCACCAA<br>AGTGTCAGCGTTCTATGAAGAAGAAGCCTTCCTGCTGGCCTCCTTCGACCTGTCGGGAATCCAGGACTTTA<br>TCTACAACATTAACATCGCAACTAACGGCGCGGCGAAGCAGCTGAAGGCCCGGAGCCTCTACCTGGACTTT<br>ATGTCCGAGTACATCGCCGATAGCCTGCTGGACAAGCTGGGACTGAACAGGGCTAACATGCTTTACGTCGG<br>CGGCGGACACGCCTACTTCGTCCTGGCCAACACCGAAAAGACTGTGGAAACCCTGGTGCAGTTTGAGAAGG<br>ATTTCAACCAGTTCCTGTTGGCAAACTTCCAGACCCGCCTCTATGTGGCCTTTGGCTGGGGTTCCTTCGCG<br>GCCAAGGACATCATGTCCGAGCTGAATAGCCCCGAGTCCTACCGCCAAGTGTACCAAAAGGCTTCGCGCAT<br>GATCTCCAAAAAGAAAATCTCCAGATACGACTACCAGACACTGATGCTCCTGAATCGCGGTGGAAAGTCCT<br>CAGAGAGAGAGTGCGAGATTTGCCACTCCGTGGAGAACCTGGTGTCCTACCACGACCAGAAAGTCTGTGAC<br>ATTTGCCGGGGACTGTACCAGTTCTCGAAAGAAATTGCCCATGACCACTTCATCATTACCGAAAATGAGGG<br>GCTGCCGATTGGACCAAACGCGTGCTTAAAGGGCGTGGCATTCGAAAAGCTGTCCCAAGAAGCGTTCAGCC<br>GGGTCTACGTGAAGAATGACTATAAGGCCGGTACCGTGAAGGCTACGCATGTGTTCGTGGGGGATTACCAG<br>TGCGACGAGATCTACAACTACGCCGCCCTGAGCAAGAACGAGAACGGCCTAGGCATCAAGAGACTGGCCGT<br>GGTCCGGCTCGACGTGGATGACTTGGGCGCCGCCTTCATGGCCGGTTTCAGCCAGCAGGGAAACGGACAAT<br>ACTCCACTCTGTCAAGATCGGCCACATTCTCCCGGAGCATGTCGCTGTTCTTCAAAGTGTACATTAACCAG<br>TTCGCCTCCGACAAGAAGCTGAGCATTATCTACGCGGGCGGCGCCGCCGTGTTCGCCATTGGATCGTGGCA<br>GGATATCATCGCGTTCACTGTGGAACTTCGCGAAAACTTCATCAAGTGGACCAACGGGAAGCTCACCCTCT<br>CCGCGGGGATAGGGTTGTTCGCCGACAAGACTCCTATTAGCCTGATGGCTCACCAGACCGGGGAACTGGAA<br>GAGGCCGCCAAGGGCAACGAAAAGGACTCCATCTCGCTGTTCTCAAGCGACTACACTTTCAAGTTTGATAG<br>GTTCATCACTAACGTGTACGACGACAAACTGGAACAGATTAGATACTTCTTCAACCATCAAGACGAGAGGG<br>GAAAGAACTTCATCTATAAGCTTATTGAGCTTTTGAGGAACCACGACCGCATGAATATGGCACGCCTCGCC<br>TATTACCTCACTCGCCTGGAAGAACTGACCCGGGAGACTGACAGGGACAAGTTCAAGACCTTCAAGAACCT<br>GTTCTACTCCTGGTACACCAACAAGAACGATAAGGACCGGAAGGAAGCCGAGCTCGCGCTCCTGCTGTACA<br>TCTACGAAATCAGAAAGGATTAAcggcaataaaaagacagaataaaacgcacggtgttgggtcgtttgttc<br>AAGCTC

**Plasmid**	**pDAC309**
**Description**	Expression of Csm2
**Utility**	
**Features**	Pcmv-FLAG-NLS-Csm2-pA
**Sequence**	ACATGTGAGCAAAAGGCCAGCAAAAGGCCAGGAACCGTAAAAAGGCCGCGTTGCTGGCGTTTTTCCATAGG<br>CTCCGCCCCCCTGACGAGCATCACAAAAATCGACGCTCAAGTCAGAGGTGGCGAAACCCGACAGGACTATA<br>AAGATACCAGGCGTTTCCCCCTGGAAGCTCCCTCGTGCGCTCTCCTGTTCCGACCCTGCCGCTTACCGGAT<br>ACCTGTCCGCCTTTCTCCCTTCGGGAAGCGTGGCGCTTTCTCATAGCTCACGCTGTAGGTATCTCAGTTCG<br>GTGTAGGTCGTTCGCTCCAAGCTGGGCTGTGTGCACGAACCCCCCGTTCAGCCCGACCGCTGCGCCTTATC<br>CGGTAACTATCGTCTTGAGTCCAACCCGGTAAGACACGACTTATCGCCACTGGCAGCAGCCACTGGTAACA<br>GGATTAGCAGAGCGAGGTATGTAGGCGGTGCTACAGAGTTCTTGAAGTGGTGGCCTAACTACGGCTACACT<br>AGAAGAACAGTATTTGGTATCTGCGCTCTGCTGAAGCCAGTTACCTTCGGAAAAAGAGTTGGTAGCTCTTG<br>ATCCGGCAAACAAACCACCGCTGGTAGCGGTGGTTTTTTTGTTTGCAAGCAGCAGATTACGCGCAGAAAAA<br>AAGGATCTCAAGAAGATCCTTTGATCTTTTCTACGGGGTCTGACGCTCAGTGGAACGAAAACTCACGTTAA<br>GGGATTTTGGTCATGAGATTATCAAAAAGGATCTTCACCTAGATCCTTTTAAATTAAAAATGAAGTTTTAA<br>ATCAATCTAAAGTATATATGAGTAAACTTGGTCTGACAGTTACCAATGCTTAATCAGTGAGGCACCTATCT<br>CAGCGATCTGTCTATTTCGTTCATCCATAGTTGCCTGACTCCCCGTCGTGTAGATAACTACGATACGGGAG<br>GGCTTACCATCTGGCCCCAGTGCTGCAATGATACCGCGAGACCCACGCTCACCGGCTCCAGATTTATCAGC<br>AATAAACCAGCCAGCCGGAAGGGCCGAGCGCAGAAGTGGTCCTGCAACTTTATCCGCCTCCATCCAGTCTA<br>TTAATTGTTGCCGGGAAGCTAGAGTAAGTAGTTCGCCAGTTAATAGTTTGCGCAACGTTGTTGCCATTGCT<br>ACAGGCATCGTGGTGTCACGCTCGTCGTTTGGTATGGCTTCATTCAGCTCCGGTTCCCAACGATCAAGGCG<br>AGTTACATGATCCCCCATGTTGTGCAAAAAAGCGGTTAGCTCCTTCGGTCCTCCGATCGTTGTCAGAAGTA<br>AGTTGGCCGCAGTGTTATCACTCATGGTTATGGCAGCACTGCATAATTCTCTTACTGTCATGCCATCCGTA<br>AGATGCTTTTCTGTGACTGGTGAGTACTCAACCAAGTCATTCTGAGAATAGTGTATGCGGCGACCGAGTTG<br>CTCTTGCCCGGCGTCAATACGGGATAATACCGCGCCACATAGCAGAACTTTAAAAGTGCTCATCATTGGAA<br>AACGTTCTTCGGGGCGAAAACTCTCAAGGATCTTACCGCTGTTGAGATCCAGTTCGATGTAACCCACTCGT<br>GCACCCAACTGATCTTCAGCATCTTTTACTTTCACCAGCGTTTCTGGGTGAGCAAAAACAGGAAGGCAAAA

	TGCCGCAAAAAAGGGAATAAGGGCGACACGGAAATGTTGAATACTCATACTCTTCCTTTTTCAATATTATT<br>GAAGCATTTATCAGGGTTATTGTCTCATGAGCGGATACATATTTGAATGTATTTAGAAAAATAAACAAATA<br>GGGGTTCCGCGCACATTTCCCCGAAAAGTGCCACCTGACGTCGGATCCgacattgattattgactagttat<br>taatagtaatcaattacggggtcattagttcatagcccatatatggagttccgCGTTACATAACTTACGGT<br>AAATGGCCCGCCTGGCTGACCGCCCAACGACCCCCGCCCATTGACGTCAATAATGACGTATGTTCCCATAG<br>TAACGCCAATAGGGACTTTCCATTGACGTCAATGGGTGGAGTATTTACGGTAAACTGCCCACTTGGCAGTA<br>CATCAAGTGTATCATATGCCAAGTACGCCCCCTATTGACGTCAATGACGGTAAATGGCCCGCCTGGCATTA<br>TGCCCAGTACATGACCTTATGGGACTTTCCTACTTGGCAGTACATCTACGTATTAGTCATCGCTATTACCA<br>TGGTGATGCGGTTTTGGCAGTACATCAATGGGCGTGGATAGCGGTTTGACTCACGGGGATTTCCAAGTCTC<br>CACCCCATTGACGTCAATGGGAGTTTGTTTTGGCACCAAAATCAACGGGACTTTCCAAAATGTCGTAACAA<br>CTCCGCCCCATTGACGCAAATGGGCGGTAGGCGTGTACGGTGGGAGGTCTATATAAGCAGAGCTCGTTTAG<br>TGAACCGTCAGATCTCTAGAgccgccaccATGGATTACAAAGACGATGACGATAAGATGGCGCCTAAGAAG<br>AAACGCAAAGTGCGGGGCATGACCATCCTGACCGACGAGAACTACGTGGACATCGCCGAGAAAGCCATCCT<br>GAAGCTGGAAAGAAACACCAGAAATAGAAAGAACCCTGATGCCTTCTTCCTGACCACATCTAAGCTGCGGA<br>ACCTGCTGAGCCTGACAAGCACCCTGTTCGACGAGAGCAAGGTGAAGGAATACGACGCCCTGCTGGACAGA<br>ATCGCTTATCTGAGAGTGCAGTTCGTGTACCAGGCCGGCAGAGAGATCGCCGTGAAAGATCTGATCGAGAA<br>GGCCCAGATCCTGGAAGCTCTGAAAGAGATCAAGGACCGGGAAACCCTGCAGAGATTCTGCAGATACATGG<br>AAGCCCTGGTGGCCTACTTCAAGTTCTACGGCGGCAAGGACTGAcggcaataaaagacagaataaaacgc<br>acggtgttgggtcgtttgttcAAGCTC

**Plasmid**	**pDAC310**
**Description**	Expression of Csm3
**Utility**	
**Features**	Pcmv-FLAG-NLS-Csm3-pA
**Sequence**	ACATGTGAGCAAAAGGCCAGCAAAAGGCCAGGAACCGTAAAAAGGCCGCGTTGCTGGCGTTTTTCCATAGG<br>CTCCGCCCCCCTGACGAGCATCACAAAAATCGACGCTCAAGTCAGAGGTGGCGAAACCCGACAGGACTATA<br>AAGATACCAGGCGTTTCCCCCTGGAAGCTCCCTCGTGCGCTCTCCTGTTCCGACCCTGCCGCTTACCGGAT<br>ACCTGTCCGCCTTTCTCCCTTCGGGAAGCGTGGCGCTTTCTCATAGCTCACGCTGTAGGTATCTCAGTTCG<br>GTGTAGGTCGTTCGCTCCAAGCTGGGCTGTGTGCACGAACCCCCCGTTCAGCCCGACCGCTGCGCCTTATC<br>CGGTAACTATCGTCTTGAGTCCAACCCGGTAAGACACGACTTATCGCCACTGGCAGCAGCCACTGGTAACA<br>GGATTAGCAGAGCGAGGTATGTAGGCGGTGCTACAGAGTTCTTGAAGTGGTGGCCTAACTACGGCTACACT<br>AGAAGAACAGTATTTGGTATCTGCGCTCTGCTGAAGCCAGTTACCTTCGGAAAAAGAGTTGGTAGCTCTTG<br>ATCCGGCAAACAAACCACCGCTGGTAGCGGTGGTTTTTTTGTTTGCAAGCAGCAGATTACGCGCAGAAAAA<br>AAGGATCTCAAGAAGATCCTTTGATCTTTTCTACGGGGTCTGACGCTCAGTGGAACGAAAACTCACGTTAA<br>GGGATTTTGGTCATGAGATTATCAAAAAGGATCTTCACCTAGATCCTTTTAAATTAAAAATGAAGTTTTAA<br>ATCAATCTAAAGTATATATGAGTAAACTTGGTCTGACAGTTACCAATGCTTAATCAGTGAGGCACCTATCT<br>CAGCGATCTGTCTATTTCGTTCATCCATAGTTGCCTGACTCCCCGTCGTGTAGATAACTACGATACGGGAG<br>GGCTTACCATCTGGCCCCAGTGCTGCAATGATACCGCGAGACCCACGCTCACCGGCTCCAGATTTATCAGC<br>AATAAACCAGCCAGCCGGAAGGGCCGAGCGCAGAAGTGGTCCTGCAACTTTATCCGCCTCCATCCAGTCTA<br>TTAATTGTTGCCGGGAAGCTAGAGTAAGTAGTTCGCCAGTTAATAGTTTGCGCAACGTTGTTGCCATTGCT<br>ACAGGCATCGTGGTGTCACGCTCGTCGTTTGGTATGGCTTCATTCAGCTCCGGTTCCCAACGATCAAGGCG<br>AGTTACATGATCCCCCATGTTGTGCAAAAAAGCGGTTAGCTCCTTCGGTCCTCCGATCGTTGTCAGAAGTA<br>AGTTGGCCGCAGTGTTATCACTCATGGTTATGGCAGCACTGCATAATTCTCTTACTGTCATGCCATCCGTA<br>AGATGCTTTTCTGTGACTGGTGAGTACTCAACCAAGTCATTCTGAGAATAGTGTATGCGGCGACCGAGTTG<br>CTCTTGCCCGGCGTCAATACGGGATAATACCGCGCCACATAGCAGAACTTTAAAAGTGCTCATCATTGGAA<br>AACGTTCTTCGGGGCGAAAACTCTCAAGGATCTTACCGCTGTTGAGATCCAGTTCGATGTAACCCACTCGT<br>GCACCCAACTGATCTTCAGCATCTTTTACTTTCACCAGCGTTTCTGGGTGAGCAAAAACAGGAAGGCAAAA<br>TGCCGCAAAAAAGGGAATAAGGGCGACACGGAAATGTTGAATACTCATACTCTTCCTTTTTCAATATTATT<br>GAAGCATTTATCAGGGTTATTGTCTCATGAGCGGATACATATTTGAATGTATTTAGAAAAATAAACAAATA<br>GGGGTTCCGCGCACATTTCCCCGAAAAGTGCCACCTGACGTCGGATCCgacattgattattgactagttat<br>taatagtaatcaattacggggtcattagttcatagcccatatatggagttccgCGTTACATAACTTACGGT<br>AAATGGCCCGCCTGGCTGACCGCCCAACGACCCCCGCCCATTGACGTCAATAATGACGTATGTTCCCATAG<br>TAACGCCAATAGGGACTTTCCATTGACGTCAATGGGTGGAGTATTTACGGTAAACTGCCCACTTGGCAGTA<br>CATCAAGTGTATCATATGCCAAGTACGCCCCCTATTGACGTCAATGACGGTAAATGGCCCGCCTGGCATTA<br>TGCCCAGTACATGACCTTATGGGACTTTCCTACTTGGCAGTACATCTACGTATTAGTCATCGCTATTACCA<br>TGGTGATGCGGTTTTGGCAGTACATCAATGGGCGTGGATAGCGGTTTGACTCACGGGGATTTCCAAGTCTC<br>CACCCCATTGACGTCAATGGGAGTTTGTTTTGGCACCAAAATCAACGGGACTTTCCAAAATGTCGTAACAA<br>CTCCGCCCCATTGACGCAAATGGGCGGTAGGCGTGTACGGTGGGAGGTCTATATAAGCAGAGCTCGTTTAG<br>TGAACCGTCAGATCTCTAGAgccgccaccATGGATTACAAAGACGATGACGATAAGATGGCGCCTAAGAAG<br>AAACGCAAAGTGCGGGGCATGACCTTCGCCAAGATCAAATTCAGCGCCCAGATCCGGCTGGAAACCGGCCT<br>GCACATCGGAGGATCTGATGCCTTTGCCGCTATCGGCGCCATCGACAGCCCTGTGATCAAGGACCCCATCA<br>CCAACCTGCCTATCATCCCCGGCTCTAGCCTGAAGGGCAAGATGAGAACACTGCTGGCCAAGGTGTACAAC<br>GAAAAGGTGGCCGAGAAGCCTAGCGACGACAGCGACATCCTGAGCAGACTGTTCGGAAATAGCAAGGATAA

	GCGGTTCAAGATGGGCAGACTGATCTTCCGGGACGCCTTCCTGAGCAACGCCGACGAGCTGGATTCTCTGG<br>GCGTGCGGAGCTACACCGAGGTGAAGTTCGAGAACACCATCGATAGAATCACCGCCGAGGCCAATCCTAGA<br>CAGATCGAGAGAGCCATTCGGAACTCAACATTCGACTTCGAGCTGATCTACGAGATCACTGATGAGAATGA<br>GAACCAGGTCGAGGAAGATTTCAAGGTGATCAGAGACGGCCTGAAGCTGCTGGAACTGGACTACCTGGGCG<br>GAAGCGGCTCCAGAGGCTACGGCAAAGTGGCTTTTGAGAACCTGAAAGCCACCACAGTGTTCGGCAACTAC<br>GACGTGAAAACCCTGAACGAGCTGCTGACCGCCGAAGTGTGAcggcaataaaaagacagaataaaacgcac<br>ggtgttgggtcgtttgttcAAGCTC

**Plasmid**	**pDAC327**
**Description**	Expression of Csm3(RNase mut)
**Utility**	
**Features**	Pcmv-FLAG-NLS-Csm3(RNase mut)-pA
**Sequence**	ACATGTGAGCAAAAGGCCAGCAAAAGGCCAGGAACCGTAAAAAGGCCGCGTTGCTGGCGTTTTTCCATAGG<br>CTCCGCCCCCCTGACGAGCATCACAAAAATCGACGCTCAAGTCAGAGGTGGCGAAACCCGACAGGACTATA<br>AAGATACCAGGCGTTTCCCCCTGGAAGCTCCCTCGTGCGCTCTCCTGTTCCGACCCTGCCGCTTACCGGAT<br>ACCTGTCCGCCTTTCTCCCTTCGGGAAGCGTGGCGCTTTCTCATAGCTCACGCTGTAGGTATCTCAGTTCG<br>GTGTAGGTCGTTCGCTCCAAGCTGGGCTGTGTGCACGAACCCCCCGTTCAGCCCGACCGCTGCGCCTTATC<br>CGGTAACTATCGTCTTGAGTCCAACCCGGTAAGACACGACTTATCGCCACTGGCAGCAGCCACTGGTAACA<br>GGATTAGCAGAGCGAGGTATGTAGGCGGTGCTACAGAGTTCTTGAAGTGGTGGCCTAACTACGGCTACACT<br>AGAAGAACAGTATTTGGTATCTGCGCTCTGCTGAAGCCAGTTACCTTCGGAAAAAGAGTTGGTAGCTCTTG<br>ATCCGGCAAACAAACCACCGCTGGTAGCGGTGGTTTTTTTGTTTGCAAGCAGCAGATTACGCGCAGAAAAA<br>AAGGATCTCAAGAAGATCCTTTGATCTTTTCTACGGGGTCTGACGCTCAGTGGAACGAAAACTCACGTTAA<br>GGGATTTTGGTCATGAGATTATCAAAAAGGATCTTCACCTAGATCCTTTTAAATTAAAAATGAAGTTTTAA<br>ATCAATCTAAAGTATATATGAGTAAACTTGGTCTGACAGTTACCAATGCTTAATCAGTGAGGCACCTATCT<br>CAGCGATCTGTCTATTTCGTTCATCCATAGTTGCCTGACTCCCCGTCGTGTAGATAACTACGATACGGGAG<br>GGCTTACCATCTGGCCCCAGTGCTGCAATGATACCGCGAGACCCACGCTCACCGGCTCCAGATTTATCAGC<br>AATAAACCAGCCAGCCGGAAGGGCCGAGCGCAGAAGTGGTCCTGCAACTTTATCCGCCTCCATCCAGTCTA<br>TTAATTGTTGCCGGGAAGCTAGAGTAAGTAGTTCGCCAGTTAATAGTTTGCGCAACGTTGTTGCCATTGCT<br>ACAGGCATCGTGGTGTCACGCTCGTCGTTTGGTATGGCTTCATTCAGCTCCGGTTCCCAACGATCAAGGCG<br>AGTTACATGATCCCCCATGTTGTGCAAAAAAGCGGTTAGCTCCTTCGGTCCTCCGATCGTTGTCAGAAGTA<br>AGTTGGCCGCAGTGTTATCACTCATGGTTATGGCAGCACTGCATAATTCTCTTACTGTCATGCCATCCGTA<br>AGATGCTTTTCTGTGACTGGTGAGTACTCAACCAAGTCATTCTGAGAATAGTGTATGCGGCGACCGAGTTG<br>CTCTTGCCCGGCGTCAATACGGGATAATACCGCGCCACATAGCAGAACTTTAAAAGTGCTCATCATTGGAA<br>AACGTTCTTCGGGGCGAAAACTCTCAAGGATCTTACCGCTGTTGAGATCCAGTTCGATGTAACCCACTCGT<br>GCACCCAACTGATCTTCAGCATCTTTTACTTTCACCAGCGTTTCTGGGTGAGCAAAAACAGGAAGGCAAAA<br>TGCCGCAAAAAAGGGAATAAGGGCGACACGGAAATGTTGAATACTCATACTCTTCCTTTTTCAATATTATT<br>GAAGCATTTATCAGGGTTATTGTCTCATGAGCGGATACATATTTGAATGTATTTAGAAAAATAAACAAATA<br>GGGGTTCCGCGCACATTTCCCCGAAAAGTGCCACCTGACGTCGGATCCgacattgattattgactagttat<br>taatagtaatcaattacggggtcattagttcatagcccatatatggagttccgCGTTACATAACTTACGGT<br>AAATGGCCCGCCTGGCTGACCGCCCAACGACCCCCGCCCATTGACGTCAATAATGACGTATGTTCCCATAG<br>TAACGCCAATAGGGACTTTCCATTGACGTCAATGGGTGGAGTATTTACGGTAAACTGCCCACTTGGCAGTA<br>CATCAAGTGTATCATATGCCAAGTACGCCCCCTATTGACGTCAATGACGGTAAATGGCCCGCCTGGCATTA<br>TGCCCAGTACATGACCTTATGGGACTTTCCTACTTGGCAGTACATCTACGTATTAGTCATCGCTATTACCA<br>TGGTGATGCGGTTTTGGCAGTACATCAATGGGCGTGGATAGCGGTTTGACTCACGGGGATTTCCAAGTCTC<br>CACCCCATTGACGTCAATGGGAGTTTGTTTTGGCACCAAAATCAACGGGACTTTCCAAAATGTCGTAACAA<br>CTCCGCCCCATTGACGCAAATGGGCGGTAGGCGTGTACGGTGGGAGGTCTATATAAGCAGAGCTCGTTTAG<br>TGAACCGTCAGATCTCTAGAgccgccaccATGGATTACAAAGACGATGACGATAAGATGGCGCCTAAGAAG<br>AAACGCAAAGTGCGGGGCATGACCTTCGCCAAGATCAAATTCAGCGCCCAGATCCGGCTGGAAACCGGCCT<br>GCACATCGGAGGATCTGATGCCTTTGCCGCTATCGGCGCCATCGCCAGCCCTGTGATCAAGGACCCCATCA<br>CCAACCTGCCTATCATCCCCGGCTCTAGCCTGAAGGGCAAGATGAGAACACTGCTGGCCAAGGTGTACAAC<br>GAAAAGGTGGCCGAGAAGCCTAGCGACGACAGCGACATCCTGAGCAGACTGTTCGGAAATAGCAAGGATAA<br>GCGGTTCAAGATGGGCAGACTGATCTTCCGGGACGCCTTCCTGAGCAACGCCGACGAGCTGGATTCTCTGG<br>GCGTGCGGAGCTACACCGAGGTGAAGTTCGAGAACACCATCGATAGAATCACCGCCGAGGCCAATCCTAGA<br>CAGATCGAGAGAGCCATTCGGAACTCAACATTCGACTTCGAGCTGATCTACGAGATCACTGATGAGAATGA<br>GAACCAGGTCGAGGAAGATTTCAAGGTGATCAGAGACGGCCTGAAGCTGCTGGAACTGGACTACCTGGGCG<br>GAAGCGGCTCCAGAGGCTACGGCAAAGTGGCTTTTGAGAACCTGAAAGCCACCACAGTGTTCGGCAACTAC<br>GACGTGAAAACCCTGAACGAGCTGCTGACCGCCGAAGTGTGAcggcaataaaaagacagaataaaacgcac<br>ggtgttgggtcgtttgttcAAGCTC

**Plasmid**	**pDAC339**
**Description**	Expression of Csm4
**Utility**	

**Features**	Pcmv-FLAG-NLS-Csm4-pA
**Sequence**	ACATGTGAGCAAAAGGCCAGCAAAAGGCCAGGAACCGTAAAAAGGCCGCGTTGCTGGCGTTTTTCCATAGG<br>CTCCGCCCCCCTGACGAGCATCACAAAAATCGACGCTCAAGTCAGAGGTGGCGAAACCCGACAGGACTATA<br>AAGATACCAGGCGTTTCCCCCTGGAAGCTCCCTCGTGCGCTCTCCTGTTCCGACCCTGCCGCTTACCGGAT<br>ACCTGTCCGCCTTTCTCCCTTCGGGAAGCGTGGCGCTTTCTCATAGCTCACGCTGTAGGTATCTCAGTTCG<br>GTGTAGGTCGTTCGCTCCAAGCTGGGCTGTGTGCACGAACCCCCCGTTCAGCCCGACCGCTGCGCCTTATC<br>CGGTAACTATCGTCTTGAGTCCAACCCGGTAAGACACGACTTATCGCCACTGGCAGCAGCCACTGGTAACA<br>GGATTAGCAGAGCGAGGTATGTAGGCGGTGCTACAGAGTTCTTGAAGTGGTGGCCTAACTACGGCTACACT<br>AGAAGAACAGTATTTGGTATCTGCGCTCTGCTGAAGCCAGTTACCTTCGGAAAAAGAGTTGGTAGCTCTTG<br>ATCCGGCAAACAAACCACCGCTGGTAGCGGTGGTTTTTTTGTTTGCAAGCAGCAGATTACGCGCAGAAAAA<br>AAGGATCTCAAGAAGATCCTTTGATCTTTTCTACGGGGTCTGACGCTCAGTGGAACGAAAACTCACGTTAA<br>GGGATTTTGGTCATGAGATTATCAAAAAGGATCTTCACCTAGATCCTTTTAAATTAAAAATGAAGTTTTAA<br>ATCAATCTAAAGTATATATGAGTAAACTTGGTCTGACAGTTACCAATGCTTAATCAGTGAGGCACCTATCT<br>CAGCGATCTGTCTATTTCGTTCATCCATAGTTGCCTGACTCCCCGTCGTGTAGATAACTACGATACGGGAG<br>GGCTTACCATCTGGCCCCAGTGCTGCAATGATACCGCGAGACCCACGCTCACCGGCTCCAGATTTATCAGC<br>AATAAACCAGCCAGCCGGAAGGGCCGAGCGCAGAAGTGGTCCTGCAACTTTATCCGCCTCCATCCAGTCTA<br>TTAATTGTTGCCGGGAAGCTAGAGTAAGTAGTTCGCCAGTTAATAGTTTGCGCAACGTTGTTGCCATTGCT<br>ACAGGCATCGTGGTGTCACGCTCGTCGTTTGGTATGGCTTCATTCAGCTCCGGTTCCCAACGATCAAGGCG<br>AGTTACATGATCCCCCATGTTGTGCAAAAAAGCGGTTAGCTCCTTCGGTCCTCCGATCGTTGTCAGAAGTA<br>AGTTGGCCGCAGTGTTATCACTCATGGTTATGGCAGCACTGCATAATTCTCTTACTGTCATGCCATCCGTA<br>AGATGCTTTTCTGTGACTGGTGAGTACTCAACCAAGTCATTCTGAGAATAGTGTATGCGGCGACCGAGTTG<br>CTCTTGCCCGGCGTCAATACGGGATAATACCGCGCCACATAGCAGAACTTTAAAAGTGCTCATCATTGGAA<br>AACGTTCTTCGGGGCGAAAACTCTCAAGGATCTTACCGCTGTTGAGATCCAGTTCGATGTAACCCACTCGT<br>GCACCCAACTGATCTTCAGCATCTTTTACTTTCACCAGCGTTTCTGGGTGAGCAAAAACAGGAAGGCAAAA<br>TGCCGCAAAAAAGGGAATAAGGGCGACACGGAAATGTTGAATACTCATACTCTTCCTTTTTCAATATTATT<br>GAAGCATTTATCAGGGTTATTGTCTCATGAGCGGATACATATTTGAATGTATTTAGAAAAATAAACAAATA<br>GGGGTTCCGCGCACATTTCCCCGAAAAGTGCCACCTGACGTCGGATCCgacattgattattgactagttat<br>taatagtaatcaattacggggtcattagttcatagcccatatatggagttccgCGTTACATAACTTACGGT<br>AAATGGCCCGCCTGGCTGACCGCCCAACGACCCCCGCCCATTGACGTCAATAATGACGTATGTTCCCATAG<br>TAACGCCAATAGGGACTTTCCATTGACGTCAATGGGTGGAGTATTTACGGTAAACTGCCCACTTGGCAGTA<br>CATCAAGTGTATCATATGCCAAGTACGCCCCCTATTGACGTCAATGACGGTAAATGGCCCGCCTGGCATTA<br>TGCCCAGTACATGACCTTATGGGACTTTCCTACTTGGCAGTACATCTACGTATTAGTCATCGCTATTACCA<br>TGGTGATGCGGTTTTGGCAGTACATCAATGGGCGTGGATAGCGGTTTGACTCACGGGGATTTCCAAGTCTC<br>CACCCCATTGACGTCAATGGGAGTTTGTTTTGGCACCAAAATCAACGGGACTTTCCAAAATGTCGTAACAA<br>CTCCGCCCCATTGACGCAAATGGGCGGTAGGCGTGTACGGTGGGAGGTCTATATAAGCAGAGCTCGTTTAG<br>TGAACCGTCAGATCTCTAGAgccgccaccATGGATTACAAAGACGATGACGATAAGATGGCGCCTAAGAAG<br>AAACGCAAAGTGCGGGGCATGACTTACAAGCTCTACATTATGACCTTTCAAAACGCCCACTTCGGTTCCGG<br>CACTCTGGACTCATCGAAGCTGACCTTCTCCGCGGATAGAATCTTCTCGGCACTCGTGCTCGAGGCTCTGA<br>AGATGGGAAAGCTCGACGCCTTCTTGGCCGAGGCCAACCAGGATAAGTTCACTCTGACCGACGCGTTCCCA<br>TTCCAATTCGGTCCTTTCCTGCCGAAACCGATTGGTTACCCCAAGCACGACCAGATCGACCAGTCTGTGGA<br>CGTGAAGGAAGTCCGCCGCCAAGCGAAGCTGTCCAAAAAGCTCCAGTTCCTGGCTCTGGAAAACGTCGACG<br>ACTACCTGAACGGAGAGCTGTTTGAGAATGAGGAACACGCCGTGATCGACACAGTGACCAAGAACCAGCCC<br>CATAAAGATGATAATCTGTACCAAGTGGCCACCACTCGGTTCTCGAACGACACCTCCCTTTACGTGATCGC<br>CAACGAATCCGATCTGCTGAACGAACTGATGAGCAGCCTTCAGTACTCCGGGCTGGGCGGCAAAAGGTCCT<br>CAGGATTCGGCAGATTTGAGCTGGACATCCAGAACATTCCCTTGGAACTGTCCGACCGGCTGACGAAGAAC<br>CACAGCGACAAGGTCATGTCACTTACCACCGCCCTCCCGGTGGACGCTGATCTCGAGGAAGCGATGGAAGA<br>TGGCCATTACCTGTTGACCAAGTCGTCCGGATTCGCATTCTCCCACGCCACCAACGAAAACTATCGGAAGC<br>AGGACCTGTACAAGTTCGCCTCCGGGAGCACCTTCAGCAAGACTTTCGAGGGACAGATCGTGGACGTGCGC<br>CCTCTCGATTTCCCTCACGCCGTGCTGAACTACGCCAAGCCGCTGTTCTTTAAGCTCGAAGTCTAAcggca<br>ataaaaagacagaataaaacgcacggtgttgggtcgtttgttcAAGCTC

**Plasmid**	**pDAC312**
**Description**	Expression of Csm5
**Utility**	
**Features**	Pcmv-FLAG-NLS-Csm5-pA
**Sequence**	ACATGTGAGCAAAAGGCCAGCAAAAGGCCAGGAACCGTAAAAAGGCCGCGTTGCTGGCGTTTTTCCATAGG<br>CTCCGCCCCCCTGACGAGCATCACAAAAATCGACGCTCAAGTCAGAGGTGGCGAAACCCGACAGGACTATA<br>AAGATACCAGGCGTTTCCCCCTGGAAGCTCCCTCGTGCGCTCTCCTGTTCCGACCCTGCCGCTTACCGGAT<br>ACCTGTCCGCCTTTCTCCCTTCGGGAAGCGTGGCGCTTTCTCATAGCTCACGCTGTAGGTATCTCAGTTCG<br>GTGTAGGTCGTTCGCTCCAAGCTGGGCTGTGTGCACGAACCCCCCGTTCAGCCCGACCGCTGCGCCTTATC<br>CGGTAACTATCGTCTTGAGTCCAACCCGGTAAGACACGACTTATCGCCACTGGCAGCAGCCACTGGTAACA<br>GGATTAGCAGAGCGAGGTATGTAGGCGGTGCTACAGAGTTCTTGAAGTGGTGGCCTAACTACGGCTACACT<br>AGAAGAACAGTATTTGGTATCTGCGCTCTGCTGAAGCCAGTTACCTTCGGAAAAAGAGTTGGTAGCTCTTG

	ATCCGGCAAACAAACCACCGCTGGTAGCGGTGGTTTTTTTGTTTGCAAGCAGCAGATTACGCGCAGAAAAA<br>AAGGATCTCAAGAAGATCCTTTGATCTTTTCTACGGGGTCTGACGCTCAGTGGAACGAAAACTCACGTTAA<br>GGGATTTTGGTCATGAGATTATCAAAAAGGATCTTCACCTAGATCCTTTTAAATTAAAAATGAAGTTTTAA<br>ATCAATCTAAAGTATATATGAGTAAACTTGGTCTGACAGTTACCAATGCTTAATCAGTGAGGCACCTATCT<br>CAGCGATCTGTCTATTTCGTTCATCCATAGTTGCCTGACTCCCCGTCGTGTAGATAACTACGATACGGGAG<br>GGCTTACCATCTGGCCCCAGTGCTGCAATGATACCGCGAGACCCACGCTCACCGGCTCCAGATTTATCAGC<br>AATAAACCAGCCAGCCGGAAGGGCCGAGCGCAGAAGTGGTCCTGCAACTTTATCCGCCTCCATCCAGTCTA<br>TTAATTGTTGCCGGGAAGCTAGAGTAAGTAGTTCGCCAGTTAATAGTTTGCGCAACGTTGTTGCCATTGCT<br>ACAGGCATCGTGGTGTCACGCTCGTCGTTTGGTATGGCTTCATTCAGCTCCGGTTCCCAACGATCAAGGCG<br>AGTTACATGATCCCCCATGTTGTGCAAAAAAGCGGTTAGCTCCTTCGGTCCTCCGATCGTTGTCAGAAGTA<br>AGTTGGCCGCAGTGTTATCACTCATGGTTATGGCAGCACTGCATAATTCTCTTACTGTCATGCCATCCGTA<br>AGATGCTTTTCTGTGACTGGTGAGTACTCAACCAAGTCATTCTGAGAATAGTGTATGCGGCGACCGAGTTG<br>CTCTTGCCCGGCGTCAATACGGGATAATACCGCGCCACATAGCAGAACTTTAAAAGTGCTCATCATTGGAA<br>AACGTTCTTCGGGGCGAAAACTCTCAAGGATCTTACCGCTGTTGAGATCCAGTTCGATGTAACCCACTCGT<br>GCACCCAACTGATCTTCAGCATCTTTTACTTTCACCAGCGTTTCTGGGTGAGCAAAAACAGGAAGGCAAAA<br>TGCCGCAAAAAAGGGAATAAGGGCGACACGGAAATGTTGAATACTCATACTCTTCCTTTTTCAATATTATT<br>GAAGCATTTATCAGGGTTATTGTCTCATGAGCGGATACATATTTGAATGTATTTAGAAAAATAAACAAATA<br>GGGGTTCCGCGCACATTTCCCCGAAAAGTGCCACCTGACGTCGGATCCgacattgattattgactagttat<br>taatagtaatcaattacggggtcattagttcatagcccatatatggagttccgCGTTACATAACTTACGGT<br>AAATGGCCCGCCTGGCTGACCGCCCAACGACCCCCGCCCATTGACGTCAATAATGACGTATGTTCCCATAG<br>TAACGCCAATAGGGACTTTCCATTGACGTCAATGGGTGGAGTATTTACGGTAAACTGCCCACTTGGCAGTA<br>CATCAAGTGTATCATATGCCAAGTACGCCCCCTATTGACGTCAATGACGGTAAATGGCCCGCCTGGCATTA<br>TGCCCAGTACATGACCTTATGGGACTTTCCTACTTGGCAGTACATCTACGTATTAGTCATCGCTATTACCA<br>TGGTGATGCGGTTTTGGCAGTACATCAATGGGCGTGGATAGCGGTTTGACTCACGGGGATTTCCAAGTCTC<br>CACCCCATTGACGTCAATGGGAGTTTGTTTTGGCACCAAAATCAACGGGACTTTCCAAAATGTCGTAACAA<br>CTCCGCCCCATTGACGCAAATGGGCGGTAGGCGTGTACGGTGGGAGGTCTATATAAGCAGAGCTCGTTTAG<br>TGAACCGTCAGATCTCTAGAgccgccaccATGGATTACAAAGACGATGACGATAAGATGGCGCCTAAGAAG<br>AAACGCAAAGTGCGGGGCATGAAAAATGACTACCGGACCTTCAAGCTGAGCCTGCTGACCCTGGCTCCTAT<br>CCACATCGGCAACGGCGAGAAGTACACCAGCAGAGAATTCATCTACGAGAACAAGAAGTTCTACTTCCCCG<br>ACATGGGCAAGTTCTACAACAAGATGGTGGAAAAGAGACTGGCCGAGAAGTTCGAGGCCTTCCTGATCCAG<br>ACCAGACCCAACGCCAGAAACAACCGGCTGATTTCTTTTCTGAACGACAACAGAATCGCCGAAAGATCTTT<br>TGGCGGCTACAGCATCAGTGAAACCGGCCTGGAATCTGATAAGAACCCTAACAGCGCCGGAGCTATCAACG<br>AGGTGAACAAATTCATCCGGGACGCCTTCGGAAATCCTTACATCCCAGGCAGCAGCCTGAAGGGCGCCATC<br>CGCACCATCCTGATGAACACCACACCTAAGTGGAACAACGAGAACGCCGTGAACGACTTCGGCAGATTCCC<br>AAAGGAAAACAAGAACCTGATCCCTTGGGGACCTAAGAAAGGCAAGGAATACGACGACCTGTTCAACGCCA<br>TCAGAGTGTCCGACAGCAAGCCCTTCGACAACAAAAGCCTGATCCTCGTGCAGAAGTGGGACTACAGCGCC<br>AAAACCAACAAGGCCAAGCCTCTGCCTCTGTACAGAGAGTCTATCAGCCCTCTGACCAAGATCGAGTTCGA<br>GATAACAACAACCACTGATGAGGCCGGCAGACTGATCGAGGAACTGGGAAAGCGGGCCCAGGCCTTTTATA<br>AGGACTACAAGGCCTTTTTCCTGTCTGAATTCCCTGATGATAAGATCCAGGCTAATCTGCAATACCCCATC<br>TACCTGGGCGCCGGCAGCGGCGCTTGGACAAAGACCCTGTTTAAGCAGGCCGACGGCATCCTGCAGCGGAG<br>ATACTCCAGAATGAAAACCAAGATGGTCAAGAAGGGCGTGCTGAAGCTGACAAAGGCCCCTCTGAAAACAG<br>TGAAGATCCCCAGCGGCAACCACAGCCTGGTGAAGAATCACGAGAGCTTCTACGAGATGGGCAAAGCCAAC<br>TTCATGATCAAGGAAATCGACAAGTGAcggcaataaaaagacagaataaaacgcacggtgttgggtcgttt<br>gttcAAGCTC

**Plasmid**	**pDAC307**
**Description**	Expression of Cas6
**Utility**	
**Features**	Pcmv-FLAG-NLS-Cas6-pA
**Sequence**	ACATGTGAGCAAAAGGCCAGCAAAAGGCCAGGAACCGTAAAAAGGCCGCGTTGCTGGCGTTTTTCCATAGG<br>CTCCGCCCCCCTGACGAGCATCACAAAAATCGACGCTCAAGTCAGAGGTGGCGAAACCCGACAGGACTATA<br>AAGATACCAGGCGTTTCCCCCTGGAAGCTCCCTCGTGCGCTCTCCTGTTCCGACCCTGCCGCTTACCGGAT<br>ACCTGTCCGCCTTTCTCCCTTCGGGAAGCGTGGCGCTTTCTCATAGCTCACGCTGTAGGTATCTCAGTTCG<br>GTGTAGGTCGTTCGCTCCAAGCTGGGCTGTGTGCACGAACCCCCCGTTCAGCCCGACCGCTGCGCCTTATC<br>CGGTAACTATCGTCTTGAGTCCAACCCGGTAAGACACGACTTATCGCCACTGGCAGCAGCCACTGGTAACA<br>GGATTAGCAGAGCGAGGTATGTAGGCGGTGCTACAGAGTTCTTGAAGTGGTGGCCTAACTACGGCTACACT<br>AGAAGAACAGTATTTGGTATCTGCGCTCTGCTGAAGCCAGTTACCTTCGGAAAAAGAGTTGGTAGCTCTTG<br>ATCCGGCAAACAAACCACCGCTGGTAGCGGTGGTTTTTTTGTTTGCAAGCAGCAGATTACGCGCAGAAAAA<br>AAGGATCTCAAGAAGATCCTTTGATCTTTTCTACGGGGTCTGACGCTCAGTGGAACGAAAACTCACGTTAA<br>GGGATTTTGGTCATGAGATTATCAAAAAGGATCTTCACCTAGATCCTTTTAAATTAAAAATGAAGTTTTAA<br>ATCAATCTAAAGTATATATGAGTAAACTTGGTCTGACAGTTACCAATGCTTAATCAGTGAGGCACCTATCT<br>CAGCGATCTGTCTATTTCGTTCATCCATAGTTGCCTGACTCCCCGTCGTGTAGATAACTACGATACGGGAG<br>GGCTTACCATCTGGCCCCAGTGCTGCAATGATACCGCGAGACCCACGCTCACCGGCTCCAGATTTATCAGC

	AATAAACCAGCCAGCCGGAAGGGCCGAGCGCAGAAGTGGTCCTGCAACTTTATCCGCCTCCATCCAGTCTA<br>TTAATTGTTGCCGGGAAGCTAGAGTAAGTAGTTCGCCAGTTAATAGTTTGCGCAACGTTGTTGCCATTGCT<br>ACAGGCATCGTGGTGTCACGCTCGTCGTTTGGTATGGCTTCATTCAGCTCCGGTTCCCAACGATCAAGGCG<br>AGTTACATGATCCCCCATGTTGTGCAAAAAAGCGGTTAGCTCCTTCGGTCCTCCGATCGTTGTCAGAAGTA<br>AGTTGGCCGCAGTGTTATCACTCATGGTTATGGCAGCACTGCATAATTCTCTTACTGTCATGCCATCCGTA<br>AGATGCTTTTCTGTGACTGGTGAGTACTCAACCAAGTCATTCTGAGAATAGTGTATGCGGCGACCGAGTTG<br>CTCTTGCCCGGCGTCAATACGGGATAATACCGCGCCACATAGCAGAACTTTAAAAGTGCTCATCATTGGAA<br>AACGTTCTTCGGGGCGAAAACTCTCAAGGATCTTACCGCTGTTGAGATCCAGTTCGATGTAACCCACTCGT<br>GCACCCAACTGATCTTCAGCATCTTTTACTTTCACCAGCGTTTCTGGGTGAGCAAAAACAGGAAGGCAAAA<br>TGCCGCAAAAAAGGGAATAAGGGCGACACGGAAATGTTGAATACTCATACTCTTCCTTTTTCAATATTATT<br>GAAGCATTTATCAGGGTTATTGTCTCATGAGCGGATACATATTTGAATGTATTTAGAAAAATAAACAAATA<br>GGGGTTCCGCGCACATTTCCCCGAAAAGTGCCACCTGACGTCGGATCCgacattgattattgactagttat<br>taatagtaatcaattacggggtcattagttcatagcccatatatggagttccgCGTTACATAACTTACGGT<br>AAATGGCCCGCCTGGCTGACCGCCCAACGACCCCCGCCCATTGACGTCAATAATGACGTATGTTCCCATAG<br>TAACGCCAATAGGGACTTTCCATTGACGTCAATGGGTGGAGTATTTACGGTAAACTGCCCACTTGGCAGTA<br>CATCAAGTGTATCATATGCCAAGTACGCCCCCTATTGACGTCAATGACGGTAAATGGCCCGCCTGGCATTA<br>TGCCCAGTACATGACCTTATGGGACTTTCCTACTTGGCAGTACATCTACGTATTAGTCATCGCTATTACCA<br>TGGTGATGCGGTTTTGGCAGTACATCAATGGGCGTGGATAGCGGTTTGACTCACGGGGATTTCCAAGTCTC<br>CACCCCATTGACGTCAATGGGAGTTTGTTTTGGCACCAAAATCAACGGGACTTTCCAAAATGTCGTAACAA<br>CTCCGCCCCATTGACGCAAATGGGCGGTAGGCGTGTACGGTGGGAGGTCTATATAAGCAGAGCTCGTTTAG<br>TGAACCGTCAGATCTCTAGAgccgccaccATGGATTACAAAGACGATGACGATAAGATGGCGCCTAAGAAG<br>AAACGCAAAGTGCGGGGCATGAAAAAGCTCGTGTTCACCTTTAAGCGGATCGACCACCCTGCTCAGGACCT<br>GGCCGTGAAATTCCACGGCTTCCTGATGGAACAGCTGGATAGCGACTACGTGGACTACCTGCACCAGCAGC<br>AGACCAACCCCTACGCCACAAAGGTGATCCAGGGCAAAGAGAACACCCAGTGGGTCGTGCATCTGCTGACA<br>GACGACATCGAGGACAAGGTGTTCATGACCCTGCTGCAGATCAAGGAAGTGTCCCTGAACGACCTGCCTAA<br>GTTGTCTGTGGAAAAGGTGGAAATCCAGGAGCTGGGCGCTGATAAGCTGCTCGAGATCTTCAACAGCGAGG<br>AAAACCAGACCTACTTCAGCATCATCTTCGAGACACCTACAGGCTTTAAAAGCCAGGGCAGCTACGTGATC<br>TTCCCCAGCATGCGGCTGATCTTTCAGAGCCTGATGCAGAAGTACGGCAGACTGGTGGAAAACCAGCCTGA<br>GATCGAGGAAGATACCCTGGACTACCTGAGCGAGCACAGCACCATCACCAATTACAGACTGGAAACAAGCT<br>ACTTCAGAGTGCATAGACAGAGAATCCCCGCCTTCCGGGGCAAGCTGACCTTCAAGGTGCAGGGAGCCCAG<br>ACACTGAAGGCCTACGTGAAGATGCTGCTGACCTTCGGCGAGTACAGCGGCCTGGGCATGAAAACCAGCCT<br>GGGAATGGGCGGCATCAAGCTGGAAGAAAGAAAGGACTGAcggcaataaaagacagaataaaacgcacgg<br>tgttgggtcgtttgttcAAGCTC

**Plasmid**	**pDAC324**
**Description**	Expression of crRNA
**Utility**	
**Features**	Pu6-crRNA-pT
**Sequence**	TCGCGCGTTTCGGTGATGACGGTGAAAACCTCTGACACATGCAGCTCCCGGAGACGGTCACAGCTTGTCTG<br>TAAGCGGATGCCGGGAGCAGACAAGCCCGTCAGGGCGCGTCAGCGGGTGTTGGCGGGTGTCGGGGCTGGCT<br>TAACTATGCGGCATCAGAGCAGATTGTACTGAGAGTGCACCATATGGAGGGCCTATTTCCCATGATTCCTT<br>CATATTTGCATATACGATACAAGGCTGTTAGAGAGATAATTAGAATTAATTTGACTGTAAACACAAAGATA<br>TTAGTACAAAATACGTGACGTAGAAAGTAATAATTTCTTGGGTAGTTTGCAGTTTTAAAATTATGTTTTAA<br>AATGGACTATCATATGCTTACCGTAACTTGAAAGTATTTCGATTTCTTGGCTTTATATATCTTGTGGAAAG<br>GACGAAACACCGATATAAACCTAATTACCTCGAGAGGGGACGGAAACCCGTCTTCGATGAAGCGATTCAGA<br>AGACTTGATATAAACCTAATTACCTCGAGAGGGGACTTTTTTACATGTGAGCAAAAGGCCAGCAAAAGGCC<br>AGGAACCGTAAAAAGGCCGCGTTGCTGGCGTTTTTCCATAGGCTCCGCCCCCCTGACGAGCATCACAAAAA<br>TCGACGCTCAAGTCAGAGGTGGCGAAACCCGACAGGACTATAAAGATACCAGGCGTTTCCCCCTGGAAGCT<br>CCCTCGTGCGCTCTCCTGTTCCGACCCTGCCGCTTACCGGATACCTGTCCGCCTTTCTCCCTTCGGGAAGC<br>GTGGCGCTTTCTCATAGCTCACGCTGTAGGTATCTCAGTTCGGTGTAGGTCGTTCGCTCCAAGCTGGGCTG<br>TGTGCACGAACCCCCCGTTCAGCCCGACCGCTGCGCCTTATCCGGTAACTATCGTCTTGAGTCCAACCCGG<br>TAAGACACGACTTATCGCCACTGGCAGCAGCCACTGGTAACAGGATTAGCAGAGCGAGGTATGTAGGCGGT<br>GCTACAGAGTTCTTGAAGTGGTGGCCTAACTACGGCTACACTAGAAGAACAGTATTTGGTATCTGCGCTCT<br>GCTGAAGCCAGTTACCTTCGGAAAAAGAGTTGGTAGCTCTTGATCCGGCAAACAAACCACCGCTGGTAGCG<br>GTGGTTTTTTTGTTTGCAAGCAGCAGATTACGCGCAGAAAAAAAGGATCTCAAGAAGATCCTTTGATCTTT<br>TCTACGGGGTCTGACGCTCAGTGGAACGAAAACTCACGTTAAGGGATTTTGGTCATGAGATTATCAAAAAG<br>GATCTTCACCTAGATCCTTTTAAATTAAAAATGAAGTTTTAAATCAATCTAAAGTATATATGAGTAAACTT<br>GGTCTGACAGTTACCAATGCTTAATCAGTGAGGCACCTATCTCAGCGATCTGTCTATTTCGTTCATCCATA<br>GTTGCCTGACTCCCCGTCGTGTAGATAACTACGATACGGGAGGGCTTACCATCTGGCCCCAGTGCTGCAAT<br>GATACCGCGAGACCCACGCTCACCGGCTCCAGATTTATCAGCAATAAACCAGCCAGCCGGAAGGGCCGAGC<br>GCAGAAGTGGTCCTGCAACTTTATCCGCCTCCATCCAGTCTATTAATTGTTGCCGGGAAGCTAGAGTAAGT<br>AGTTCGCCAGTTAATAGTTTGCGCAACGTTGTTGCCATTGCTACAGGCATCGTGGTGTCACGCTCGTCGTT<br>TGGTATGGCTTCATTCAGCTCCGGTTCCCAACGATCAAGGCGAGTTACATGATCCCCCATGTTGTGCAAAA

	AAGCGGTTAGCTCCTTCGGTCCTCCGATCGTTGTCAGAAGTAAGTTGGCCGCAGTGTTATCACTCATGGTT<br>ATGGCAGCACTGCATAATTCTCTTACTGTCATGCCATCCGTAAGATGCTTTTCTGTGACTGGTGAGTACTC<br>AACCAAGTCATTCTGAGAATAGTGTATGCGGCGACCGAGTTGCTCTTGCCCGGCGTCAATACGGGATAATA<br>CCGCGCCACATAGCAGAACTTTAAAAGTGCTCATCATTGGAAAACGTTCTTCGGGGCGAAAACTCTCAAGG<br>ATCTTACCGCTGTTGAGATCCAGTTCGATGTAACCCACTCGTGCACCCAACTGATCTTCAGCATCTTTTAC<br>TTTCACCAGCGTTTCTGGGTGAGCAAAAACAGGAAGGCAAAATGCCGCAAAAAAGGGAATAAGGGCGACAC<br>GGAAATGTTGAATACTCATACTCTTCCTTTTTCAATATTATTGAAGCATTTATCAGGGTTATTGTCTCATG<br>AGCGGATACATATTTGAATGTATTTAGAAAAATAAACAAATAGGGGTTCCGCGCACATTTCCCCGAAAAGT<br>GCCACCTGACGTCTAAGAAACCATTATTATCATGACATTAACCTATAAAAATAGGCGTATCACGAGGCCCT<br>TTCGTC

**Plasmid**	**pDAC439**
**Description**	Expression of Csm complex from single promoter; RFP backbone
**Utility**	RNA KD
**Features**	Pcmv-FLAG-NLS-Csm5-2A-FLAG-NLS-Csm4-2A-FLAG-NLS-Csm3-2A-FLAG-NLS-Csm2-pA; Pcmv-RFP-2A-FLAG-NLS-Cas6-2A-FLAG-NLS-Csm1-pA; Pu6-crRNA-pT
**Sequence**	CTCGAGTAGTTATTAATAGTAATCAATTACGGGGTCATTAGTTCATAGCCCATATATGGAGTTCCGCGTTA<br>CATAACTTACGGTAAATGGCCCGCCTGGCTGACCGCCCAACGACCCCCGCCCATTGACGTCAATAATGACG<br>TATGTTCCCATAGTAACGCCAATAGGGACTTTCCATTGACGTCAATGGGTGGAGTATTTACGGTAAACTGC<br>CCACTTGGCAGTACATCAAGTGTATCATATGCCAAGTACGCCCCCTATTGACGTCAATGACGGTAAATGGC<br>CCGCCTGGCATTATGCCCAGTACATGACCTTATGGGACTTTCCTACTTGGCAGTACATCTACGTATTAGTC<br>ATCGCTATTACCATGGTGATGCGGTTTTGGCAGTACATCAATGGGCGTGGATAGCGGTTTGACTCACGGGG<br>ATTTCCAAGTCTCCACCCCATTGACGTCAATGGGAGTTTGTTTTGGCACCAAAATCAACGGGACTTTCCAA<br>AATGTCGTAACAACTCCGCCCCATTGACGCAAATGGGCGGTAGGCGTGTACGGTGGGAGGTCTATATAAGC<br>AGAGCTGGTTTAGTGAACCGTCAGATCCGCTAGGGATCCgccgccaccATGGATTACAAAGACGATGACGA<br>TAAGATGGCGCCTAAGAAGAAACGCAAAGTGCGGGGCATGAAAAATGACTACCGGACCTTCAAGCTGAGCC<br>TGCTGACCCTGGCTCCTATCCACATCGGCAACGGCGAGAAGTACACCAGCAGAGAATTCATCTACGAGAAC<br>AAGAAGTTCTACTTCCCCGACATGGGCAAGTTCTACAACAAGATGGTGGAAAAGAGACTGGCCGAGAAGTT<br>CGAGGCCTTCCTGATCCAGACCAGACCCAACGCCAGAAACAACCGGCTGATTTCTTTTCTGAACGACAACA<br>GAATCGCCGAAAGATCTTTTGGCGGCTACAGCATCAGTGAAACCGGCCTGGAATCTGATAAGAACCCTAAC<br>AGCGCCGGAGCTATCAACGAGGTGAACAAATTCATCCGGGACGCCTTCGGAAATCCTTACATCCCAGGCAG<br>CAGCCTGAAGGGCGCCATCCGCACCATCCTGATGAACACCACACCTAAGTGGAACAACGAGAACGCCGTGA<br>ACGACTTCGGCAGATTCCCAAAGGAAAACAAGAACCTGATCCCTTGGGGACCTAAGAAAGGCAAGGAATAC<br>GACGACCTGTTCAACGCCATCAGAGTGTCCGACAGCAAGCCCTTCGACAACAAAAGCCTGATCCTCGTGCA<br>GAAGTGGGACTACAGCGCCAAAACCAACAAGGCCAAGCCTCTGCCTCTGTACAGAGAGTCTATCAGCCCTC<br>TGACCAAGATCGAGTTCGAGATAACAACAACCACTGATGAGGCCGGCAGACTGATCGAGGAACTGGGAAAG<br>CGGGCCCAGGCCTTTTATAAGGACTACAAGGCCTTTTTCCTGTCTGAATTCCCTGATGATAAGATCCAGGC<br>TAATCTGCAATACCCCATCTACCTGGGCGCCGGCAGCGGCGCTTGGACAAAGACCCTGTTTAAGCAGGCCG<br>ACGGCATCCTGCAGCGGAGATACTCCAGAATGAAAACCAAGATGGTCAAGAAGGGCGTGCTGAAGCTGACA<br>AAGGCCCCTCTGAAAACAGTGAAGATCCCCAGCGGCAACCACAGCCTGGTGAAGAATCACGAGAGCTTCTA<br>CGAGATGGGCAAAGCCAACTTCATGATCAAGGAAATCGACAAGgaaggaaggggctccctcctcacttgtg<br>gagatgtcgaagaaaatcctggacctGATTACAAAGACGATGACGATAAGATGGCGCCTAAGAAGAAACGC<br>AAAGTGCGGGGCATGACTTACAAGCTCTACATTATGACCTTTCAAAACGCCCACTTCGGTTCCGGCACTCT<br>GGACTCATCGAAGCTGACCTTCTCCGCGGATAGAATCTTCTCGGCACTCGTGCTCGAGGCTCTGAAGATGG<br>GAAAGCTCGACGCCTTCTTGGCCGAGGCCAACCAGGATAAGTTCACTCTGACCGACGCGTTCCCATTCCAA<br>TTCGGTCCTTTCCTGCCGAAACCGATTGGTTACCCCAAGCACGACCAGATCGACCAGTCTGTGGACGTGAA<br>GGAAGTCCGCCGCCAAGCGAAGCTGTCCAAAAAGCTCCAGTTCCTGGCTCTGGAAAACGTCGACGACTACC<br>TGAACGGAGAGCTGTTTGAGAATGAGGAACACGCCGTGATCGACACAGTGACCAAGAACCAGCCCCATAAA<br>GATGATAATCTGTACCAAGTGGCCACCACTCGGTTCTCGAACGACACCTCCCTTTACGTGATCGCCAACGA<br>ATCCGATCTGCTGAACGAACTGATGAGCAGCCTTCAGTACTCCGGGCTGGGCGGCAAAAGGTCCTCAGGAT<br>TCGGCAGATTTGAGCTGGACATCCAGAACATTCCCTTGGAACTGTCCGACCGGCTGACGAAGAACCACAGC<br>GACAAGGTCATGTCACTTACCACCGCCCTCCCGGTGGACGCTGATCTCGAGGAAGCGATGGAAGATGGCCA<br>TTACCTGTTGACCAAGTCGTCCGGATTCGCATTCTCCCACGCCACCAACGAAAACTATCGGAAGCAGGACC<br>TGTACAAGTTCGCCTCCGGGAGCACCTTCAGCAAGACTTTCGAGGGACAGATCGTGGACGTGCGCCCTCTC<br>GATTTCCCTCACGCCGTGCTGAACTACGCCAAGCCGCTGTTCTTTAAGCTCGAAGTCgagggcagaggaag<br>tctgctaacatgcggtgacgtcgaggagaatcctggacctGATTACAAAGACGATGACGATAAGATGGCGC<br>CTAAGAAGAAACGCAAAGTGCGGGGCATGACCTTCGCCAAGATCAAATTCAGCGCCCAGATCCGGCTGGAA<br>ACCGGCCTGCACATCGGAGGATCTGATGCCTTTGCCGCTATCGGCGCCATCGACAGCCCTGTGATCAAGGA<br>CCCCATCACCAACCTGCCTATCATCCCCGGCTCTAGCCTGAAGGGCAAGATGAGAACACTGCTGGCCAAGG<br>TGTACAACGAAAAGGTGGCCGAGAAGCCTAGCGACGACAGCGACATCCTGAGCAGACTGTTCGGAAATAGC<br>AAGGATAAGCGGTTCAAGATGGGCAGACTGATCTTCCGGGACGCCTTCCTGAGCAACGCCGACGAGCTGGA<br>TTCTCTGGGCGTGCGGAGCTACACCGAGGTGAAGTTCGAGAACACCATCGATAGAATCACCGCCGAGGCCA<br>ATCCTAGACAGATCGAGAGAGCCATTCGGAACTCAACATTCGACTTCGAGCTGATCTACGAGATCACTGAT

	GAGAATGAGAACCAGGTCGAGGAAGATTTCAAGGTGATCAGAGACGGCCTGAAGCTGCTGGAACTGGACTA<br>CCTGGGCGGAAGCGGCTCCAGAGGCTACGGCAAAGTGGCTTTTGAGAACCTGAAAGCCACCACAGTGTTCG<br>GCAACTACGACGTGAAAACCCTGAACGAGCTGCTGACCGCCGAAGTGgaggggcgggggtctttgttgact<br>tgcggggatgttgaggagaacccagggccaGATTACAAAGACGATGACGATAAGATGGCGCCTAAGAAGAA<br>ACGCAAAGTGCGGGGCATGACCATCCTGACCGACGAGAACTACGTGGACATCGCCGAGAAAGCCATCCTGA<br>AGCTGGAAAGAAACACCAGAAATAGAAAGAACCCTGATGCCTTCTTCCTGACCACATCTAAGCTGCGGAAC<br>CTGCTGAGCCTGACAAGCACCCTGTTCGACGAGAGCAAGGTGAAGGAATACGACGCCCTGCTGGACAGAAT<br>CGCTTATCTGAGAGTGCAGTTCGTGTACCAGGCCGGCAGAGAGATCGCCGTGAAAGATCTGATCGAGAAGG<br>CCCAGATCCTGGAAGCTCTGAAAGAGATCAAGGACCGGGAAACCCTGCAGAGATTCTGCAGATACATGGAA<br>GCCCTGGTGGCCTACTTCAAGTTCTACGGCGGCAAGGACTGAGCTAGCGCGGCCGCATCGATAAGCTTGTC<br>GACGATATCTCCAGAGGATCATAATCAGCCATACCACATTTGTAGAGGTTTTACTTGCTTTAAAAAACCTC<br>CCACACCTCCCCCTGAACCTGAAACATAAAATGAATGCAATTGTTGTTGTTAACTTGTTTATTGCAGCTTA<br>TAATGGTTACAAATAAAGCAATAGCATCACAAATTTCACAAATAAAGCATTTTTTTCACTGCCCCGAGCTT<br>CCTCGCTCACTGACTCGCTGCGCTCGGTCGTTCGGCTGCGGCGAGCGGTATCAGCTCACTCAAAGGCGGTA<br>ATACGGTTATCCACAGAATCAGGGGATAACGCAGGAAAGAAACTAGTGAGGGCCTATTTCCCATGATTCCT<br>TCATATTTGCATATACGATACAAGGCTGTTAGAGAGATAATTAGAATTAATTTGACTGTAAACACAAAGAT<br>ATTAGTACAAAATACGTGACGTAGAAAGTAATAATTTCTTGGGTAGTTTGCAGTTTTAAAATTATGTTTTA<br>AAATGGACTATCATATGCTTACCGTAACTTGAAAGTATTTCGATTTCTTGGCTTTATATATCTTGTGGAAA<br>GGACGAAACACCGATATAAACCTAATTACCTCGAGAGGGGACGGAAACCCGTCTTCGATGAAGCGATTCAG<br>AAGACTTGATATAAACCTAATTACCTCGAGAGGGGACTTTTTTACATGTGAGCAAAAGGCCAGCAAAAGGC<br>CAGGAACCGTAAAAAGGCCGCGTTGCTGGCGTTTTTCCATAGGCTCCGCCCCCCTGACGAGCATCACAAAA<br>ATCGACGCTCAAGTCAGAGGTGGCGAAACCCGACAGGACTATAAAGATACCAGGCGTTTCCCCCTGGAAGC<br>TCCCTCGTGCGCTCTCCTGTTCCGACCCTGCCGCTTACCGGATACCTGTCCGCCTTTCTCCCTTCGGGAAG<br>CGTGGCGCTTTCTCATAGCTCACGCTGTAGGTATCTCAGTTCGGTGTAGGTCGTTCGCTCCAAGCTGGGCT<br>GTGTGCACGAACCCCCCGTTCAGCCCGACCGCTGCGCCTTATCCGGTAACTATCGTCTTGAGTCCAACCCG<br>GTAAGACACGACTTATCGCCACTGGCAGCAGCCACTGGTAACAGGATTAGCAGAGCGAGGTATGTAGGCGG<br>TGCTACAGAGTTCTTGAAGTGGTGGCCTAACTACGGCTACACTAGAAGAACAGTATTTGGTATCTGCGCTC<br>TGCTGAAGCCAGTTACCTTCGGAAAAAGAGTTGGTAGCTCTTGATCCGGCAAACAAACCACCGCTGGTAGC<br>GGTGGTTTTTTTGTTTGCAAGCAGCAGATTACGCGCAGAAAAAAAGGATCTCAAGAAGATCCTTTGATCTT<br>TTCTACGGGGTCTGACGCTCAGTGGAACGAAAACTCACGTTAAGGGATTTTGGTCATGAGATTATCAAAAA<br>GGATCTTCACCTAGATCCTTTTAAATTAAAAATGAAGTTTTAAATCAATCTAAAGTATATATGAGTAAACT<br>TGGTCTGACAGTTACCAATGCTTAATCAGTGAGGCACCTATCTCAGCGATCTGTCTATTTCGTTCATCCAT<br>AGTTGCCTGACTCCCCGTCGTGTAGATAACTACGATACGGGAGGGCTTACCATCTGGCCCCAGTGCTGCAA<br>TGATACCGCGAGACCCACGCTCACCGGCTCCAGATTTATCAGCAATAAACCAGCCAGCCGGAAGGGCCGAG<br>CGCAGAAGTGGTCCTGCAACTTTATCCGCCTCCATCCAGTCTATTAATTGTTGCCGGGAAGCTAGAGTAAG<br>TAGTTCGCCAGTTAATAGTTTGCGCAACGTTGTTGCCATTGCTACAGGCATCGTGGTGTCACGCTCGTCGT<br>TTGGTATGGCTTCATTCAGCTCCGGTTCCCAACGATCAAGGCGAGTTACATGATCCCCCATGTTGTGCAAA<br>AAAGCGGTTAGCTCCTTCGGTCCTCCGATCGTTGTCAGAAGTAAGTTGGCCGCAGTGTTATCACTCATGGT<br>TATGGCAGCACTGCATAATTCTCTTACTGTCATGCCATCCGTAAGATGCTTTTCTGTGACTGGTGAGTACT<br>CAACCAAGTCATTCTGAGAATAGTGTATGCGGCGACCGAGTTGCTCTTGCCCGGCGTCAACACGGGATAAT<br>ACCGCGCCACATAGCAGAACTTTAAAAGTGCTCATCATTGGAAAACGTTCTTCGGGGCGAAAACTCTCAAG<br>GATCTTACCGCTGTTGAGATCCAGTTCGATGTAACCCACTCGTGCACCCAACTGATCTTCAGCATCTTTTA<br>CTTTCACCAGCGTTTCTGGGTGAGCAAAAACAGGAAGGCAAAATGCCGCAAAAAAGGGAATAAGGGCGACA<br>CGGAAATGTTGAATACTCATACTCTTCCTTTTTCAATATTATTGAAGCATTTATCAGGGTTATTGTCTCAT<br>GAGCGGATACATATTTGAATGTATTTAGAAAAATAAACAAATAGGGGTTCCGCGCACATTTCCCCGAAAAG<br>TGCCACCTGACGTCGGCAGTGAAAAAAATGCTTTATTTGTGAAATTTGTGATGCTATTGCTTTATTTGTAA<br>CCATTATAAGCTGCAATAAACAAGTTAACAACAACAATTGCATTCATTTTATGTTTCAGGTTCAGGGGGAG<br>GTGTGGGAGGTTTTTTAAAGCAAGTAAAACCTCTACAAATGTGGTATGGCTGATTATGATCCTCTAGATTA<br>ATCCTTTCTGATTTCGTAGATGTACAGCAGGAGCGCGAGCTCGGCTTCCTTCCGGTCCTTATCGTTCTTGT<br>TGGTGTACCAGGAGTAGAACAGGTTCTTGAAGGTCTTGAACTTGTCCCTGTCAGTCTCCCGGGTCAGTTCT<br>TCCAGGCGAGTGAGGTAATAGGCGAGGCGTGCCATATTCATGCGGTCGTGGTTCCTCAAAAGCTCAATAAG<br>CTTATAGATGAAGTTCTTTCCCCTCTCGTCTTGATGGTTGAAGAAGTATCTAATCTGTTCCAGTTTGTCGT<br>CGTACACGTTAGTGATGAACCTATCAAACTTGAAAGTGTAGTCGCTTGAGAACAGCGAGATGGAGTCCTTT<br>TCGTTGCCCTTGGCGGCCTCTTCCAGTTCCCCGGTCTGGTGAGCCATCAGGCTAATAGGAGTCTTGTCGGC<br>GAACAACCCTATCCCCGCGGAGAGGGTGAGCTTCCCGTTGGTCCACTTGATGAAGTTTTCGCGAAGTTCCA<br>CAGTGAACGCGATGATATCCTGCCACGATCCAATGGCGAACACGTCATCGCCGCCCGCGTAGATAATGCTC<br>AGCTTCTTGTCGGAGGCGAACTGGTTAATGTACACTTTGAAGAACAGCGACATGCTCCGGGAGAATGTGGC<br>CGATCTTGACAGAGTGGAGTATTGTCCGTTTCCCTGCTGGCTGAAACCGGCCATGAAGGCGGCGCCCAAGT<br>CATCCACGTCGAGCCGGACCACGGCCAGTCTCTTGATGCCTAGGCCGTTCTCGTTCTTGCTCAGGGCGGCG<br>TAGTTGTAGATCTCGTCGCACTGGTAATCCCCCACGAACACATGCGTAGCCTTCACGGTACCGGCCTTATA<br>GTCATTCTTCACGTAGACCCGGCTGAACGCTTCTTGGGACAGCTTTTCGAATGCCACGCCCTTTAAGCACG<br>CGTTTGGTCCAATCGGCAGCCCCTCATTTTCGGTAATGATGAAGTGGTCATGGGCAATTTCTTTCGAGAAC<br>TGGTACAGTCCCCGGCAAATGTCACAGACTTTCTGGTCGTGGTAGGACACCAGGTTCTCCACGGAGTGGCA

	AATCTCGCACTCTCTCTCTGAGGACTTTCCACCGCGATTCAGGAGCATCAGTGTCTGGTAGTCGTATCTGG<br>AGATTTTCTTTTTGGAGATCATGCGCGAAGCCTTTTGGTACACTTGGCGGTAGGACTCGGGGCTATTCAGC<br>TCGGACATGATGTCCTTGGCCGCGAAGGAACCCCAGCCAAAGGCCACATAGAGGCGGGTCTGGAAGTTTGC<br>CAACAGGAACTGGTTGAAATCCTTCTCAAACTGCACCAGGGTTTCCACAGTCTTTTCGGTGTTGGCCAGGA<br>CGAAGTAGGCGTGTCCGCCGCCGACGTAAAGCATGTTAGCCCTGTTCAGTCCCAGCTTGTCCAGCAGGCTA<br>TCGGCGATGTACTCGGACATAAAGTCCAGGTAGAGGCTCCGGGCCTTCAGCTGCTTCGCCGCGCCGTTAGT<br>TGCGATGTTAATGTTGTAGATAAAGTCCTGGATTCCCGACAGGTCGAAGGAGGCCAGCAGGAAGGCTTCTT<br>CTTCATAGAACGCTGACACTTTGGTGAACAGGTCCTCTTTGTAGTTGTGCCGACCCTTGTCCTCCAGGTAG<br>TCGTAGATCGCCAGAGCGAAGGCAGCAGTCAAGCGGAATGGTCGGCCAGGGAGATGTCGGCGATTTCCTT<br>AGTGTTGGTGCTTGACGGCACGAAGGAGAGAGTAGCCTCGAAAAGGTTGAGCAGGGAGTCAATCTGGACTT<br>GGTTGAACTCGAACTCGGCCAGTTCGTTCTTAATCCGGGTGGCGATGGCAGCGTAATCGCCCTTGCTAAAG<br>GGTTCGTAAGTGGCGGACGCGAAGTTGGGCTTCGACTTGAGATTCAGCACGGTAGGCTTGAAGTACCGCTT<br>ATCGGTCTGCGCTCCGAACACGTTAAAGATGTCGGCCTGGTTCGTGTAGGTGTCCCAGATCTTTGCGGAGG<br>TATCTTCGTCTGACTCTTCGTTGGATTGCCGGCGGTCGACACCGGAGGCGATGTTGTCGGCAATGTAGGTG<br>ATGTAAGCCAGGTGATCGTTGCCGAGCTTATCAGACTGGTAGTTGGCCATATGGTACCGGATCTGATCCGA<br>GATGACTTGGTTGTCGGCGATCTCGTCGAACCAGTCGGCGCCCACAAGTGCGTGTTTCTTCCGCTCTCCGG<br>TTGCTCGCTGGATGACCTTTCCGATGTCGTGCAGCAGGGCTCCGTAAAACAGATCAATCTTTTCTTTCTTC<br>ATGCCCCGCACTTTGCGTTTCTTCTTAGGCGCCATCTTATCGTCATCGTCTTTGTAATCGCCGGAATGGTG<br>ATGGTGATGGTGtggtcctggattttcttcaacatctccacaagttagcaaacttcctcttccttcGTCCT<br>TTCTTTCTTCCAGCTTGATGCCGCCCATTCCCAGGCTGGTTTTCATGCCCAGGCCGCTGTACTCGCCGAAG<br>GTCAGCAGCATCTTCACGTAGGCCTTCAGTGTCTGGGCTCCCTGCACCTTGAAGGTCAGCTTGCCCCGGAA<br>GGCGGGGATTCTCTGTCTATGCACTCTGAAGTAGCTTGTTTCCAGTCTGTAATTGGTGATGGTGCTGTGCT<br>CGCTCAGGTAGTCCAGGGTATCTTCCTCGATCTCAGGCTGGTTTTCCACCAGTCTGCCGTACTTCTGCATC<br>AGGCTCTGAAAGATCAGCCGCATGCTGGGGAAGATCACGTAGCTGCCCTGGCTTTTAAAGCCTGTAGGTGT<br>CTCGAAGATGATGCTGAAGTAGGTCTGGTTTTCCTCGCTGTTGAAGATCTCGAGCAGCTTATCAGCGCCCA<br>GCTCCTGGATTTCCACCTTTTCCACAGACAACTTAGGCAGGTCGTTCAGGGACACTTCCTTGATCTGCAGC<br>AGGGTCATGAACACCTTGTCCTCGATGTCGTCTGTCAGCAGATGCACGACCCACTGGGTGTTCTCTTTGCC<br>CTGGATCACCTTTGTGGCGTAGGGGTTGGTCTGCTGCTGGTGCAGGTAGTCCACGTAGTCGCTATCCAGCT<br>GTTCCATCAGGAAGCCGTGGAATTTCACGGCCAGGTCCTGAGCAGGGTGGTCGATCCGCTTAAAGGTGAAC<br>ACGAGCTTTTTCATGCCCCGCACTTTGCGTTTCTTCTTAGGCGCCATCTTATCGTCATCGTCTTTGTAATC<br>ggggccggggttctcctccacgtcgccgcaggtcagcaggctgcctctgccctcCTTGTACAGCTCGTCCA<br>TGCCGCCGGTGGAGTGGCGGCCCTCGGCGCGTTCGTACTGTTCCACGATGGTGTAGTCCTCGTTGTGGGAG<br>GTGATGTCCAACTTGATGTTGACGTTGTAGGCGCCGGGCAGCTGCACGGGCTTCTTGGCCTTGTAGGTGGT<br>CTTGACCTCAGCGTCGTAGTGGCCGCCGTCCTTCAGCTTCAGCCTCTGCTTGATCTCGCCCTTCAGGGCGC<br>CGTCCTCGGGGTACATCCGCTCGGAGGAGGCCTCCCAGCCCATGGTTTTCTTCTGCATTACGGGGCCGTCG<br>GAGGGGAAGTTGGTGCCGCGCAGCTTCACCTTGTAGATGAACTCGCCGTCCTGCAGGGAGGAGTCCTGGGT<br>CACGGTCACCACGCCGCCGTCCTCGAAGTTCATCACGCGCTCCCACTTGAAGCCCTCGGGGAAGGACAGCT<br>TCAAGTAGTCGGGGATGTCGGCGGGGTGCTTCACGTAGGCCTTGGAGCCGTACATGAACTGAGGGGACAGG<br>ATGTCCCAGGCGAAGGGCAGGGGGCCACCCTTGGTCACCTTCAGCTTGGCGGTCTGGGTGCCCTCGTAGGG<br>GCGGCCCTCGCCCTCGCCCTCGATCTCGAACTCGTGGCCGTTCACGGAGCCCTCCATGTGCACCTTGAAGC<br>GCATGAACTCCTTGATGATGGCCATGTTATCCTCCTCGCCCTTGCTCACCATggtggcggcACCGGTGAAT<br>TCTCCAGGCGATCTGACGGTTCACTAAACGAGCTCTGCTTATATAGGCCTCCCACCGTACACGCCACCTCG<br>ACATA

**Plasmid**	**pDAC435**
**Description**	Expression of Csm complex from separate promoters; RFP backbone
**Utility**	RNA KD
**Features**	Pcmv-FLAG-NLS-Csm1-pA; Pcmv-FLAG-NLS-Csm2-pA; Pcmv-FLAG-NLS-Csm3-pA; Pcmv-FLAG-NLS-Csm4-pA; Pcmv-FLAG-NLS-Csm5-pA; Pcmv-FLAG-NLS-Cas6-pA; Pu6-crRNA-pT; Pcmv-RFP-pA
**Sequence**	GTGATGCGGTTTTGGCAGTACATCAATGGGCGTGGATAGCGGTTTGACTCACGGGGATTTCCAAGTCTCCA<br>CCCCATTGACGTCAATGGGAGTTTGTTTTGGCACCAAAATCAACGGGACTTTCCAAAATGTCGTAACAACT<br>CCGCCCCATTGACGCAAATGGGCGGTAGGCGTGTACGGTGGGAGGTCTATATAAGCAGAGCTCGTTTAGTG<br>AACCGTCAGATCTCTAGAgccgccaccATGCACCATCACCATCACCATTCCGGCGATTACAAAGACGATGA<br>CGATAAGATGGCGCCTAAGAAGAAACGCAAAGTGCGGGGCATGAAGAAAGAAAAGATTGATCTGTTTTACG<br>GAGCCCTGCTGCACGACATCGGAAAGGTCATCCAGCGAGCAACCGGAGAGCGGAAGAAACACGCACTTGTG<br>GGCGCCGACTGGTTCGACGAGATCGCCGACAACCAAGTCATCTCGGATCAGATCCGGTACCATATGGCCAA<br>CTACCAGTCTGATAAGCTCGGCAACGATCACCTGGCTTACATCACCTACATTGCCGACAACATCGCCTCCG<br>GTGTCGACCGCCGGCAATCCAACGAAGAGTCAGACGAAGATACCTCCGCAAAGATCTGGGACACCTACACG<br>AACCAGGCCGACATCTTTAACGTGTTCGGAGCGCAGACCGATAAGCGGTACTTCAAGCCTACCGTGCTGAA<br>TCTCAAGTCGAAGCCCAACTTCGCGTCCGCCACTTACGAACCCTTTAGCAAGGGCGATTACGCTGCCATCG<br>CCACCCGGATTAAGAACGAACTGGCCGAGTTCGAGTTCAACCAAGTCCAGATTGACTCCCTGCTCAACCTT<br>TTCGAGGCTACTCTCTCCTTCGTGCCGTCAAGCACCAACACTAAGGAAATCGCCGACATCTCCCTGGCCGA

	CCATTCCCGCTTGACTGCTGCCTTCGCTCTGGCGATCTACGACTACCTGGAGGACAAGGGTCGGCACAACT<br>ACAAAGAGGACCTGTTCACCAAAGTGTCAGCGTTCTATGAAGAAGAAGCCTTCCTGCTGGCCTCCTTCGAC<br>CTGTCGGGAATCCAGGACTTTATCTACAACATTAACATCGCAACTAACGGCGCGGCGAAGCAGCTGAAGGC<br>CCGGAGCCTCTACCTGGACTTTATGTCCGAGTACATCGCCGATAGCCTGCTGGACAAGCTGGGACTGAACA<br>GGGCTAACATGCTTTACGTCGGCGGCGGACACGCCTACTTCGTCCTGGCCAACACCGAAAAGACTGTGGAA<br>ACCCTGGTGCAGTTTGAGAAGGATTTCAACCAGTTCCTGTTGGCAAACTTCCAGACCCGCCTCTATGTGGC<br>CTTTGGCTGGGGTTCCTTCGCGGCCAAGGACATCATGTCCGAGCTGAATAGCCCCGAGTCCTACCGCCAAG<br>TGTACCAAAAGGCTTCGCGCATGATCTCCAAAAAGAAAATCTCCAGATACGACTACCAGACACTGATGCTC<br>CTGAATCGCGGTGGAAAGTCCTCAGAGAGAGAGTGCGAGATTTGCCACTCCGTGGAGAACCTGGTGTCCTA<br>CCACGACCAGAAAGTCTGTGACATTTGCCGGGGACTGTACCAGTTCTCGAAAGAAATTGCCCATGACCACT<br>TCATCATTACCGAAAATGAGGGGCTGCCGATTGGACCAAACGCGTGCTTAAAGGGCGTGGCATTCGAAAAG<br>CTGTCCCAAGAAGCGTTCAGCCGGGTCTACGTGAAGAATGACTATAAGGCCGGTACCGTGAAGGCTACGCA<br>TGTGTTCGTGGGGGATTACCAGTGCGACGAGATCTACAACTACGCCGCCCTGAGCAAGAACGAGAACGGCC<br>TAGGCATCAAGAGACTGGCCGTGGTCCGGCTCGACGTGGATGACTTGGGCGCCGCCTTCATGGCCGGTTTC<br>AGCCAGCAGGGAAACGGACAATACTCCACTCTGTCAAGATCGGCCACATTCTCCCGGAGCATGTCGCTGTT<br>CTTCAAAGTGTACATTAACCAGTTCGCCTCCGACAAGAAGCTGAGCATTATCTACGCGGGCGGCGATGACG<br>TGTTCGCCATTGGATCGTGGCAGGATATCATCGCGTTCACTGTGGAACTTCGCGAAAACTTCATCAAGTGG<br>ACCAACGGGAAGCTCACCCTCTCCGCGGGGATAGGGTTGTTCGCCGACAAGACTCCTATTAGCCTGATGGC<br>TCACCAGACCGGGGAACTGGAAGAGGCCGCCAAGGGCAACGAAAAGGACTCCATCTCGCTGTTCTCAAGCG<br>ACTACACTTTCAAGTTTGATAGGTTCATCACTAACGTGTACGACGACAAACTGGAACAGATTAGATACTTC<br>TTCAACCATCAAGACGAGAGGGGAAAGAACTTCATCTATAAGCTTATTGAGCTTTTGAGGAACCACGACCG<br>CATGAATATGGCACGCCTCGCCTATTACCTCACTCGCCTGGAAGAACTGACCCGGGAGACTGACAGGGACA<br>AGTTCAAGACCTTCAAGAACCTGTTCTACTCCTGGTACACCAACAAGAACGATAAGGACCGGAAGGAAGCC<br>GAGCTCGCGCTCCTGCTGTACATCTACGAAATCAGAAAGGATTAAcggcaataaaaagacagaataaaacg<br>cacggtgttgggtcgtttgttcGCACACATTAGCTAGCCGTCAGCACACATTGTGATGCGGTTTTGGCAGT<br>ACATCAATGGGCGTGGATAGCGGTTTGACTCACGGGGATTTCCAAGTCTCCACCCCATTGACGTCAATGGG<br>AGTTTGTTTTGGCACCAAAATCAACGGGACTTTCCAAAATGTCGTAACAACTCCGCCCCATTGACGCAAAT<br>GGGCGGTAGGCGTGTACGGTGGGAGGTCTATATAAGCAGAGCTCGTTTAGTGAACCGTCAGATCTCTAGAg<br>ccgccaccATGGATTACAAAGACGATGACGATAAGATGGCGCCTAAGAAGAAACGCAAAGTGCGGGGCATG<br>ACCATCCTGACCGACGAGAACTACGTGGACATCGCCGAGAAAGCCATCCTGAAGCTGGAAAGAAACACCAG<br>AAATAGAAAGAACCCTGATGCCTTCTTCCTGACCACATCTAAGCTGCGGAACCTGCTGAGCCTGACAAGCA<br>CCCTGTTCGACGAGAGCAAGGTGAAGGAATACGACGCCCTGCTGGACAGAATCGCTTATCTGAGAGTGCAG<br>TTCGTGTACCAGGCCGGCAGAGAGATCGCCGTGAAAGATCTGATCGAGAAGGCCCAGATCCTGGAAGCTCT<br>GAAAGAGATCAAGGACCGGGAAACCCTGCAGAGATTCTGCAGATACATGGAAGCCCTGGTGGCCTACTTCA<br>AGTTCTACGGCGGCAAGGACTGAcggcaataaaaagacagaataaaacgcacggtgttgggtcgtttgttc<br>AGTTCTTTTGCCTTACTTTCAATGCATGCGGTGATGCGGTTTTGGCAGTACATCAATGGGCGTGGATAGCG<br>GTTTGACTCACGGGGATTTCCAAGTCTCCACCCCATTGACGTCAATGGGAGTTTGTTTTGGCACCAAAATC<br>AACGGGACTTTCCAAAATGTCGTAACAACTCCGCCCCATTGACGCAAATGGGCGGTAGGCGTGTACGGTGG<br>GAGGTCTATATAAGCAGAGCTCGTTTAGTGAACCGTCAGATCTCTAGAgccgccaccATGGATTACAAAGA<br>CGATGACGATAAGATGGCGCCTAAGAAGAAACGCAAAGTGCGGGGCATGACCTTCGCCAAGATCAAATTCA<br>GCGCCCAGATCCGGCTGGAAACCGGCCTGCACATCGGAGGATCTGATGCCTTTGCCGCTATCGGCGCCATC<br>GACAGCCCTGTGATCAAGGACCCCATCACCAACCTGCCTATCATCCCCGGCTCTAGCCTGAAGGGCAAGAT<br>GAGAACACTGCTGGCCAAGGTGTACAACGAAAAGGTGGCCGAGAAGCCTAGCGACGACAGCGACATCCTGA<br>GCAGACTGTTCGGAAATAGCAAGGATAAGCGGTTCAAGATGGGCAGACTGATCTTCCGGGACGCCTTCCTG<br>AGCAACGCCGACGAGCTGGATTCTCTGGGCGTGCGGAGCTACACCGAGGTGAAGTTCGAGAACACCATCGA<br>TAGAATCACCGCCGAGGCCAATCCTAGACAGATCGAGAGAGCCATTCGGAACTCAACATTCGACTTCGAGC<br>TGATCTACGAGATCACTGATGAGAATGAGAACCAGGTCGAGGAAGATTTCAAGGTGATCAGAGACGGCCTG<br>AAGCTGCTGGAACTGGACTACCTGGGCGGAAGCGGCTCCAGAGGCTACGGCAAAGTGGCTTTTGAGAACCT<br>GAAAGCCACCACAGTGTTCGGCAACTACGACGTGAAAACCCTGAACGAGCTGCTGACCGCCGAAGTGTGAc<br>ggcaataaaaagacagaataaaacgcacggtgttgggtcgtttgttcTGGATTGCGAGAATGGACTAGTAG<br>CAAACTGTGATGCGGTTTTGGCAGTACATCAATGGGCGTGGATAGCGGTTTGACTCACGGGGATTTCCAAG<br>TCTCCACCCCATTGACGTCAATGGGAGTTTGTTTTGGCACCAAAATCAACGGGACTTTCCAAAATGTCGTA<br>ACAACTCCGCCCCATTGACGCAAATGGGCGGTAGGCGTGTACGGTGGGAGGTCTATATAAGCAGAGCTCGT<br>TTAGTGAACCGTCAGATCTCTAGAgccgccaccATGGATTACAAAGACGATGACGATAAGATGGCGCCTAA<br>GAAGAAACGCAAAGTGCGGGGCATGACTTACAAGCTCTACATTATGACCTTTCAAAACGCCCACTTCGGTT<br>CCGGCACTCTGGACTCATCGAAGCTGACCTTCTCCGCGGATAGAATCTTCTCGGCACTCGTGCTCGAGGCT<br>CTGAAGATGGGAAAGCTCGACGCCTTCTTGGCCGAGGCCAACCAGGATAAGTTCACTCTGACCGACGCGTT<br>CCCATTCCAATTCGGTCCTTTCCTGCCGAAACCGATTGGTTACCCCAAGCACGACCAGATCGACCAGTCTG<br>TGGACGTGAAGGAAGTCCGCCGCCAAGCGAAGCTGTCCAAAAAGCTCCAGTTCCTGGCTCTGGAAAACGTC<br>GACGACTACCTGAACGGAGAGCTGTTTGAGAATGAGGAACACGCCGTGATCGACACAGTGACCAAGAACCA<br>GCCCCATAAAGATGATAATCTGTACCAAGTGGCCACCACTCGGTTCTCGAACGACACCTCCCTTTACGTGA<br>TCGCCAACGAATCCGATCTGCTGAACGAACTGATGAGCAGCCTTCAGTACTCCGGGCTGGGCGGCAAAAGG<br>TCCTCAGGATTCGGCAGATTTGAGCTGGACATCCAGAACATTCCCTTGGAACTGTCCGACCGGCTGACGAA

	GAACCACAGCGACAAGGTCATGTCACTTACCACCGCCCTCCCGGTGGACGCTGATCTCGAGGAAGCGATGG<br>AAGATGGCCATTACCTGTTGACCAAGTCGTCCGGATTCGCATTCTCCCACGCCACCAACGAAAACTATCGG<br>AAGCAGGACCTGTACAAGTTCGCCTCCGGGAGCACCTTCAGCAAGACTTTCGAGGGACAGATCGTGGACGT<br>GCGCCCTCTCGATTTCCCTCACGCCGTGCTGAACTACGCCAAGCCGCTGTTCTTTAAGCTCGAAGTCTAAc<br>ggcaataaaaagacagaataaaacgcacggtgttgggtcgtttgttcGCACATTCAAAAACAGGCAATTGG<br>ACAAGCGTGATGCGGTTTTGGCAGTACATCAATGGGCGTGGATAGCGGTTTGACTCACGGGGATTTCCAAG<br>TCTCCACCCCATTGACGTCAATGGGAGTTTGTTTTGGCACCAAAATCAACGGGACTTTCCAAAATGTCGTA<br>ACAACTCCGCCCCATTGACGCAAATGGGCGGTAGGCGTGTACGGTGGGAGGTCTATATAAGCAGAGCTCGT<br>TTAGTGAACCGTCAGATCTCTAGAgccgccaccATGGATTACAAAGACGATGACGATAAGATGGCGCCTAA<br>GAAGAAACGCAAAGTGCGGGGCATGAAAAATGACTACCGGACCTTCAAGCTGAGCCTGCTGACCCTGGCTC<br>CTATCCACATCGGCAACGGCGAGAAGTACACCAGCAGAGAATTCATCTACGAGAACAAGAAGTTCTACTTC<br>CCCGACATGGGCAAGTTCTACAACAAGATGGTGGAAAAGAGACTGGCCGAGAAGTTCGAGGCCTTCCTGAT<br>CCAGACCAGACCCAACGCCAGAAACAACCGGCTGATTTCTTTTCTGAACGACAACAGAATCGCCGAAAGAT<br>CTTTTGGCGGCTACAGCATCAGTGAAACCGGCCTGGAATCTGATAAGAACCCTAACAGCGCCGGAGCTATC<br>AACGAGGTGAACAAATTCATCCGGGACGCCTTCGGAAATCCTTACATCCCAGGCAGCAGCCTGAAGGGCGC<br>CATCCGCACCATCCTGATGAACACCACACCTAAGTGGAACAACGAGAACGCCGTGAACGACTTCGGCAGAT<br>TCCCAAAGGAAAACAAGAACCTGATCCCTTGGGGACCTAAGAAAGGCAAGGAATACGACGACCTGTTCAAC<br>GCCATCAGAGTGTCCGACAGCAAGCCCTTCGACAACAAAAGCCTGATCCTCGTGCAGAAGTGGGACTACAG<br>CGCCAAAACCAACAAGGCCAAGCCTCTGCCTCTGTACAGAGAGTCTATCAGCCCTCTGACCAAGATCGAGT<br>TCGAGATAACAACAACCACTGATGAGGCCGGCAGACTGATCGAGGAACTGGGAAAGCGGGCCCAGGCCTTT<br>TATAAGGACTACAAGGCCTTTTTCCTGTCTGAATTCCCTGATGATAAGATCCAGGCTAATCTGCAATACCC<br>CATCTACCTGGGCGCCGGCAGCGGCGCTTGGACAAAGACCCTGTTTAAGCAGGCCGACGGCATCCTGCAGC<br>GGAGATACTCCAGAATGAAAACCAAGATGGTCAAGAAGGGCGTGCTGAAGCTGACAAAGGCCCCTCTGAAA<br>ACAGTGAAGATCCCCAGCGGCAACCACAGCCTGGTGAAGAATCACGAGAGCTTCTACGAGATGGGCAAAGC<br>CAACTTCATGATCAAGGAAATCGACAAGTGAcggcaataaaaagacagaataaaacgcacggtgttgggtc<br>gtttgttcATGTGTGCCGGCCGGAGAATAAAAGTCTAAGTGATGCGGTTTTGGCAGTACATCAATGGGCGT<br>GGATAGCGGTTTGACTCACGGGGATTTCCAAGTCTCCACCCCATTGACGTCAATGGGAGTTTGTTTTGGCA<br>CCAAAATCAACGGGACTTTCCAAAATGTCGTAACAACTCCGCCCCATTGACGCAAATGGGCGGTAGGCGTG<br>TACGGTGGGAGGTCTATATAAGCAGAGCTCGTTTAGTGAACCGTCAGATCTCTAGAgccgccaccATGGAT<br>TACAAAGACGATGACGATAAGATGGCGCCTAAGAAGAAACGCAAAGTGCGGGGCATGAAAAAGCTCGTGTT<br>CACCTTTAAGCGGATCGACCACCCTGCTCAGGACCTGGCCGTGAAATTCCACGGCTTCCTGATGGAACAGC<br>TGGATAGCGACTACGTGGACTACCTGCACCAGCAGCAGACCAACCCCTACGCCACAAAGGTGATCCAGGGC<br>AAAGAGAACACCCAGTGGGTCGTGCATCTGCTGACAGACGACATCGAGGACAAGGTGTTCATGACCCTGCT<br>GCAGATCAAGGAAGTGTCCCTGAACGACCTGCCTAAGTTGTCTGTGGAAAAGGTGGAAATCCAGGAGCTGG<br>GCGCTGATAAGCTGCTCGAGATCTTCAACAGCGAGGAAAACCAGACCTACTTCAGCATCATCTTCGAGACA<br>CCTACAGGCTTTAAAAGCCAGGGCAGCTACGTGATCTTCCCCAGCATGCGGCTGATCTTTCAGAGCCTGAT<br>GCAGAAGTACGGCAGACTGGTGGAAAACCAGCCTGAGATCGAGGAAGATACCCTGGACTACCTGAGCGAGC<br>ACAGCACCATCACCAATTACAGACTGGAAACAAGCTACTTCAGAGTGCATAGACAGAGAATCCCCGCCTTC<br>CGGGGCAAGCTGACCTTCAAGGTGCAGGGAGCCCAGACACTGAAGGCCTACGTGAAGATGCTGCTGACCTT<br>CGGCGAGTACAGCGGCCTGGGCATGAAAACCAGCCTGGGAATGGGCGGCATCAAGCTGGAAGAAAGAAAGG<br>ACTGAcggcaataaaaagacagaataaaacgcacggtgttgggtcgtttgttcGTGCGATAGAGGGATCCC<br>GCATTGAATTATGTGATGCGGTTTTGGCAGTACATCAATGGGCGTGGATAGCGGTTTGACTCACGGGGATT<br>TCCAAGTCTCCACCCCATTGACGTCAATGGGAGTTTGTTTTGGCACCAAAATCAACGGGACTTTCCAAAAT<br>GTCGTAACAACTCCGCCCCATTGACGCAAATGGGCGGTAGGCGTGTACGGTGGGAGGTCTATATAAGCAGA<br>GCTCGTTTAGTGAACCGTCAGATCTCTAGAgccgccaccATGGTGAGCAAGGGCGAGGAGGATAACATGGC<br>CATCATCAAGGAGTTCATGCGCTTCAAGGTGCACATGGAGGGCTCCGTGAACGGCCACGAGTTCGAGATCG<br>AGGGCGAGGGCGAGGGCCGCCCCTACGAGGGCACCCAGACCGCCAAGCTGAAGGTGACCAAGGGTGGCCCC<br>CTGCCCTTCGCCTGGGACATCCTGTCCCCTCAGTTCATGTACGGCTCCAAGGCCTACGTGAAGCACCCCGC<br>CGACATCCCCGACTACTTGAAGCTGTCCTTCCCCGAGGGCTTCAAGTGGGAGCGCGTGATGAACTTCGAGG<br>ACGGCGGCGTGGTGACCGTGACCCAGGACTCCTCCCTGCAGGACGGCGAGTTCATCTACAAGGTGAAGCTG<br>CGCGGCACCAACTTCCCCTCCGACGGCCCCGTAATGCAGAAGAAAACCATGGGCTGGGAGGCCTCCTCCGA<br>GCGGATGTACCCCGAGGACGGCGCCCTGAAGGGCGAGATCAAGCAGAGGCTGAAGCTGAAGGACGGCGGCC<br>ACTACGACGCTGAGGTCAAGACCACCTACAAGGCCAAGAAGCCCGTGCAGCTGCCCGGCGCCTACAACGTC<br>AACATCAAGTTGGACATCACCTCCCACAACGAGGACTACACCATCGTGGAACAGTACGAACGCGCCGAGGG<br>CCGCCACTCCACCGGCGGCATGGACGAGCTGTACAAGTAAcggcaataaaaagacagaataaaacgcacgg<br>tgttgggtcgtttgttcGAGCAGATTGTACTGAGAGTGCACCGGTTGGAGGGCCTATTTCCCATGATTCCT<br>TCATATTTGCATATACGATACAAGGCTGTTAGAGAGATAATTAGAATTAATTTGACTGTAAACACAAAGAT<br>ATTAGTACAAAATACGTGACGTAGAAAGTAATAATTTCTTGGGTAGTTTGCAGTTTTAAAATTATGTTTTA<br>AAATGGACTATCATATGCTTACCGTAACTTGAAAGTATTTCGATTTCTTGGCTTTATATATCTTGTGGAAA<br>GGACGAAACACCGATATAAACCTAATTACCTCGAGAGGGGACGGAAACCCGTCTTCGATGAAGCGATTCAG<br>AAGACTTGATATAAACCTAATTACCTCGAGAGGGGACTTTTTTACATGTGTCAGAGGTTTTCACCGTCATC<br>ACCGAAACGCGCGAGACGAAAGGCCTCGTGATACGCCTATTTTTATAGGTTAATGTCATGATAATAATGG<br>TTTCTTAGACGTCAGGTGGCACTTTTCGGGGAAATGTGCGCGGAACCCCTATTTGTTTATTTTTCTAAATA

	CATTCAAATATGTATCCGCTCATGAGACAATAACCCTGATAAATGCTTCAATAATATTGAAAAAGGAAGAG<br>TATGAGTATTCAACATTTCCGTGTCGCCCTTATTCCCTTTTTTGCGGCATTTTGCCTTCCTGTTTTTGCTC<br>ACCCAGAAACGCTGGTGAAAGTAAAAGATGCTGAAGATCAGTTGGGTGCACGAGTGGGTTACATCGAACTG<br>GATCTCAACAGCGGTAAGATCCTTGAGAGTTTTCGCCCCGAAGAACGTTTTCCAATGATGAGCACTTTTAA<br>AGTTCTGCTATGTGGCGCGGTATTATCCCGTATTGACGCCGGGCAAGAGCAACTCGGTCGCCGCATACACT<br>ATTCTCAGAATGACTTGGTTGAGTACTCACCAGTCACAGAAAAGCATCTTACGGATGGCATGACAGTAAGA<br>GAATTATGCAGTGCTGCCATAACCATGAGTGATAACACTGCGGCCAACTTACTTCTGACAACGATCGGAGG<br>ACCGAAGGAGCTAACCGCTTTTTTGCACAACATGGGGGATCATGTAACTCGCCTTGATCGTTGGGAACCGG<br>AGCTGAATGAAGCCATACCAAACGACGAGCGTGACACCACGATGCCTGTAGCAATGGCAACAACGTTGCGC<br>AAACTATTAACTGGCGAACTACTTACTCTAGCTTCCCGGCAACAATTAATAGACTGGATGGAGGCGGATAA<br>AGTTGCAGGACCACTTCTGCGCTCGGCCCTTCCGGCTGGCTGGTTTATTGCTGATAAATCTGGAGCCGGTG<br>AGCGTGGGTCTCGCGGTATCATTGCAGCACTGGGGCCAGATGGTAAGCCCTCCCGTATCGTAGTTATCTAC<br>ACGACGGGGAGTCAGGCAACTATGGATGAACGAAATAGACAGATCGCTGAGATAGGTGCCTCACTGATTAA<br>GCATTGGTAACTGTCAGACCAAGTTTACTCATATATACTTTAGATTGATTTAAAACTTCATTTTTAATTTA<br>AAAGGATCTAGGTGAAGATCCTTTTTGATAATCTCATGACCAAAATCCCTTAACGTGAGTTTTCGTTCCAC<br>TGAGCGTCAGACCCCGTAGAAAAGATCAAAGGATCTTCTTGAGATCCTTTTTTTCTGCGCGTAATCTGCTG<br>CTTGCAAACAAAAAAACCACCGCTACCAGCGGTGGTTTGTTTGCCGGATCAAGAGCTACCAACTCTTTTTC<br>CGAAGGTAACTGGCTTCAGCAGAGCGCAGATACCAAATACTGTTCTTCTAGTGTAGCCGTAGTTAGGCCAC<br>CACTTCAAGAACTCTGTAGCACCGCCTACATACCTCGCTCTGCTAATCCTGTTACCAGTGGCTGCTGCCAG<br>TGGCGATAAGTCGTGTCTTACCGGGTTGGACTCAAGACGATAGTTACCGGATAAGGCGCAGCGGTCGGGCT<br>GAACGGGGGGTTCGTGCACACAGCCCAGCTTGGAGCGAACGACCTACACCGAACTGAGATACCTACAGCGT<br>GAGCTATGAGAAAGCGCCACGCTTCCCGAAGGGAGAAAGGCGGACAGGTATCCGGTAAGCGGCAGGGTCGG<br>AACAGGAGAGCGCACGAGGGAGCTTCCAGGGGGAAACGCCTGGTATCTTTATAGTCCTGTCGGGTTTCGCC<br>ACCTCTGACTTGAGCGTCGATTTTTGTGATGCTCGTCAGGGGGGCGGAGCCTATGGAAAAACGCCAGCAAC<br>GCGGCCTTTTTCTTAAGC

**Plasmid**	**pDAC446**
**Description**	Expression of Csm complex from separate promoters; GFP backbone
**Utility**	RNA KD
**Features**	Pcmv-FLAG-NLS-Csm1-pA; Pcmv-FLAG-NLS-Csm2-pA; Pcmv-FLAG-NLS-Csm3-pA; Pcmv-FLAG-NLS-Csm4-pA; Pcmv-FLAG-NLS-Csm5-pA; Pcmv-FLAG-NLS-Cas6-pA; Pu6-crRNA-pT; Pcmv-GFP-pA
**Sequence**	GTGATGCGGTTTTGGCAGTACATCAATGGGCGTGGATAGCGGTTTGACTCACGGGGATTTCCAAGTCTCCA<br>CCCCATTGACGTCAATGGGAGTTTGTTTTGGCACCAAAATCAACGGGACTTTCCAAAATGTCGTAACAACT<br>CCGCCCCATTGACGCAAATGGGCGGTAGGCGTGTACGGTGGGAGGTCTATATAAGCAGAGCTCGTTTAGTG<br>AACCGTCAGATCTCTAGAgccgccaccATGCACCATCACCATCACCATTCCGGCGATTACAAAGACGATGA<br>CGATAAGATGGCGCCTAAGAAGAAACGCAAAGTGCGGGGCATGAAGAAAGAAAAGATTGATCTGTTTTACG<br>GAGCCCTGCTGCACGACATCGGAAAGGTCATCCAGCGAGCAACCGGAGAGCGGAAGAAACACGCACTTGTG<br>GGCGCCGACTGGTTCGACGAGATCGCCGACAACCAAGTCATCTCGGATCAGATCCGGTACCATATGGCCAA<br>CTACCAGTCTGATAAGCTCGGCAACGATCACCTGGCTTACATCACCTACATTGCCGACAACATCGCCTCCG<br>GTGTCGACCGCCGGCAATCCAACGAAGAGTCAGACGAAGATACCTCCGCAAAGATCTGGGACACCTACACG<br>AACCAGGCCGACATCTTTAACGTGTTCGGAGCGCAGACCGATAAGCGGTACTTCAAGCCTACCGTGCTGAA<br>TCTCAAGTCGAAGCCCAACTTCGCGTCCGCCACTTACGAACCCTTTAGCAAGGGCGATTACGCTGCCATCG<br>CCACCCGGATTAAGAACGAACTGGCCGAGTTCGAGTTCAACCAAGTCCAGATTGACTCCCTGCTCAACCTT<br>TTCGAGGCTACTCTCTCCTTCGTGCCGTCAAGCACCAACACTAAGGAAATCGCCGACATCTCCCTGGCCGA<br>CCATTCCCGCTTGACTGCTGCCTTCGCTCTGGCGATCTACGACTACCTGGAGGACAAGGGTCGGCACAACT<br>ACAAAGAGGACCTGTTCACCAAAGTGTCAGCGTTCTATGAAGAAGAAGCCTTCCTGCTGGCCTCCTTCGAC<br>CTGTCGGGAATCCAGGACTTTATCTACAACATTAACATCGCAACTAACGGCGCGGCGAAGCAGCTGAAGGC<br>CCGGAGCCTCTACCTGGACTTTATGTCCGAGTACATCGCCGATAGCCTGCTGGACAAGCTGGGACTGAACA<br>GGGCTAACATGCTTTACGTCGGCGGCGGACACGCCTACTTCGTCCTGGCCAACACCGAAAAGACTGTGGAA<br>ACCCTGGTGCAGTTTGAGAAGGATTTCAACCAGTTCCTGTTGGCAAACTTCCAGACCCGCCTCTATGTGGC<br>CTTTGGCTGGGGTTCCTTCGCGGCCAAGGACATCATGTCCGAGCTGAATAGCCCCGAGTCCTACCGCCAAG<br>TGTACCAAAAGGCTTCGCGCATGATCTCCAAAAAGAAAATCTCCAGATACGACTACCAGACACTGATGCTC<br>CTGAATCGCGGTGGAAAGTCCTCAGAGAGAGAGTGCGAGATTTGCCACTCCGTGGAGAACCTGGTGTCCTA<br>CCACGACCAGAAAGTCTGTGACATTTGCCGGGGACTGTACCAGTTCTCGAAAGAAATTGCCCATGACCACT<br>TCATCATTACCGAAAATGAGGGGCTGCCGATTGGACCAAACGCGTGCTTAAAGGGCGTGGCATTCGAAAAG<br>CTGTCCCAAGAAGCGTTCAGCCGGGTCTACGTGAAGAATGACTATAAGGCCGGTACCGTGAAGGCTACGCA<br>TGTGTTCGTGGGGGATTACCAGTGCGACGAGATCTACAACTACGCCGCCCTGAGCAAGAACGAGAACGGCC<br>TAGGCATCAAGAGACTGGCCGTGGTCCGGCTCGACGTGGATGACTTGGGCGCCGCCTTCATGGCCGGTTTC<br>AGCCAGCAGGGAAACGGACAATACTCCACTCTGTCAAGATCGGCCACATTCTCCCGGAGCATGTCGCTGTT<br>CTTCAAAGTGTACATTAACCAGTTCGCCTCCGACAAGAAGCTGAGCATTATCTACGCGGGCGGCGATGACG<br>TGTTCGCCATTGGATCGTGGCAGGATATCATCGCGTTCACTGTGGAACTTCGCGAAAACTTCATCAAGTGG<br>ACCAACGGGAAGCTCACCCTCTCCGCGGGGATAGGGTTGTTCGCCGACAAGACTCCTATTAGCCTGATGGC

	TCACCAGACCGGGGAACTGGAAGAGGCCGCCAAGGGCAACGAAAAGGACTCCATCTCGCTGTTCTCAAGCG<br>ACTACACTTTCAAGTTTGATAGGTTCATCACTAACGTGTACGACGACAAACTGGAACAGATTAGATACTTC<br>TTCAACCATCAAGACGAGAGGGGAAAGAACTTCATCTATAAGCTTATTGAGCTTTTGAGGAACCACGACCG<br>CATGAATATGGCACGCCTCGCCTATTACCTCACTCGCCTGGAAGAACTGACCCGGGAGACTGACAGGGACA<br>AGTTCAAGACCTTCAAGAACCTGTTCTACTCCTGGTACACCAACAAGAACGATAAGGACCGGAAGGAAGCC<br>GAGCTCGCGCTCCTGCTGTACATCTACGAAATCAGAAAGGATTAAcggcaataaaaagacagaataaaacg<br>cacggtgttgggtcgtttgttcGCACACATTAGCTAGCCGTCAGCACACATTGTGATGCGGTTTTGGCAGT<br>ACATCAATGGGCGTGGATAGCGGTTTGACTCACGGGGATTTCCAAGTCTCCACCCCATTGACGTCAATGGG<br>AGTTTGTTTTGGCACCAAAATCAACGGGACTTTCCAAAATGTCGTAACAACTCCGCCCCATTGACGCAAAT<br>GGGCGGTAGGCGTGTACGGTGGGAGGTCTATATAAGCAGAGCTCGTTTAGTGAACCGTCAGATCTCTAGAg<br>ccgccaccATGGATTACAAAGACGATGACGATAAGATGGCGCCTAAGAAGAAACGCAAAGTGCGGGGCATG<br>ACCATCCTGACCGACGAGAACTACGTGGACATCGCCGAGAAAGCCATCCTGAAGCTGGAAAGAAACACCAG<br>AAATAGAAAGAACCCTGATGCCTTCTTCCTGACCACATCTAAGCTGCGGAACCTGCTGAGCCTGACAAGCA<br>CCCTGTTCGACGAGAGCAAGGTGAAGGAATACGACGCCCTGCTGGACAGAATCGCTTATCTGAGAGTGCAG<br>TTCGTGTACCAGGCCGGCAGAGAGATCGCCGTGAAAGATCTGATCGAGAAGGCCCAGATCCTGGAAGCTCT<br>GAAAGAGATCAAGGACCGGGAAACCCTGCAGAGATTCTGCAGATACATGGAAGCCCTGGTGGCCTACTTCA<br>AGTTCTACGGCGGCAAGGACTGAcggcaataaaaagacagaataaaacgcacggtgttgggtcgtttgttc<br>AGTTCTTTTGCCTTACTTTCAATGCATGCGGTGATGCGGTTTTGGCAGTACATCAATGGGCGTGGATAGCG<br>GTTTGACTCACGGGGATTTCCAAGTCTCCACCCCATTGACGTCAATGGGAGTTTGTTTTGGCACCAAAATC<br>AACGGGACTTTCCAAAATGTCGTAACAACTCCGCCCCATTGACGCAAATGGGCGGTAGGCGTGTACGGTGG<br>GAGGTCTATATAAGCAGAGCTCGTTTAGTGAACCGTCAGATCTCTAGAgccgccaccATGGATTACAAAGA<br>CGATGACGATAAGATGGCGCCTAAGAAGAAACGCAAAGTGCGGGGCATGACCTTCGCCAAGATCAAATTCA<br>GCGCCCAGATCCGGCTGGAAACCGGCCTGCACATCGGAGGATCTGATGCCTTTGCCGCTATCGGCGCCATC<br>GACAGCCCTGTGATCAAGGACCCCATCACCAACCTGCCTATCATCCCCGGCTCTAGCCTGAAGGGCAAGAT<br>GAGAACACTGCTGGCCAAGGTGTACAACGAAAAGGTGGCCGAGAAGCCTAGCGACGACAGCGACATCCTGA<br>GCAGACTGTTCGGAAATAGCAAGGATAAGCGGTTCAAGATGGGCAGACTGATCTTCCGGGACGCCTTCCTG<br>AGCAACGCCGACGAGCTGGATTCTCTGGGCGTGCGGAGCTACACCGAGGTGAAGTTCGAGAACACCATCGA<br>TAGAATCACCGCCGAGGCCAATCCTAGACAGATCGAGAGAGCCATTCGGAACTCAACATTCGACTTCGAGC<br>TGATCTACGAGATCACTGATGAGAATGAGAACCAGGTCGAGGAAGATTTCAAGGTGATCAGAGACGGCCTG<br>AAGCTGCTGGAACTGGACTACCTGGGCGGAAGCGGCTCCAGAGGCTACGGCAAAGTGGCTTTTGAGAACCT<br>GAAAGCCACCACAGTGTTCGGCAACTACGACGTGAAAACCCTGAACGAGCTGCTGACCGCCGAAGTGTGAc<br>ggcaataaaaagacagaataaaacgcacggtgttgggtcgtttgttcTGGATTGCGAGAATGGACTAGTAG<br>CAAACTGTGATGCGGTTTTGGCAGTACATCAATGGGCGTGGATAGCGGTTTGACTCACGGGGATTTCCAAG<br>TCTCCACCCCATTGACGTCAATGGGAGTTTGTTTTGGCACCAAAATCAACGGGACTTTCCAAAATGTCGTA<br>ACAACTCCGCCCCATTGACGCAAATGGGCGGTAGGCGTGTACGGTGGGAGGTCTATATAAGCAGAGCTCGT<br>TTAGTGAACCGTCAGATCTCTAGAgccgccaccATGGATTACAAAGACGATGACGATAAGATGGCGCCTAA<br>GAAGAAACGCAAAGTGCGGGGCATGACTTACAAGCTCTACATTATGACCTTTCAAAACGCCCACTTCGGTT<br>CCGGCACTCTGGACTCATCGAAGCTGACCTTCTCCGCGGATAGAATCTTCTCGGCACTCGTGCTCGAGGCT<br>CTGAAGATGGGAAAGCTCGACGCCTTCTTGGCCGAGGCCAACCAGGATAAGTTCACTCTGACCGACGCGTT<br>CCCATTCCAATTCGGTCCTTTCCTGCCGAAACCGATTGGTTACCCCAAGCACGACCAGATCGACCAGTCTG<br>TGGACGTGAAGGAAGTCCGCCGCCAAGCGAAGCTGTCCAAAAAGCTCCAGTTCCTGGCTCTGGAAAACGTC<br>GACGACTACCTGAACGGAGAGCTGTTTGAGAATGAGGAACACGCCGTGATCGACACAGTGACCAAGAACCA<br>GCCCCATAAAGATGATAATCTGTACCAAGTGGCCACCACTCGGTTCTCGAACGACACCTCCCTTTACGTGA<br>TCGCCAACGAATCCGATCTGCTGAACGAACTGATGAGCAGCCTTCAGTACTCCGGGCTGGGCGGCAAAAGG<br>TCCTCAGGATTCGGCAGATTTGAGCTGGACATCCAGAACATTCCCTTGGAACTGTCCGACCGGCTGACGAA<br>GAACCACAGCGACAAGGTCATGTCACTTACCACCGCCCTCCCGGTGGACGCTGATCTCGAGGAAGCGATGG<br>AAGATGGCCATTACCTGTTGACCAAGTCGTCCGGATTCGCATTCTCCCACGCCACCAACGAAAACTATCGG<br>AAGCAGGACCTGTACAAGTTCGCCTCCGGGAGCACCTTCAGCAAGACTTTCGAGGGACAGATCGTGGACGT<br>GCGCCCTCTCGATTTCCCTCACGCCGTGCTGAACTACGCCAAGCCGCTGTTCTTTAAGCTCGAAGTCTAAc<br>ggcaataaaaagacagaataaaacgcacggtgttgggtcgtttgttcGCACATTCAAAAACAGGCAATTGG<br>ACAAGCGTGATGCGGTTTTGGCAGTACATCAATGGGCGTGGATAGCGGTTTGACTCACGGGGATTTCCAAG<br>TCTCCACCCCATTGACGTCAATGGGAGTTTGTTTTGGCACCAAAATCAACGGGACTTTCCAAAATGTCGTA<br>ACAACTCCGCCCCATTGACGCAAATGGGCGGTAGGCGTGTACGGTGGGAGGTCTATATAAGCAGAGCTCGT<br>TTAGTGAACCGTCAGATCTCTAGAgccgccaccATGGATTACAAAGACGATGACGATAAGATGGCGCCTAA<br>GAAGAAACGCAAAGTGCGGGGCATGAAAAATGACTACCGGACCTTCAAGCTGAGCCTGCTGACCCTGGCTC<br>CTATCCACATCGGCAACGGCGAGAAGTACACCAGCAGAGAATTCATCTACGAGAACAAGAAGTTCTACTTC<br>CCCGACATGGGCAAGTTCTACAACAAGATGGTGGAAAAGAGACTGGCCGAGAAGTTCGAGGCCTTCCTGAT<br>CCAGACCAGACCCAACGCCAGAAACAACCGGCTGATTTCTTTTCTGAACGACAACAGAATCGCCGAAAGAT<br>CTTTTGGCGGCTACAGCATCAGTGAAACCGGCCTGGAATCTGATAAGAACCCTAACAGCGCCGGAGCTATC<br>AACGAGGTGAACAAATTCATCCGGGACGCCTTCGGAAATCCTTACATCCCAGGCAGCAGCCTGAAGGGCGC<br>CATCCGCACCATCCTGATGAACACCACACCTAAGTGGAACAACGAGAACGCCGTGAACGACTTCGGCAGAT<br>TCCCAAAGGAAAACAAGAACCTGATCCCTTGGGGACCTAAGAAAGGCAAGGAATACGACGACCTGTTCAAC<br>GCCATCAGAGTGTCCGACAGCAAGCCCTTCGACAACAAAAGCCTGATCCTCGTGCAGAAGTGGGACTACAG

	CGCCAAAACCAACAAGGCCAAGCCTCTGCCTCTGTACAGAGAGTCTATCAGCCCTCTGACCAAGATCGAGT<br>TCGAGATAACAACAACCACTGATGAGGCCGGCAGACTGATCGAGGAACTGGGAAAGCGGGCCCAGGCCTTT<br>TATAAGGACTACAAGGCCTTTTTCCTGTCTGAATTCCCTGATGATAAGATCCAGGCTAATCTGCAATACCC<br>CATCTACCTGGGCGCCGGCAGCGGCGCTTGGACAAAGACCCTGTTTAAGCAGGCCGACGGCATCCTGCAGC<br>GGAGATACTCCAGAATGAAAACCAAGATGGTCAAGAAGGGCGTGCTGAAGCTGACAAAGGCCCCTCTGAAA<br>ACAGTGAAGATCCCCAGCGGCAACCACAGCCTGGTGAAGAATCACGAGAGCTTCTACGAGATGGGCAAAGC<br>CAACTTCATGATCAAGGAAATCGACAAGTGAcggcaataaaaagacagaataaaacgcacggtgttgggtc<br>gtttgttcATGTGTGCCGGCCGGAGAATAAAAGTCTAAGTGATGCGGTTTTGGCAGTACATCAATGGGCGT<br>GGATAGCGGTTTGACTCACGGGGATTTCCAAGTCTCCACCCCATTGACGTCAATGGGAGTTTGTTTTGGCA<br>CCAAAATCAACGGGACTTTCCAAAATGTCGTAACAACTCCGCCCCATTGACGCAAATGGGCGGTAGGCGTG<br>TACGGTGGGAGGTCTATATAAGCAGAGCTCGTTTAGTGAACCGTCAGATCTCTAGAgccgccaccATGGAT<br>TACAAAGACGATGACGATAAGATGGCGCCTAAGAAGAAACGCAAAGTGCGGGGCATGAAAAAGCTCGTGTT<br>CACCTTTAAGCGGATCGACCACCCTGCTCAGGACCTGGCCGTGAAATTCCACGGCTTCCTGATGGAACAGC<br>TGGATAGCGACTACGTGGACTACCTGCACCAGCAGCAGACCAACCCCTACGCCACAAAGGTGATCCAGGGC<br>AAAGAGAACACCCAGTGGGTCGTGCATCTGCTGACAGACGACATCGAGGACAAGGTGTTCATGACCCTGCT<br>GCAGATCAAGGAAGTGTCCCTGAACGACCTGCCTAAGTTGTCTGTGGAAAAGGTGGAAATCCAGGAGCTGG<br>GCGCTGATAAGCTGCTCGAGATCTTCAACAGCGAGGAAAACCAGACCTACTTCAGCATCATCTTCGAGACA<br>CCTACAGGCTTTAAAAGCCAGGGCAGCTACGTGATCTTCCCCAGCATGCGGCTGATCTTTCAGAGCCTGAT<br>GCAGAAGTACGGCAGACTGGTGGAAAACCAGCCTGAGATCGAGGAAGATACCCTGGACTACCTGAGCGAGC<br>ACAGCACCATCACCAATTACAGACTGGAAACAAGCTACTTCAGAGTGCATAGACAGAGAATCCCCGCCTTC<br>CGGGGCAAGCTGACCTTCAAGGTGCAGGGAGCCCAGACACTGAAGGCCTACGTGAAGATGCTGCTGACCTT<br>CGGCGAGTACAGCGGCCTGGGCATGAAAACCAGCCTGGGAATGGGCGGCATCAAGCTGGAAGAAAGAAAGG<br>ACTGAcggcaataaaaagacagaataaaacgcacggtgttgggtcgtttgttcGTGCGATAGAGGGATCCC<br>GCATTGAATTATGTGATGCGGTTTTGGCAGTACATCAATGGGCGTGGATAGCGGTTTGACTCACGGGGATT<br>TCCAAGTCTCCACCCCATTGACGTCAATGGGAGTTTGTTTTGGCACCAAAATCAACGGGACTTTCCAAAAT<br>GTCGTAACAACTCCGCCCCATTGACGCAAATGGGCGGTAGGCGTGTACGGTGGGAGGTCTATATAAGCAGA<br>GCTCGTTTAGTGAACCGTCAGATCTCTAGAgccgccaccatggtgagcaagggcgaggagctgttcaccgg<br>ggtggtgcccatcctggtcgagctggacggcgacgtaaacggccacaagttcagcgtgtccggcgagggcg<br>agggcgatgccacctacggcaagctgaccctgaagttcatctgcaccaccggcaagctgcccgtgccctgg<br>cccaccctcgtgaccaccctgacctacggcgtgcagtgcttcagccgctaccccgaccacatgaagcagca<br>cgacttcttcaagtccgccatgcccgaaggctacgtccaggagcgcaccatcttcttcaaggacgacggca<br>actacaagacccgcgccgaggtgaagttcgagggcgacaccctggtgaaccgcatcgagctgaagggcatc<br>gacttcaaggaggacggcaacatcctggggcacaagctggagtacaactacaacagccacaacgtctatat<br>catggccgacaagcagaagaacggcatcaaggtgaacttcaagatccgccacaacatcgaggacggcagcg<br>tgcagctcgccgaccactaccagcagaacacccccatcggcgacggccccgtgctgctgcccgacaaccac<br>tacctgagcacccagtccgccctgagcaaagaccccaacgagaagcgcgatcacatggtcctgctggagtt<br>cgtgaccgccgccgggatcactctcggcatggacgagctgtacaagtgacggcaataaaaagacagaataa<br>aacgcacggtgttgggtcgtttgttcGTAGATGGCGCGCCTTTGTTGACCCGGTTGGAGGGCCTATTTCCC<br>ATGATTCCTTCATATTTGCATATACGATACAAGGCTGTTAGAGAGATAATTAGAATTAATTTGACTGTAAA<br>CACAAAGATATTAGTACAAAATACGTGACGTAGAAAGTAATAATTTCTTGGGTAGTTTGCAGTTTTAAAAT<br>TATGTTTTAAAATGGACTATCATATGCTTACCGTAACTTGAAAGTATTTCGATTTCTTGGCTTTATATATC<br>TTGTGGAAAGGACGAAACACCGATATAAACCTAATTACCTCGAGAGGGGACGGAAACCCGTCTTCGATGAA<br>GCGATTCAGAAGACTTGATATAAACCTAATTACCTCGAGAGGGGACTTTTTTACATGTGTCAGAGGTTTTC<br>ACCGTCATCACCGAAACGCGCGAGACGAAAGGGCCTCGTGATACGCCTATTTTTATAGGTTAATGTCATGA<br>TAATAATGGTTTCTTAGACGTCAGGTGGCACTTTTCGGGGAAATGTGCGCGGAACCCCTATTTGTTTATTT<br>TTCTAAATACATTCAAATATGTATCCGCTCATGAGACAATAACCCTGATAAATGCTTCAATAATATTGAAA<br>AAGGAAGAGTATGAGTATTCAACATTTCCGTGTCGCCCTTATTCCCTTTTTTGCGGCATTTTGCCTTCCTG<br>TTTTTGCTCACCCAGAAACGCTGGTGAAAGTAAAAGATGCTGAAGATCAGTTGGGTGCACGAGTGGGTTAC<br>ATCGAACTGGATCTCAACAGCGGTAAGATCCTTGAGAGTTTTCGCCCCGAAGAACGTTTTCCAATGATGAG<br>CACTTTTAAAGTTCTGCTATGTGGCGCGGTATTATCCCGTATTGACGCCGGGCAAGAGCAACTCGGTCGCC<br>GCATACACTATTCTCAGAATGACTTGGTTGAGTACTCACCAGTCACAGAAAAGCATCTTACGGATGGCATG<br>ACAGTAAGAGAATTATGCAGTGCTGCCATAACCATGAGTGATAACACTGCGGCCAACTTACTTCTGACAAC<br>GATCGGAGGACCGAAGGAGCTAACCGCTTTTTTGCACAACATGGGGGATCATGTAACTCGCCTTGATCGTT<br>GGGAACCGGAGCTGAATGAAGCCATACCAAACGACGAGCGTGACACCACGATGCCTGTAGCAATGGCAACA<br>ACGTTGCGCAAACTATTAACTGGCGAACTACTTACTCTAGCTTCCCGGCAACAATTAATAGACTGGATGGA<br>GGCGGATAAAGTTGCAGGACCACTTCTGCGCTCGGCCCTTCCGGCTGGCTGGTTTATTGCTGATAAATCTG<br>GAGCCGGTGAGCGTGGGTCTCGCGGTATCATTGCAGCACTGGGGCCAGATGGTAAGCCCTCCCGTATCGTA<br>GTTATCTACACGACGGGGAGTCAGGCAACTATGGATGAACGAAATAGACAGATCGCTGAGATAGGTGCCTC<br>ACTGATTAAGCATTGGTAACTGTCAGACCAAGTTTACTCATATATACTTTAGATTGATTTAAAACTTCATT<br>TTTAATTTAAAAGGATCTAGGTGAAGATCCTTTTTGATAATCTCATGACCAAAATCCCTTAACGTGAGTTT<br>TCGTTCCACTGAGCGTCAGACCCCGTAGAAAAGATCAAAGGATCTTCTTGAGATCCTTTTTTTCTGCGCGT<br>AATCTGCTGCTTGCAAACAAAAAAACCACCGCTACCAGCGGTGGTTTGTTTGCCGGATCAAGAGCTACCAA<br>CTCTTTTTCCGAAGGTAACTGGCTTCAGCAGAGCGCAGATACCAAATACTGTTCTTCTAGTGTAGCCGTAG

	TTAGGCCACCACTTCAAGAACTCTGTAGCACCGCCTACATACCTCGCTCTGCTAATCCTGTTACCAGTGGC<br>TGCTGCCAGTGGCGATAAGTCGTGTCTTACCGGGTTGGACTCAAGACGATAGTTACCGGATAAGGCGCAGC<br>GGTCGGGCTGAACGGGGGGTTCGTGCACACAGCCCAGCTTGGAGCGAACGACCTACACCGAACTGAGATAC<br>CTACAGCGTGAGCTATGAGAAAGCGCCACGCTTCCCGAAGGGAGAAAGGCGGACAGGTATCCGGTAAGCGG<br>CAGGGTCGGAACAGGAGAGCGCACGAGGGAGCTTCCAGGGGGAAACGCCTGGTATCTTTATAGTCCTGTCG<br>GGTTTCGCCACCTCTGACTTGAGCGTCGATTTTTGTGATGCTCGTCAGGGGGGCGGAGCCTATGGAAAAAC<br>GCCAGCAACGCGGCCTTTTTCTTAAGC

**Plasmid**	**pDAC627**
**Description**	Expression of Csm complex from separate promoters; Puro backbone
**Utility**	RNA KD
**Features**	Pcmv-FLAG-NLS-Csm1-pA; Pcmv-FLAG-NLS-Csm2-pA; Pcmv-FLAG-NLS-Csm3-pA; Pcmv-FLAG-NLS-Csm4-pA; Pcmv-FLAG-NLS-Csm5-pA; Pcmv-FLAG-NLS-Cas6-pA; Pu6-crRNA-pT; Pcmv-Puro-pA
**Sequence**	GTGATGCGGTTTTGGCAGTACATCAATGGGCGTGGATAGCGGTTTGACTCACGGGGATTTCCAAGTCTCCA<br>CCCCATTGACGTCAATGGGAGTTTGTTTTGGCACCAAAATCAACGGGACTTTCCAAAATGTCGTAACAACT<br>CCGCCCCATTGACGCAAATGGGCGGTAGGCGTGTACGGTGGGAGGTCTATATAAGCAGAGCTCGTTTAGTG<br>AACCGTCAGATCTCTAGAgccgccaccATGCACCATCACCATCACCATTCCGGCGATTACAAAGACGATGA<br>CGATAAGATGGCGCCTAAGAAGAAACGCAAAGTGCGGGGCATGAAGAAAGAAAAGATTGATCTGTTTTACG<br>GAGCCCTGCTGCACGACATCGGAAAGGTCATCCAGCGAGCAACCGGAGAGCGGAAGAAACACGCACTTGTG<br>GGCGCCGACTGGTTCGACGAGATCGCCGACAACCAAGTCATCTCGGATCAGATCCGGTACCATATGGCCAA<br>CTACCAGTCTGATAAGCTCGGCAACGATCACCTGGCTTACATCACCTACATTGCCGACAACATCGCCTCCG<br>GTGTCGACCGCCGGCAATCCAACGAAGAGTCAGACGAAGATACCTCCGCAAAGATCTGGGACACCTACACG<br>AACCAGGCCGACATCTTTAACGTGTTCGGAGCGCAGACCGATAAGCGGTACTTCAAGCCTACCGTGCTGAA<br>TCTCAAGTCGAAGCCCAACTTCGCGTCCGCCACTTACGAACCCTTTAGCAAGGGCGATTACGCTGCCATCG<br>CCACCCGGATTAAGAACGAACTGGCCGAGTTCGAGTTCAACCAAGTCCAGATTGACTCCCTGCTCAACCTT<br>TTCGAGGCTACTCTCTCCTTCGTGCCGTCAAGCACCAACACTAAGGAAATCGCCGACATCTCCCTGGCCGA<br>CCATTCCCGCTTGACTGCTGCCTTCGCTCTGGCGATCTACGACTACCTGGAGGACAAGGGTCGGCACAACT<br>ACAAAGAGGACCTGTTCACCAAAGTGTCAGCGTTCTATGAAGAAGCCTTCCTGCTGGCCTCCTTCGAC<br>CTGTCGGGAATCCAGGACTTTATCTACAACATTAACATCGCAACTAACGGCGCGGCGAAGCAGCTGAAGGC<br>CCGGAGCCTCTACCTGGACTTTATGTCCGAGTACATCGCCGATAGCCTGCTGGACAAGCTGGGACTGAACA<br>GGGCTAACATGCTTTACGTCGGCGGCGGACACGCCTACTTCGTCCTGGCCAACACCGAAAAGACTGTGGAA<br>ACCCTGGTGCAGTTTGAGAAGGATTTCAACCAGTTCCTGTTGGCAAACTTCCAGACCCGCCTCTATGTGGC<br>CTTTGGCTGGGGTTCCTTCGCGGCCAAGGACATCATGTCCGAGCTGAATAGCCCCGAGTCCTACCGCCAAG<br>TGTACCAAAAGGCTTCGCGCATGATCTCCAAAAAGAAAATCTCCAGATACGACTACCAGACACTGATGCTC<br>CTGAATCGCGGTGGAAAGTCCTCAGAGAGAGAGTGCGAGATTTGCCACTCCGTGGAGAACCTGGTGTCCTA<br>CCACGACCAGAAAGTCTGTGACATTTGCCGGGGACTGTACCAGTTCTCGAAAGAAATTGCCCATGACCACT<br>TCATCATTACCGAAAATGAGGGGCTGCCGATTGGACCAAACGCGTGCTTAAAGGGCGTGGCATTCGAAAAG<br>CTGTCCCAAGAAGCGTTCAGCCGGGTCTACGTGAAGAATGACTATAAGGCCGGTACCGTGAAGGCTACGCA<br>TGTGTTCGTGGGGGATTACCAGTGCGACGAGATCTACAACTACGCCGCCCTGAGCAAGAACGAGAACGGCC<br>TAGGCATCAAGAGACTGGCCGTGGTCCGGCTCGACGTGGATGACTTGGGCGCCGCCTTCATGGCCGGTTTC<br>AGCCAGCAGGGAAACGGACAATACTCCACTCTGTCAAGATCGGCCACATTCTCCCGGAGCATGTCGCTGTT<br>CTTCAAAGTGTACATTAACCAGTTCGCCTCCGACAAGAAGCTGAGCATTATCTACGCGGGCGGCGATGACG<br>TGTTCGCCATTGGATCGTGGCAGGATATCATCGCGTTCACTGTGGAACTTCGCGAAAACTTCATCAAGTGG<br>ACCAACGGGAAGCTCACCCTCTCCGCGGGGATAGGGTTGTTCGCCGACAAGACTCCTATTAGCCTGATGGC<br>TCACCAGACCGGGGAACTGGAAGAGGCCGCCAAGGGCAACGAAAAGGACTCCATCTCGCTGTTCTCAAGCG<br>ACTACACTTTCAAGTTTGATAGGTTCATCACTAACGTGTACGACGACAAACTGGAACAGATTAGATACTTC<br>TTCAACCATCAAGACGAGAGGGGAAAGAACTTCATCTATAAGCTTATTGAGCTTTTGAGGAACCACGACCG<br>CATGAATATGGCACGCCTCGCCTATTACCTCACTCGCCTGGAAGAACTGACCCGGGAGACTGACAGGGACA<br>AGTTCAAGACCTTCAAGAACCTGTTCTACTCCTGGTACACCAACAAGAACGATAAGGACCGGAAGGAAGCC<br>GAGCTCGCGCTCCTGCTGTACATCTACGAAATCAGAAAGGATTAAcggcaataaaaagacagaataaaacg<br>cacggtgttgggtcgtttgttcGCACACATTAGCTAGCCGTCAGCACACATTGTGATGCGGTTTTGGCAGT<br>ACATCAATGGGCGTGGATAGCGGTTTGACTCACGGGGATTTCCAAGTCTCCACCCCATTGACGTCAATGGG<br>AGTTTGTTTTGGCACCAAAATCAACGGGACTTTCCAAAATGTCGTAACAACTCCGCCCCATTGACGCAAAT<br>GGGCGGTAGGCGTGTACGGTGGGAGGTCTATATAAGCAGAGCTCGTTTAGTGAACCGTCAGATCTCTAGAg<br>ccgccaccATGGATTACAAAGACGATGACGATAAGATGGCGCCTAAGAAGAAACGCAAAGTGCGGGGCATG<br>ACCATCCTGACCGACGAGAACTACGTGGACATCGCCGAGAAAGCCATCCTGAAGCTGGAAAGAAACACCAG<br>AAATAGAAAGAACCCTGATGCCTTCTTCCTGACCACATCTAAGCTGCGGAACCTGCTGAGCCTGACAAGCA<br>CCCTGTTCGACGAGAGCAAGGTGAAGGAATACGACGCCCTGCTGGACAGAATCGCTTATCTGAGAGTGCAG<br>TTCGTGTACCAGGCCGGCAGAGAGATCGCCGTGAAAGATCTGATCGAGAAGGCCCAGATCCTGGAAGCTCT<br>GAAAGAGATCAAGGACCGGGAAACCCTGCAGAGATTCTGCAGATACATGGAAGCCCTGGTGGCCTACTTCA<br>AGTTCTACGGCGGCAAGGACTGAcggcaataaaaagacagaataaaacgcacggtgttgggtcgtttgttc<br>AGTTCTTTTGCCTTACTTTCAATGCATGCGGTGATGCGGTTTTGGCAGTACATCAATGGGCGTGGATAGCG

	GTTTGACTCACGGGGATTTCCAAGTCTCCACCCCATTGACGTCAATGGGAGTTTGTTTTGGCACCAAAATC<br>AACGGGACTTTCCAAAATGTCGTAACAACTCCGCCCCATTGACGCAAATGGGCGGTAGGCGTGTACGGTGG<br>GAGGTCTATATAAGCAGAGCTCGTTTAGTGAACCGTCAGATCTCTAGAgccgccaccATGGATTACAAAGA<br>CGATGACGATAAGATGGCGCCTAAGAAGAAACGCAAAGTGCGGGGCATGACCTTCGCCAAGATCAAATTCA<br>GCGCCCAGATCCGGCTGGAAACCGGCCTGCACATCGGAGGATCTGATGCCTTTGCCGCTATCGGCGCCATC<br>GACAGCCCTGTGATCAAGGACCCCATCACCAACCTGCCTATCATCCCCGGCTCTAGCCTGAAGGGCAAGAT<br>GAGAACACTGCTGGCCAAGGTGTACAACGAAAAGGTGGCCGAGAAGCCTAGCGACGACAGCGACATCCTGA<br>GCAGACTGTTCGGAAATAGCAAGGATAAGCGGTTCAAGATGGGCAGACTGATCTTCCGGGACGCCTTCCTG<br>AGCAACGCCGACGAGCTGGATTCTCTGGGCGTGCGGAGCTACACCGAGGTGAAGTTCGAGAACACCATCGA<br>TAGAATCACCGCCGAGGCCAATCCTAGACAGATCGAGAGAGCCATTCGGAACTCAACATTCGACTTCGAGC<br>TGATCTACGAGATCACTGATGAGAATGAGAACCAGGTCGAGGAAGATTTCAAGGTGATCAGAGACGGCCTG<br>AAGCTGCTGGAACTGGACTACCTGGGCGGAAGCGGCTCCAGAGGCTACGGCAAAGTGGCTTTTGAGAACCT<br>GAAAGCCACCACAGTGTTCGGCAACTACGACGTGAAAACCCTGAACGAGCTGCTGACCGCCGAAGTGTGAc<br>ggcaataaaaagacagaataaaacgcacggtgttgggtcgtttgttcTGGATTGCGAGAATGGACTAGTAG<br>CAAACTGTGATGCGGTTTTGGCAGTACATCAATGGGCGTGGATAGCGGTTTGACTCACGGGGATTTCCAAG<br>TCTCCACCCCATTGACGTCAATGGGAGTTTGTTTTGGCACCAAAATCAACGGGACTTTCCAAAATGTCGTA<br>ACAACTCCGCCCCATTGACGCAAATGGGCGGTAGGCGTGTACGGTGGGAGGTCTATATAAGCAGAGCTCGT<br>TTAGTGAACCGTCAGATCTCTAGAgccgccaccATGGATTACAAAGACGATGACGATAAGATGGCGCCTAA<br>GAAGAAACGCAAAGTGCGGGGCATGACTTACAAGCTCTACATTATGACCTTTCAAAACGCCCACTTCGGTT<br>CCGGCACTCTGGACTCATCGAAGCTGACCTTCTCCGCGGATAGAATCTTCTCGGCACTCGTGCTCGAGGCT<br>CTGAAGATGGGAAAGCTCGACGCCTTCTTGGCCGAGGCCAACCAGGATAAGTTCACTCTGACCGACGCGTT<br>CCCATTCCAATTCGGTCCTTTCCTGCCGAAACCGATTGGTTACCCCAAGCACGACCAGATCGACCAGTCTG<br>TGGACGTGAAGGAAGTCCGCCGCCAAGCGAAGCTGTCCAAAAAGCTCCAGTTCCTGGCTCTGGAAAACGTC<br>GACGACTACCTGAACGGAGAGCTGTTTGAGAATGAGGAACACGCCGTGATCGACACAGTGACCAAGAACCA<br>GCCCCATAAAGATGATAATCTGTACCAAGTGGCCACCACTCGGTTCTCGAACGACACCTCCCTTTACGTGA<br>TCGCCAACGAATCCGATCTGCTGAACGAACTGATGAGCAGCCTTCAGTACTCCGGGCTGGGCGGCAAAAGG<br>TCCTCAGGATTCGGCAGATTTGAGCTGGACATCCAGAACATTCCCTTGGAACTGTCCGACCGGCTGACGAA<br>GAACCACAGCGACAAGGTCATGTCACTTACCACCGCCCTCCCGGTGGACGCTGATCTCGAGGAAGCGATGG<br>AAGATGGCCATTACCTGTTGACCAAGTCGTCCGGATTCGCATTCTCCCACGCCACCAACGAAAACTATCGG<br>AAGCAGGACCTGTACAAGTTCGCCTCCGGGAGCACCTTCAGCAAGACTTTCGAGGGACAGATCGTGGACGT<br>GCGCCCTCTCGATTTCCCTCACGCCGTGCTGAACTACGCCAAGCCGCTGTTCTTTAAGCTCGAAGTCTAAc<br>ggcaataaaaagacagaataaaacgcacggtgttgggtcgtttgttcGCACATTCAAAAACAGGCAATTGG<br>ACAAGCGTGATGCGGTTTTGGCAGTACATCAATGGGCGTGGATAGCGGTTTGACTCACGGGGATTTCCAAG<br>TCTCCACCCCATTGACGTCAATGGGAGTTTGTTTTGGCACCAAAATCAACGGGACTTTCCAAAATGTCGTA<br>ACAACTCCGCCCCATTGACGCAAATGGGCGGTAGGCGTGTACGGTGGGAGGTCTATATAAGCAGAGCTCGT<br>TTAGTGAACCGTCAGATCTCTAGAgccgccaccATGGATTACAAAGACGATGACGATAAGATGGCGCCTAA<br>GAAGAAACGCAAAGTGCGGGGCATGAAAAATGACTACCGGACCTTCAAGCTGAGCCTGCTGACCCTGGCTC<br>CTATCCACATCGGCAACGGCGAGAAGTACACCAGCAGAGAATTCATCTACGAGAACAAGAAGTTCTACTTC<br>CCCGACATGGGCAAGTTCTACAACAAGATGGTGGAAAAGAGACTGGCCGAGAAGTTCGAGGCCTTCCTGAT<br>CCAGACCAGACCCAACGCCAGAAACAACCGGCTGATTTCTTTTCTGAACGACAACAGAATCGCCGAAAGAT<br>CTTTTGGCGGCTACAGCATCAGTGAAACCGGCCTGGAATCTGATAAGAACCCTAACAGCGCCGGAGCTATC<br>AACGAGGTGAACAAATTCATCCGGGACGCCTTCGGAAATCCTTACATCCCAGGCAGCAGCCTGAAGGGCGC<br>CATCCGCACCATCCTGATGAACACCACACCTAAGTGGAACAACGAGAACGCCGTGAACGACTTCGGCAGAT<br>TCCCAAAGGAAAACAAGAACCTGATCCCTTGGGGACCTAAGAAAGGCAAGGAATACGACGACCTGTTCAAC<br>GCCATCAGAGTGTCCGACAGCAAGCCCTTCGACAACAAAAGCCTGATCCTCGTGCAGAAGTGGGACTACAG<br>CGCCAAAACCAACAAGGCCAAGCCTCTGCCTCTGTACAGAGAGTCTATCAGCCCTCTGACCAAGATCGAGT<br>TCGAGATAACAACAACCACTGATGAGGCCGGCAGACTGATCGAGGAACTGGGAAAGCGGGCCCAGGCCTTT<br>TATAAGGACTACAAGGCCTTTTTCCTGTCTGAATTCCCTGATGATAAGATCCAGGCTAATCTGCAATACCC<br>CATCTACCTGGGCGCCGGCAGCGGCGCTTGGACAAAGACCCTGTTTAAGCAGGCCGACGGCATCCTGCAGC<br>GGAGATACTCCAGAATGAAAACCAAGATGGTCAAGAAGGGCGTGCTGAAGCTGACAAAGGCCCCTCTGAAA<br>ACAGTGAAGATCCCCAGCGGCAACCACAGCCTGGTGAAGAATCACGAGAGCTTCTACGAGATGGGCAAAGC<br>CAACTTCATGATCAAGGAAATCGACAAGTGAcggcaataaaaagacagaataaaacgcacggtgttgggtc<br>gtttgttcATGTGTGCCGGCCGGAGAATAAAAGTCTAAGTGATGCGGTTTTGGCAGTACATCAATGGGCGT<br>GGATAGCGGTTTGACTCACGGGGATTTCCAAGTCTCCACCCCATTGACGTCAATGGGAGTTTGTTTTGGCA<br>CCAAAATCAACGGGACTTTCCAAAATGTCGTAACAACTCCGCCCCATTGACGCAAATGGGCGGTAGGCGTG<br>TACGGTGGGAGGTCTATATAAGCAGAGCTCGTTTAGTGAACCGTCAGATCTCTAGAgccgccaccATGGAT<br>TACAAAGACGATGACGATAAGATGGCGCCTAAGAAGAAACGCAAAGTGCGGGGCATGAAAAAGCTCGTGTT<br>CACCTTTAAGCGGATCGACCACCCTGCTCAGGACCTGGCCGTGAAATTCCACGGCTTCCTGATGGAACAGC<br>TGGATAGCGACTACGTGGACTACCTGCACCAGCAGCAGACCAACCCCTACGCCACAAAGGTGATCCAGGGC<br>AAAGAGAACACCCAGTGGGTCGTGCATCTGCTGACAGACGACATCGAGGACAAGGTGTTCATGACCCTGCT<br>GCAGATCAAGGAAGTGTCCCTGAACGACCTGCCTAAGTTGTCTGTGGAAAAGGTGGAAATCCAGGAGCTGG<br>GCGCTGATAAGCTGCTCGAGATCTTCAACAGCGAGGAAAACCAGACCTACTTCAGCATCATCTTCGAGACA<br>CCTACAGGCTTTAAAAGCCAGGGCAGCTACGTGATCTTCCCCAGCATGCGGCTGATCTTTCAGAGCCTGAT

	GCAGAAGTACGGCAGACTGGTGGAAAACCAGCCTGAGATCGAGGAAGATACCCTGGACTACCTGAGCGAGC<br>ACAGCACCATCACCAATTACAGACTGGAAACAAGCTACTTCAGAGTGCATAGACAGAGAATCCCCGCCTTC<br>CGGGGCAAGCTGACCTTCAAGGTGCAGGGAGCCCAGACACTGAAGGCCTACGTGAAGATGCTGCTGACCTT<br>CGGCGAGTACAGCGGCCTGGGCATGAAAACCAGCCTGGGAATGGGCGGCATCAAGCTGGAAGAAAGAAAGG<br>ACTGAcggcaataaaaagacagaataaaacgcacggtgttgggtcgtttgttcGTGCGATAGAGGGATCCC<br>GCATTGAATTATGTGATGCGGTTTTGGCAGTACATCAATGGGCGTGGATAGCGGTTTGACTCACGGGGATT<br>TCCAAGTCTCCACCCCATTGACGTCAATGGGAGTTTGTTTTGGCACCAAAATCAACGGGACTTTCCAAAAT<br>GTCGTAACAACTCCGCCCCATTGACGCAAATGGGCGGTAGGCGTGTACGGTGGGAGGTCTATATAAGCAGA<br>GCTCGTTTAGTGAACCGTCAGATCTCTAGAgccgccaccATGACCGAGTACAAGCCCACGGTGCGCCTCGC<br>CACCCGCGACGACGTCCCCAGGGCCGTACGCACCCTCGCCGCCGCGTTCGCCGACTACCCCGCCACGCGCC<br>ACACCGTCGATCCGGACCGCCACATCGAGCGGGTCACCGAGCTGCAAGAACTCTTCCTCACGCGCGTCGGG<br>CTCGACATCGGCAAGGTGTGGGTCGCGGACGACGGCGCCGCGGTGGCGGTCTGGACCACGCCGGAGAGCGT<br>CGAAGCGGGGGCGGTGTTCGCCGAGATCGGCCCGCGCATGGCCGAGTTGAGCGGTTCCCGGCTGGCCGCGC<br>AGCAACAGATGGAAGGCCTCCTGGCGCCGCACCGGCCCAAGGAGCCCGCGTGGTTCCTGGCCACCGTCGGA<br>GTCTCGCCCGACCACCAGGGCAAGGGTCTGGGCAGCGCCGTCGTGCTCCCCGGAGTGGAGGCGGCGGAGCG<br>CGCCGGGGTGCCCGCCTTCCTGGAAACCTCCGCGCCCCGCAACCTCCCCTTCTACGAGCGGCTCGGCTTCA<br>CCGTCACCGCCGACGTCGAGGTGCCCGAAGGACCGCGCACCTGGTGCATGACCCGCAAGCCCGGTGCCTGA<br>cggcaataaaaagacagaataaaacgcacggtgttgggtcgtttgttcGAGCAGATTGTACTGAGAGTGCA<br>CCGGTTGGAGGGCCTATTTCCCATGATTCCTTCATATTTGCATATACGATACAAGGCTGTTAGAGAGATAA<br>TTAGAATTAATTTGACTGTAAACACAAAGATATTAGTACAAAATACGTGACGTAGAAAGTAATAATTTCTT<br>GGGTAGTTTGCAGTTTTAAAATTATGTTTTAAAATGGACTATCATATGCTTACCGTAACTTGAAAGTATTT<br>CGATTTCTTGGCTTTATATATCTTGTGGAAAGGACGAAACACCGATATAAACCTAATTACCTCGAGAGGGG<br>ACGGAAACCCGTCTTCGATGAAGCGATTCAGAAGACTTGATATAAACCTAATTACCTCGAGAGGGGACTTT<br>TTTACATGTGTCAGAGGTTTTCACCGTCATCACCGAAACGCGCGAGACGAAAGGGCCTCGTGATACGCCTA<br>TTTTTATAGGTTAATGTCATGATAATAATGGTTTCTTAGACGTCAGGTGGCACTTTTCGGGGAAATGTGCG<br>CGGAACCCCTATTTGTTTATTTTTCTAAATACATTCAAATATGTATCCGCTCATGAGACAATAACCCTGAT<br>AAATGCTTCAATAATATTGAAAAAGGAAGAGTATGAGTATTCAACATTTCCGTGTCGCCCTTATTCCCTTT<br>TTTGCGGCATTTTGCCTTCCTGTTTTTGCTCACCCAGAAACGCTGGTGAAAGTAAAAGATGCTGAAGATCA<br>GTTGGGTGCACGAGTGGGTTACATCGAACTGGATCTCAACAGCGGTAAGATCCTTGAGAGTTTTCGCCCCG<br>AAGAACGTTTTCCAATGATGAGCACTTTTAAAGTTCTGCTATGTGGCGCGGTATTATCCCGTATTGACGCC<br>GGGCAAGAGCAACTCGGTCGCCGCATACACTATTCTCAGAATGACTTGGTTGAGTACTCACCAGTCACAGA<br>AAAGCATCTTACGGATGGCATGACAGTAAGAGAATTATGCAGTGCTGCCATAACCATGAGTGATAACACTG<br>CGGCCAACTTACTTCTGACAACGATCGGAGGACCGAAGGAGCTAACCGCTTTTTTGCACAACATGGGGGAT<br>CATGTAACTCGCCTTGATCGTTGGGAACCGGAGCTGAATGAAGCCATACCAAACGACGAGCGTGACACCAC<br>GATGCCTGTAGCAATGGCAACAACGTTGCGCAAACTATTAACTGGCGAACTACTTACTCTAGCTTCCCGGC<br>AACAATTAATAGACTGGATGGAGGCGGATAAAGTTGCAGGACCACTTCTGCGCTCGGCCCTTCCGGCTGGC<br>TGGTTTATTGCTGATAAATCTGGAGCCGGTGAGCGTGGGTCTCGCGGTATCATTGCAGCACTGGGGCCAGA<br>TGGTAAGCCCTCCCGTATCGTAGTTATCTACACGACGGGGAGTCAGGCAACTATGGATGAACGAAATAGAC<br>AGATCGCTGAGATAGGTGCCTCACTGATTAAGCATTGGTAACTGTCAGACCAAGTTTACTCATATATACTT<br>TAGATTGATTTAAAACTTCATTTTTAATTTAAAAGGATCTAGGTGAAGATCCTTTTTGATAATCTCATGAC<br>CAAAATCCCTTAACGTGAGTTTTCGTTCCACTGAGCGTCAGACCCCGTAGAAAAGATCAAAGGATCTTCTT<br>GAGATCCTTTTTTTCTGCGCGTAATCTGCTGCTTGCAAACAAAAAAACCACCGCTACCAGCGGTGGTTTGT<br>TTGCCGGATCAAGAGCTACCAACTCTTTTTCCGAAGGTAACTGGCTTCAGCAGAGCGCAGATACCAAATAC<br>TGTTCTTCTAGTGTAGCCGTAGTTAGGCCACCACTTCAAGAACTCTGTAGCACCGCCTACATACCTCGCTC<br>TGCTAATCCTGTTACCAGTGGCTGCTGCCAGTGGCGATAAGTCGTGTCTTACCGGGTTGGACTCAAGACGA<br>TAGTTACCGGATAAGGCGCAGCGGTCGGGCTGAACGGGGGGTTCGTGCACACAGCCCAGCTTGGAGCGAAC<br>GACCTACACCGAACTGAGATACCTACAGCGTGAGCTATGAGAAAGCGCCACGCTTCCCGAAGGGAGAAAGG<br>CGGACAGGTATCCGGTAAGCGGCAGGGTCGGAACAGGAGAGCGCACGAGGGAGCTTCCAGGGGGAAACGCC<br>TGGTATCTTTATAGTCCTGTCGGGTTTCGCCACCTCTGACTTGAGCGTCGATTTTTGTGATGCTCGTCAGG<br>GGGGCGGAGCCTATGGAAAAACGCCAGCAACGCGGCCTTTTTCTTAAGC

**Plasmid**	**pDAC569**
**Description**	Expression of Csm complex (RNase mut) from separate promoters; GFP backbone
**Utility**	RNA binding/tethering/pulldown
**Features**	Pcmv-FLAG-NLS-Csm1-pA; Pcmv-FLAG-NLS-Csm2-pA; Pcmv-FLAG-NLS-Csm3(RNase mut)-pA; Pcmv-FLAG-NLS-Csm4-pA; Pcmv-FLAG-NLS-Csm5-pA; Pcmv-FLAG-NLS-Cas6-pA; Pu6-crRNA-pT; Pcmv-GFP-pA
**Sequence**	GTGATGCGGTTTTGGCAGTACATCAATGGGCGTGGATAGCGGTTTGACTCACGGGGATTTCCAAGTCTCCA<br>CCCCATTGACGTCAATGGGAGTTTGTTTTGGCACCAAAATCAACGGGACTTTCCAAAATGTCGTAACAACT<br>CCGCCCCATTGACGCAAATGGGCGGTAGGCGTGTACGGTGGGAGGTCTATATAAGCAGAGCTCGTTTAGTG<br>AACCGTCAGATCTCTAGAgccgccaccATGCACCATCACCATCACCATTCCGGCGATTACAAAGACGATGA<br>CGATAAGATGGCGCCTAAGAAGAAACGCAAAGTGCGGGGCATGAAGAAAGAAAAGATTGATCTGTTTTACG

GAGCCCTGCTGCACGACATCGGAAAGGTCATCCAGCGAGCAACCGGAGAGCGGAAGAAACACGCACTTGTG
GGCGCCGACTGGTTCGACGAGATCGCCGACAACCAAGTCATCTCGGATCAGATCCGGTACCATATGGCCAA
CTACCAGTCTGATAAGCTCGGCAACGATCACCTGGCTTACATCACCTACATTGCCGACAACATCGCCTCCG
GTGTCGACCGCCGGCAATCCAACGAAGAGTCAGACGAAGATACCTCCGCAAAGATCTGGGACACCTACACG
AACCAGGCCGACATCTTTAACGTGTTCGGAGCGCAGACCGATAAGCGGTACTTCAAGCCTACCGTGCTGAA
TCTCAAGTCGAAGCCCAACTTCGCGTCCGCCACTTACGAACCCTTTAGCAAGGGCGATTACGCTGCCATCG
CCACCCGGATTAAGAACGAACTGGCCGAGTTCGAGTTCAACCAAGTCCAGATTGACTCCCTGCTCAACCTT
TTCGAGGCTACTCTCTCCTTCGTGCCGTCAAGCACCAACACTAAGGAAATCGCCGACATCTCCCTGGCCGA
CCATTCCCGCTTGACTGCTGCCTTCGCTCTGGCGATCTACGACTACCTGGAGGACAAGGGTCGGCACAACT
ACAAAGAGGACCTGTTCACCAAAGTGTCAGCGTTCTATGAAGAAGAAGCCTTCCTGCTGGCCTCCTTCGAC
CTGTCGGGAATCCAGGACTTTATCTACAACATTAACATCGCAACTAACGGCGCGGCGAAGCAGCTGAAGGC
CCGGAGCCTCTACCTGGACTTTATGTCCGAGTACATCGCCGATAGCCTGCTGGACAAGCTGGGACTGAACA
GGGCTAACATGCTTTACGTCGGCGGCGGACACGCCTACTTCGTCCTGGCCAACACCGAAAAGACTGTGGAA
ACCCTGGTGCAGTTTGAGAAGGATTTCAACCAGTTCCTGTTGGCAAACTTCCAGACCCGCCTCTATGTGGC
CTTTGGCTGGGGTTCCTTCGCGGCCAAGGACATCATGTCCGAGCTGAATAGCCCCGAGTCCTACCGCCAAG
TGTACCAAAAGGCTTCGCGCATGATCTCCAAAAAGAAAATCTCCAGATACGACTACCAGACACTGATGCTC
CTGAATCGCGGTGGAAAGTCCTCAGAGAGAGAGTGCGAGATTTGCCACTCCGTGGAGAACCTGGTGTCCTA
CCACGACCAGAAAGTCTGTGACATTTGCCGGGGACTGTACCAGTTCTCGAAAGAAATTGCCCATGACCACT
TCATCATTACCGAAAATGAGGGGCTGCCGATTGGACCAAACGCGTGCTTAAAGGGCGTGGCATTCGAAAAG
CTGTCCCAAGAAGCGTTCAGCCGGGTCTACGTGAAGAATGACTATAAGGCCGGTACCGTGAAGGCTACGCA
TGTGTTCGTGGGGGATTACCAGTGCGACGAGATCTACAACTACGCCGCCCTGAGCAAGAACGAGAACGGCC
TAGGCATCAAGAGACTGGCCGTGGTCCGGCTCGACGTGGATGACTTGGGCGCCGCCTTCATGGCCGGTTTC
AGCCAGCAGGGAAACGGACAATACTCCACTCTGTCAAGATCGGCCACATTCTCCCGGAGCATGTCGCTGTT
CTTCAAAGTGTACATTAACCAGTTCGCCTCCGACAAGAAGCTGAGCATTATCTACGCGGGCGGCGATGACG
TGTTCGCCATTGGATCGTGGCAGGATATCATCGCGTTCACTGTGGAACTTCGCGAAAACTTCATCAAGTGG
ACCAACGGGAAGCTCACCCTCTCCGCGGGGATAGGGTTGTTCGCCGACAAGACTCCTATTAGCCTGATGGC
TCACCAGACCGGGGAACTGGAAGAGGCCGCCAAGGGCAACGAAAAGGACTCCATCTCGCTGTTCTCAAGCG
ACTACACTTTCAAGTTTGATAGGTTCATCACTAACGTGTACGACGACAAACTGGAACAGATTAGATACTTC
TTCAACCATCAAGACGAGAGGGGAAAGAACTTCATCTATAAGCTTATTGAGCTTTTGAGGAACCACGACCG
CATGAATATGGCACGCCTCGCCTATTACCTCACTCGCCTGGAAGAACTGACCCGGGAGACTGACAGGGACA
AGTTCAAGACCTTCAAGAACCTGTTCTACTCCTGGTACACCAACAAGAACGATAAGGACCGGAAGGAAGCC
GAGCTCGCGCTCCTGCTGTACATCTACGAAATCAGAAAGGATTAAcggcaataaaaagacagaataaaacg
cacggtgttgggtcgtttgttcGCACACATTAGCTAGCCGTCAGCACACATTGTGATGCGGTTTTGGCAGT
ACATCAATGGGCGTGGATAGCGGTTTGACTCACGGGGATTTCCAAGTCTCCACCCCATTGACGTCAATGGG
AGTTTGTTTTGGCACCAAAATCAACGGGACTTTCCAAAATGTCGTAACAACTCCGCCCCATTGACGCAAAT
GGGCGGTAGGCGTGTACGGTGGGAGGTCTATATAAGCAGAGCTCGTTTAGTGAACCGTCAGATCTCTAGAg
ccgccaccATGGATTACAAAGACGATGACGATAAGATGGCGCCTAAGAAGAAACGCAAAGTGCGGGGCATG
ACCATCCTGACCGACGAGAACTACGTGGACATCGCCGAGAAAGCCATCCTGAAGCTGGAAAGAAACACCAG
AAATAGAAAGAACCCTGATGCCTTCTTCCTGACCACATCTAAGCTGCGGAACCTGCTGAGCCTGACAAGCA
CCCTGTTCGACGAGAGCAAGGTGAAGGAATACGACGCCCTGCTGGACAGAATCGCTTATCTGAGAGTGCAG
TTCGTGTACCAGGCCGGCAGAGAGATCGCCGTGAAAGATCTGATCGAGAAGGCCCAGATCCTGGAAGCTCT
GAAAGAGATCAAGGACCGGGAAACCCTGCAGAGATTCTGCAGATACATGGAAGCCCTGGTGGCCTACTTCA
AGTTCTACGGCGGCAAGGACTGAcggcaataaaaagacagaataaaacgcacggtgttgggtcgtttgttc
AGTTCTTTTGCCTTACTTTCAATGCATGCGGTGATGCGGTTTTGGCAGTACATCAATGGGCGTGGATAGCG
GTTTGACTCACGGGGATTTCCAAGTCTCCACCCCATTGACGTCAATGGGAGTTTGTTTTGGCACCAAAATC
AACGGGACTTTCCAAAATGTCGTAACAACTCCGCCCCATTGACGCAAATGGGCGGTAGGCGTGTACGGTGG
GAGGTCTATATAAGCAGAGCTCGTTTAGTGAACCGTCAGATCTCTAGAgccgccaccATGGATTACAAAGA
CGATGACGATAAGATGGCGCCTAAGAAGAAACGCAAAGTGCGGGGCATGACCTTCGCCAAGATCAAATTCA
GCGCCCAGATCCGGCTGGAAACCGGCCTGCACATCGGAGGATCTGATGCCTTTGCCGCTATCGGCGCCATC
GCCAGCCCTGTGATCAAGGACCCCATCACCAACCTGCCTATCATCCCCGGCTCTAGCCTGAAGGGCAAGAT
GAGAACACTGCTGGCCAAGGTGTACAACGAAAAGGTGGCCGAGAAGCCTAGCGACGACAGCGACATCCTGA
GCAGACTGTTCGGAAATAGCAAGGATAAGCGGTTCAAGATGGGCAGACTGATCTTCCGGGACGCCTTCCTG
AGCAACGCCGACGAGCTGGATTCTCTGGGCGTGCGGAGCTACACCGAGGTGAAGTTCGAGAACACCATCGA
TAGAATCACCGCCGAGGCCAATCCTAGACAGATCGAGAGAGCCATTCGGAACTCAACATTCGACTTCGAGC
TGATCTACGAGATCACTGATGAGAATGAGAACCAGGTCGAGGAAGATTTCAAGGTGATCAGAGACGGCCTG
AAGCTGCTGGAACTGGACTACCTGGGCGGAAGCGGCTCCAGAGGCTACGGCAAAGTGGCTTTTGAGAACCT
GAAAGCCACCACAGTGTTCGGCAACTACGACGTGAAAACCCTGAACGAGCTGCTGACCGCCGAAGTGTGAc
ggcaataaaaagacagaataaaacgcacggtgttgggtcgtttgttcTGGATTGCGAGAATGGACTAGTAG
CAAACTGTGATGCGGTTTTGGCAGTACATCAATGGGCGTGGATAGCGGTTTGACTCACGGGGATTTCCAAG
TCTCCACCCCATTGACGTCAATGGGAGTTTGTTTTGGCACCAAAATCAACGGGACTTTCCAAAATGTCGTA
ACAACTCCGCCCCATTGACGCAAATGGGCGGTAGGCGTGTACGGTGGGAGGTCTATATAAGCAGAGCTCGT
TTAGTGAACCGTCAGATCTCTAGAgccgccaccATGGATTACAAAGACGATGACGATAAGATGGCGCCTAA
GAAGAAACGCAAAGTGCGGGGCATGACTTACAAGCTCTACATTATGACCTTTCAAAACGCCCACTTCGGTT

	CCGGCACTCTGGACTCATCGAAGCTGACCTTCTCCGCGGATAGAATCTTCTCGGCACTCGTGCTCGAGGCT<br>CTGAAGATGGGAAAGCTCGACGCCTTCTTGGCCGAGGCCAACCAGGATAAGTTCACTCTGACCGACGCGTT<br>CCCATTCCAATTCGGTCCTTTCCTGCCGAAACCGATTGGTTACCCCAAGCACGACCAGATCGACCAGTCTG<br>TGGACGTGAAGGAAGTCCGCCGCCAAGCGAAGCTGTCCAAAAAGCTCCAGTTCCTGGCTCTGGAAAACGTC<br>GACGACTACCTGAACGGAGAGCTGTTTGAGAATGAGGAACACGCCGTGATCGACACAGTGACCAAGAACCA<br>GCCCCATAAAGATGATAATCTGTACCAAGTGGCCACCACTCGGTTCTCGAACGACACCTCCCTTTACGTGA<br>TCGCCAACGAATCCGATCTGCTGAACGAACTGATGAGCAGCCTTCAGTACTCCGGGCTGGGCGGCAAAAGG<br>TCCTCAGGATTCGGCAGATTTGAGCTGGACATCCAGAACATTCCCTTGGAACTGTCCGACCGGCTGACGAA<br>GAACCACAGCGACAAGGTCATGTCACTTACCACCGCCCTCCCGGTGGACGCTGATCTCGAGGAAGCGATGG<br>AAGATGGCCATTACCTGTTGACCAAGTCGTCCGGATTCGCATTCTCCCACGCCACCAACGAAAACTATCGG<br>AAGCAGGACCTGTACAAGTTCGCCTCCGGGAGCACCTTCAGCAAGACTTTCGAGGGACAGATCGTGGACGT<br>GCGCCCTCTCGATTTCCCTCACGCCGTGCTGAACTACGCCAAGCCGCTGTTCTTTAAGCTCGAAGTCTAAc<br>ggcaataaaaagacagaataaaacgcacggtgttgggtcgtttgttcGCACATTCAAAAACAGGCAATTGG<br>ACAAGCGTGATGCGGTTTTGGCAGTACATCAATGGGCGTGGATAGCGGTTTGACTCACGGGGATTTCCAAG<br>TCTCCACCCCATTGACGTCAATGGGAGTTTGTTTTGGCACCAAAATCAACGGGACTTTCCAAAATGTCGTA<br>ACAACTCCGCCCCATTGACGCAAATGGGCGGTAGGCGTGTACGGTGGGAGGTCTATATAAGCAGAGCTCGT<br>TTAGTGAACCGTCAGATCTCTAGAgccgccaccATGGATTACAAAGACGATGACGATAAGATGGCGCCTAA<br>GAAGAAACGCAAAGTGCGGGGCATGAAAAATGACTACCGGACCTTCAAGCTGAGCCTGCTGACCCTGGCTC<br>CTATCCACATCGGCAACGGCGAGAAGTACACCAGCAGAGAATTCATCTACGAGAACAAGAAGTTCTACTTC<br>CCCGACATGGGCAAGTTCTACAACAAGATGGTGGAAAAGAGACTGGCCGAGAAGTTCGAGGCCTTCCTGAT<br>CCAGACCAGACCCAACGCCAGAAACAACCGGCTGATTTCTTTTCTGAACGACAACAGAATCGCCGAAAGAT<br>CTTTTGGCGGCTACAGCATCAGTGAAACCGGCCTGGAATCTGATAAGAACCCTAACAGCGCCGGAGCTATC<br>AACGAGGTGAACAAATTCATCCGGGACGCCTTCGGAAATCCTTACATCCCAGGCAGCAGCCTGAAGGGCGC<br>CATCCGCACCATCCTGATGAACACCACACCTAAGTGGAACAACGAGAACGCCGTGAACGACTTCGGCAGAT<br>TCCCAAAGGAAAACAAGAACCTGATCCCTTGGGGACCTAAGAAAGGCAAGGAATACGACGACCTGTTCAAC<br>GCCATCAGAGTGTCCGACAGCAAGCCCTTCGACAACAAAAGCCTGATCCTCGTGCAGAAGTGGGACTACAG<br>CGCCAAAACCAACAAGGCCAAGCCTCTGCCTCTGTACAGAGAGTCTATCAGCCCTCTGACCAAGATCGAGT<br>TCGAGATAACAACAACCACTGATGAGGCCGGCAGACTGATCGAGGAACTGGGAAAGCGGGCCCAGGCCTTT<br>TATAAGGACTACAAGGCCTTTTTCCTGTCTGAATTCCCTGATGATAAGATCCAGGCTAATCTGCAATACCC<br>CATCTACCTGGGCGCCGGCAGCGGCGCTTGGACAAAGACCCTGTTTAAGCAGGCCGACGGCATCCTGCAGC<br>GGAGATACTCCAGAATGAAAACCAAGATGGTCAAGAAGGGCGTGCTGAAGCTGACAAAGGCCCCTCTGAAA<br>ACAGTGAAGATCCCCAGCGGCAACCACAGCCTGGTGAAGAATCACGAGAGCTTCTACGAGATGGGCAAAGC<br>CAACTTCATGATCAAGGAAATCGACAAGTGAcggcaataaaaagacagaataaaacgcacggtgttgggtc<br>gtttgttcATGTGTGCCGGCCGGAGAATAAAAGTCTAAGTGATGCGGTTTTGGCAGTACATCAATGGGCGT<br>GGATAGCGGTTTGACTCACGGGGATTTCCAAGTCTCCACCCCATTGACGTCAATGGGAGTTTGTTTTGGCA<br>CCAAAATCAACGGGACTTTCCAAAATGTCGTAACAACTCCGCCCCATTGACGCAAATGGGCGGTAGGCGTG<br>TACGGTGGGAGGTCTATATAAGCAGAGCTCGTTTAGTGAACCGTCAGATCTCTAGAgccgccaccATGGAT<br>TACAAAGACGATGACGATAAGATGGCGCCTAAGAAGAAACGCAAAGTGCGGGGCATGAAAAAGCTCGTGTT<br>CACCTTTAAGCGGATCGACCACCCTGCTCAGGACCTGGCCGTGAAATTCCACGGCTTCCTGATGGAACAGC<br>TGGATAGCGACTACGTGGACTACCTGCACCAGCAGCAGACCAACCCCTACGCCACAAAGGTGATCCAGGGC<br>AAAGAGAACACCCAGTGGGTCGTGCATCTGCTGACAGACGACATCGAGGACAAGGTGTTCATGACCCTGCT<br>GCAGATCAAGGAAGTGTCCCTGAACGACCTGCCTAAGTTGTCTGTGGAAAAGGTGGAAATCCAGGAGCTGG<br>GCGCTGATAAGCTGCTCGAGATCTTCAACAGCGAGGAAAACCAGACCTACTTCAGCATCATCTTCGAGACA<br>CCTACAGGCTTTAAAAGCCAGGGCAGCTACGTGATCTTCCCCAGCATGCGGCTGATCTTTCAGAGCCTGAT<br>GCAGAAGTACGGCAGACTGGTGGAAAACCAGCCTGAGATCGAGGAAGATACCCTGGACTACCTGAGCGAGC<br>ACAGCACCATCACCAATTACAGACTGGAAACAAGCTACTTCAGAGTGCATAGACAGAGAATCCCCGCCTTC<br>CGGGGCAAGCTGACCTTCAAGGTGCAGGGAGCCCAGACACTGAAGGCCTACGTGAAGATGCTGCTGACCTT<br>CGGCGAGTACAGCGGCCTGGGCATGAAAACCAGCCTGGGAATGGGCGGCATCAAGCTGGAAGAAAGAAAGG<br>ACTGAcggcaataaaaagacagaataaaacgcacggtgttgggtcgtttgttcGTGCGATAGAGGGATCCC<br>GCATTGAATTATGTGATGCGGTTTTGGCAGTACATCAATGGGCGTGGATAGCGGTTTGACTCACGGGGATT<br>TCCAAGTCTCCACCCCATTGACGTCAATGGGAGTTTGTTTTGGCACCAAAATCAACGGGACTTTCCAAAAT<br>GTCGTAACAACTCCGCCCCATTGACGCAAATGGGCGGTAGGCGTGTACGGTGGGAGGTCTATATAAGCAGA<br>GCTCGTTTAGTGAACCGTCAGATCTCTAGAgccgccaccatggtgagcaagggcgaggagctgttcaccgg<br>ggtggtgcccatcctggtcgagctggacggcgacgtaaacggccacaagttcagcgtgtccggcgagggcg<br>agggcgatgccacctacggcaagctgaccctgaagttcatctgcaccaccggcaagctgcccgtgccctgg<br>cccaccctcgtgaccaccctgacctacggcgtgcagtgcttcagccgctaccccgaccacatgaagcagca<br>cgacttcttcaagtccgccatgcccgaaggctacgtccaggagcgcaccatcttcttcaaggacgacggca<br>actacaagacccgcgccgaggtgaagttcgagggcgacaccctggtgaaccgcatcgagctgaagggcatc<br>gacttcaaggaggacggcaacatcctggggcacaagctggagtacaactacaacagccacaacgtctatat<br>catggccgacaagcagaagaacggcatcaaggtgaacttcaagatccgccacaacatcgaggacggcagcg<br>tgcagctcgccgaccactaccagcagaacacccccatcggcgacggccccgtgctgctgcccgacaaccac<br>tacctgagcacccagtccgccctgagcaaagaccccaacgagaagcgcgatcacatggtcctgctggagtt<br>cgtgaccgccgccgggatcactctcggcatggacgagctgtacaagtgacggcaataaaaagacagaataa

	aacgcacggtgttgggtcgtttgttcGTAGATGGCGCGCCTTTGTTGACCCGGTTGGAGGGCCTATTTCCC<br>ATGATTCCTTCATATTTGCATATACGATACAAGGCTGTTAGAGAGATAATTAGAATTAATTTGACTGTAAA<br>CACAAAGATATTAGTACAAAATACGTGACGTAGAAAGTAATAATTTCTTGGGTAGTTTGCAGTTTTAAAAT<br>TATGTTTTAAAATGGACTATCATATGCTTACCGTAACTTGAAAGTATTTCGATTTCTTGGCTTTATATATC<br>TTGTGGAAAGGACGAAACACCGATATAAACCTAATTACCTCGAGAGGGGACGGAAACCCGTCTTCGATGAA<br>GCGATTCAGAAGACTTGATATAAACCTAATTACCTCGAGAGGGGACTTTTTTACATGTGTCAGAGGTTTTC<br>ACCGTCATCACCGAAACGCGCGAGACGAAAGGGCCTCGTGATACGCCTATTTTTATAGGTTAATGTCATGA<br>TAATAATGGTTTCTTAGACGTCAGGTGGCACTTTTCGGGGAAATGTGCGCGGAACCCCTATTTGTTTATTT<br>TTCTAAATACATTCAAATATGTATCCGCTCATGAGACAATAACCCTGATAAATGCTTCAATAATATTGAAA<br>AAGGAAGAGTATGAGTATTCAACATTTCCGTGTCGCCCTTATTCCCTTTTTTGCGGCATTTTGCCTTCCTG<br>TTTTTGCTCACCCAGAAACGCTGGTGAAAGTAAAAGATGCTGAAGATCAGTTGGGTGCACGAGTGGGTTAC<br>ATCGAACTGGATCTCAACAGCGGTAAGATCCTTGAGAGTTTTCGCCCCGAAGAACGTTTTCCAATGATGAG<br>CACTTTTAAAGTTCTGCTATGTGGCGCGGTATTATCCCGTATTGACGCCGGGCAAGAGCAACTCGGTCGCC<br>GCATACACTATTCTCAGAATGACTTGGTTGAGTACTCACCAGTCACAGAAAAGCATCTTACGGATGGCATG<br>ACAGTAAGAGAATTATGCAGTGCTGCCATAACCATGAGTGATAACACTGCGGCCAACTTACTTCTGACAAC<br>GATCGGAGGACCGAAGGAGCTAACCGCTTTTTTGCACAACATGGGGGATCATGTAACTCGCCTTGATCGTT<br>GGGAACCGGAGCTGAATGAAGCCATACCAAACGACGAGCGTGACACCACGATGCCTGTAGCAATGGCAACA<br>ACGTTGCGCAAACTATTAACTGGCGAACTACTTACTCTAGCTTCCCGGCAACAATTAATAGACTGGATGGA<br>GGCGGATAAAGTTGCAGGACCACTTCTGCGCTCGGCCCTTCCGGCTGGCTGGTTTATTGCTGATAAATCTG<br>GAGCCGGTGAGCGTGGGTCTCGCGGTATCATTGCAGCACTGGGGCCAGATGGTAAGCCCTCCCGTATCGTA<br>GTTATCTACACGACGGGGAGTCAGGCAACTATGGATGAACGAAATAGACAGATCGCTGAGATAGGTGCCTC<br>ACTGATTAAGCATTGGTAACTGTCAGACCAAGTTTACTCATATATACTTTAGATTGATTTAAAACTTCATT<br>TTTAATTTAAAAGGATCTAGGTGAAGATCCTTTTTGATAATCTCATGACCAAAATCCCTTAACGTGAGTTT<br>TCGTTCCACTGAGCGTCAGACCCCGTAGAAAAGATCAAAGGATCTTCTTGAGATCCTTTTTTTCTGCGCGT<br>AATCTGCTGCTTGCAAACAAAAAAACCACCGCTACCAGCGGTGGTTTGTTTGCCGGATCAAGAGCTACCAA<br>CTCTTTTTCCGAAGGTAACTGGCTTCAGCAGAGCGCAGATACCAAATACTGTTCTTCTAGTGTAGCCGTAG<br>TTAGGCCACCACTTCAAGAACTCTGTAGCACCGCCTACATACCTCGCTCTGCTAATCCTGTTACCAGTGGC<br>TGCTGCCAGTGGCGATAAGTCGTGTCTTACCGGGTTGGACTCAAGACGATAGTTACCGGATAAGGCGCAGC<br>GGTCGGGCTGAACGGGGGGTTCGTGCACACAGCCCAGCTTGGAGCGAACGACCTACACCGAACTGAGATAC<br>CTACAGCGTGAGCTATGAGAAAGCGCCACGCTTCCCGAAGGGAGAAAGGCGGACAGGTATCCGGTAAGCGG<br>CAGGGTCGGAACAGGAGAGCGCACGAGGGAGCTTCCAGGGGGAAACGCCTGGTATCTTTATAGTCCTGTCG<br>GGTTTCGCCACCTCTGACTTGAGCGTCGATTTTTGTGATGCTCGTCAGGGGGGCGGAGCCTATGGAAAAAC<br>GCCAGCAACGCGGCCTTTTTCTTAAGC

**Plasmid**	**pDAC565**
**Description**	Expression of Csm-GFP complex (RNase mut) from separate promoters
**Utility**	RNA imaging
**Features**	Pcmv-FLAG-NLS-Csm1-pA; Pcmv-FLAG-NLS-Csm2-pA; Pcmv-FLAG-NLS-Csm3(RNase mut)-GFP-pA; Pcmv-FLAG-NLS-Csm4-pA; Pcmv-FLAG-NLS-Csm5-pA; Pcmv-FLAG-NLS-Cas6-pA; Pu6-crRNA-pT
**Sequence**	GTGATGCGGTTTTGGCAGTACATCAATGGGCGTGGATAGCGGTTTGACTCACGGGGATTTCCAAGTCTCCA<br>CCCCATTGACGTCAATGGGAGTTTGTTTTGGCACCAAAATCAACGGGACTTTCCAAAATGTCGTAACAACT<br>CCGCCCCATTGACGCAAATGGGCGGTAGGCGTGTACGGTGGGAGGTCTATATAAGCAGAGCTCGTTTAGTG<br>AACCGTCAGATCTCTAGAgccgccaccATGCACCATCACCATCACCATTCCGGCGATTACAAAGACGATGA<br>CGATAAGATGGCGCCTAAGAAGAAACGCAAAGTGCGGGGCATGAAGAAAGAAAAGATTGATCTGTTTTACG<br>GAGCCCTGCTGCACGACATCGGAAAGGTCATCCAGCGAGCAACCGGAGAGCGGAAGAAACACGCACTTGTG<br>GGCGCCGACTGGTTCGACGAGATCGCCGACAACCAAGTCATCTCGGATCAGATCCGGTACCATATGGCCAA<br>CTACCAGTCTGATAAGCTCGGCAACGATCACCTGGCTTACATCACCTACATTGCCGACAACATCGCCTCCG<br>GTGTCGACCGCCGGCAATCCAACGAAGAGTCAGACGAAGATACCTCCGCAAAGATCTGGGACACCTACACG<br>AACCAGGCCGACATCTTTAACGTGTTCGGAGCGCAGACCGATAAGCGGTACTTCAAGCCTACCGTGCTGAA<br>TCTCAAGTCGAAGCCCAACTTCGCGTCCGCCACTTACGAACCCTTTAGCAAGGGCGATTACGCTGCCATCG<br>CCACCCGGATTAAGAACGAACTGGCCGAGTTCGAGTTCAACCAAGTCCAGATTGACTCCCTGCTCAACCTT<br>TTCGAGGCTACTCTCTCCTTCGTGCCGTCAAGCACCAACACTAAGGAAATCGCCGACATCTCCCTGGCCGA<br>CCATTCCCGCTTGACTGCTGCCTTCGCTCTGGCGATCTACGACTACCTGGAGGACAAGGGTCGGCACAACT<br>ACAAAGAGGACCTGTTCACCAAAGTGTCAGCGTTCTATGAAGAAGAAGCCTTCCTGCTGGCCTCCTTCGAC<br>CTGTCGGGAATCCAGGACTTTATCTACAACATTAACATCGCAACTAACGGCGCGGCGAAGCAGCTGAAGGC<br>CCGGAGCCTCTACCTGGACTTTATGTCCGAGTACATCGCCGATAGCCTGCTGGACAAGCTGGGACTGAACA<br>GGGCTAACATGCTTTACGTCGGCGGCGGACACGCCTACTTCGTCCTGGCCAACACCGAAAAGACTGTGGAA<br>ACCCTGGTGCAGTTTGAGAAGGATTTCAACCAGTTCCTGTTGGCAAACTTCCAGACCCGCCTCTATGTGGC<br>CTTTGGCTGGGGTTCCTTCGCGGCCAAGGACATCATGTCCGAGCTGAATAGCCCCGAGTCCTACCGCCAAG<br>TGTACCAAAAGGCTTCGCGCATGATCTCCAAAAAGAAAATCTCCAGATACGACTACCAGACACTGATGCTC<br>CTGAATCGCGGTGGAAAGTCCTCAGAGAGAGAGTGCGAGATTTGCCACTCCGTGGAGAACCTGGTGTCCTA<br>CCACGACCAGAAAGTCTGTGACATTTGCCGGGGACTGTACCAGTTCTCGAAAGAAATTGCCCATGACCACT

	TCATCATTACCGAAAATGAGGGGCTGCCGATTGGACCAAACGCGTGCTTAAAGGGCGTGGCATTCGAAAAG<br>CTGTCCCAAGAAGCGTTCAGCCGGGTCTACGTGAAGAATGACTATAAGGCCGGTACCGTGAAGGCTACGCA<br>TGTGTTCGTGGGGGATTACCAGTGCGACGAGATCTACAACTACGCCGCCCTGAGCAAGAACGAGAACGGCC<br>TAGGCATCAAGAGACTGGCCGTGGTCCGGCTCGACGTGGATGACTTGGGCGCCGCCTTCATGGCCGGTTTC<br>AGCCAGCAGGGAAACGGACAATACTCCACTCTGTCAAGATCGGCCACATTCTCCCGGAGCATGTCGCTGTT<br>CTTCAAAGTGTACATTAACCAGTTCGCCTCCGACAAGAAGCTGAGCATTATCTACGCGGGCGGCGATGACG<br>TGTTCGCCATTGGATCGTGGCAGGATATCATCGCGTTCACTGTGGAACTTCGCGAAAACTTCATCAAGTGG<br>ACCAACGGGAAGCTCACCCTCTCCGCGGGGATAGGGTTGTTCGCCGACAAGACTCCTATTAGCCTGATGGC<br>TCACCAGACCGGGGAACTGGAAGAGGCCGCCAAGGGCAACGAAAAGGACTCCATCTCGCTGTTCTCAAGCG<br>ACTACACTTTCAAGTTTGATAGGTTCATCACTAACGTGTACGACGACAAACTGGAACAGATTAGATACTTC<br>TTCAACCATCAAGACGAGAGGGGAAAGAACTTCATCTATAAGCTTATTGAGCTTTTGAGGAACCACGACCG<br>CATGAATATGGCACGCCTCGCCTATTACCTCACTCGCCTGGAAGAACTGACCCGGGAGACTGACAGGGACA<br>AGTTCAAGACCTTCAAGAACCTGTTCTACTCCTGGTACACCAACAAGAACGATAAGGACCGGAAGGAAGCC<br>GAGCTCGCGCTCCTGCTGTACATCTACGAAATCAGAAAGGATTAAcggcaataaaaagacagaataaaacg<br>cacggtgttgggtcgtttgttcGCACACATTAGCTAGCCGTCAGCACACATTGTGATGCGGTTTTGGCAGT<br>ACATCAATGGGCGTGGATAGCGGTTTGACTCACGGGGATTTCCAAGTCTCCACCCCATTGACGTCAATGGG<br>AGTTTGTTTTGGCACCAAAATCAACGGGACTTTCCAAAATGTCGTAACAACTCCGCCCCATTGACGCAAAT<br>GGGCGGTAGGCGTGTACGGTGGGAGGTCTATATAAGCAGAGCTCGTTTAGTGAACCGTCAGATCTCTAGAg<br>ccgccaccATGGATTACAAAGACGATGACGATAAGATGGCGCCTAAGAAGAAACGCAAAGTGCGGGGCATG<br>ACCATCCTGACCGACGAGAACTACGTGGACATCGCCGAGAAAGCCATCCTGAAGCTGGAAAGAAACACCAG<br>AAATAGAAAGAACCCTGATGCCTTCTTCCTGACCACATCTAAGCTGCGGAACCTGCTGAGCCTGACAAGCA<br>CCCTGTTCGACGAGAGCAAGGTGAAGGAATACGACGCCCTGCTGGACAGAATCGCTTATCTGAGAGTGCAG<br>TTCGTGTACCAGGCCGGCAGAGAGATCGCCGTGAAAGATCTGATCGAGAAGGCCCAGATCCTGGAAGCTCT<br>GAAAGAGATCAAGGACCGGGAAACCCTGCAGAGATTCTGCAGATACATGGAAGCCCTGGTGGCCTACTTCA<br>AGTTCTACGGCGGCAAGGACTGAcggcaataaaaagacagaataaaacgcacggtgttgggtcgtttgttc<br>AGTTCTTTTGCCTTACTTTCAATGCATGCGGTGATGCGGTTTTGGCAGTACATCAATGGGCGTGGATAGCG<br>GTTTGACTCACGGGGATTTCCAAGTCTCCACCCCATTGACGTCAATGGGAGTTTGTTTTGGCACCAAAATC<br>AACGGGACTTTCCAAAATGTCGTAACAACTCCGCCCCATTGACGCAAATGGGCGGTAGGCGTGTACGGTGG<br>GAGGTCTATATAAGCAGAGCTCGTTTAGTGAACCGTCAGATCTCTAGAgccgccaccATGGATTACAAAGA<br>CGATGACGATAAGATGGCGCCTAAGAAGAAACGCAAAGTGCGGGGCATGACCTTCGCCAAGATCAAATTCA<br>GCGCCCAGATCCGGCTGGAAACCGGCCTGCACATCGGAGGATCTGATGCCTTTGCCGCTATCGGCGCCATC<br>GCCAGCCCTGTGATCAAGGACCCCATCACCAACCTGCCTATCATCCCCGGCTCTAGCCTGAAGGGCAAGAT<br>GAGAACACTGCTGGCCAAGGTGTACAACGAAAAGGTGGCCGAGAAGCCTAGCGACGACAGCGACATCCTGA<br>GCAGACTGTTCGGAAATAGCAAGGATAAGCGGTTCAAGATGGGCAGACTGATCTTCCGGGACGCCTTCCTG<br>AGCAACGCCGACGAGCTGGATTCTCTGGGCGTGCGGAGCTACACCGAGGTGAAGTTCGAGAACACCATCGA<br>TAGAATCACCGCCGAGGCCAATCCTAGACAGATCGAGAGAGCCATTCGGAACTCAACATTCGACTTCGAGC<br>TGATCTACGAGATCACTGATGAGAATGAGAACCAGGTCGAGGAAGATTTCAAGGTGATCAGAGACGGCCTG<br>AAGCTGCTGGAACTGGACTACCTGGGCGGAAGCGGCTCCAGAGGCTACGGCAAAGTGGCTTTTGAGAACCT<br>GAAAGCCACCACAGTGTTCGGCAACTACGACGTGAAAACCCTGAACGAGCTGCTGACCGCCGAAGTGGGCG<br>GAGGTGCTGCCGCAAtggtgagcaagggcgaggagctgttcaccggggtggtgcccatcctggtcgagctg<br>gacggcgacgtaaacggccacaagttcagcgtgtccggcgagggcgagggcgatgccacctacggcaagct<br>gaccctgaagttcatctgcaccaccggcaagctgcccgtgccctggcccaccctcgtgaccaccctgacct<br>acggcgtgcagtgcttcagccgctaccccgaccacatgaagcagcacgacttcttcaagtccgccatgccc<br>gaaggctacgtccaggagcgcaccatcttcttcaaggacgacggcaactacaagacccgcgccgaggtgaa<br>gttcgagggcgacaccctggtgaaccgcatcgagctgaagggcatcgacttcaaggaggacggcaacatcc<br>tggggcacaagctggagtacaactacaacagccacaacgtctatatcatggccgacaagcagaagaacggc<br>atcaaggtgaacttcaagatccgccacaacatcgaggacggcagcgtgcagctcgccgaccactaccagca<br>gaacacccccatcggcgacggccccgtgctgctgcccgacaaccactacctgagcacccagtccgccctga<br>gcaaagaccccaacgagaagcgcgatcacatggtcctgctggagttcgtgaccgccgccgggatcactctc<br>ggcatggacgagctgtacaagtgacggcaataaaaagacagaataaaacgcacggtgttgggtcgtttgtt<br>cTGGATTGCGAGAATGGACTAGTAGCAAACTGTGATGCGGTTTTGGCAGTACATCAATGGGCGTGGATAGC<br>GGTTTGACTCACGGGGATTTCCAAGTCTCCACCCCATTGACGTCAATGGGAGTTTGTTTTGGCACCAAAAT<br>CAACGGGACTTTCCAAAATGTCGTAACAACTCCGCCCCATTGACGCAAATGGGCGGTAGGCGTGTACGGTG<br>GGAGGTCTATATAAGCAGAGCTCGTTTAGTGAACCGTCAGATCTCTAGAgccgccaccATGGATTACAAAG<br>ACGATGACGATAAGATGGCGCCTAAGAAGAAACGCAAAGTGCGGGGCATGACTTACAAGCTCTACATTATG<br>ACCTTTCAAAACGCCCACTTCGGTTCCGGCACTCTGGACTCATCGAAGCTGACCTTCTCCGCGGATAGAAT<br>CTTCTCGGCACTCGTGCTCGAGGCTCTGAAGATGGGAAAGCTCGACGCCTTCTTGGCCGAGGCCAACCAGG<br>ATAAGTTCACTCTGACCGACGCGTTCCCATTCCAATTCGGTCCTTTCCTGCCGAAACCGATTGGTTACCCC<br>AAGCACGACCAGATCGACCAGTCTGTGGACGTGAAGGAAGTCCGCCGCCAAGCGAAGCTGTCCAAAAAGCT<br>CCAGTTCCTGGCTCTGGAAAACGTCGACGACTACCTGAACGGAGAGCTGTTTGAGAATGAGGAACACGCCG<br>TGATCGACACAGTGACCAAGAACCAGCCCCATAAAGATGATAATCTGTACCAAGTGGCCACCACTCGGTTC<br>TCGAACGACACCTCCCTTTACGTGATCGCCAACGAATCCGATCTGCTGAACGAACTGATGAGCAGCCTTCA<br>GTACTCCGGGCTGGGCGGCAAAAGGTCCTCAGGATTCGGCAGATTTGAGCTGGACATCCAGAACATTCCCT

	TGGAACTGTCCGACCGGCTGACGAAGAACCACAGCGACAAGGTCATGTCACTTACCACCGCCCTCCCGGTG<br>GACGCTGATCTCGAGGAAGCGATGGAAGATGGCCATTACCTGTTGACCAAGTCGTCCGGATTCGCATTCTC<br>CCACGCCACCAACGAAAACTATCGGAAGCAGGACCTGTACAAGTTCGCCTCCGGGAGCACCTTCAGCAAGA<br>CTTTCGAGGGACAGATCGTGGACGTGCGCCCTCTCGATTTCCCTCACGCCGTGCTGAACTACGCCAAGCCG<br>CTGTTCTTTAAGCTCGAAGTCTAAcggcaataaaaagacagaataaaacgcacggtgttgggtcgtttgtt<br>cGCACATTCAAAAACAGGCAATTGGACAAGCGTGATGCGGTTTTGGCAGTACATCAATGGGCGTGGATAGC<br>GGTTTGACTCACGGGGATTTCCAAGTCTCCACCCCATTGACGTCAATGGGAGTTTGTTTTGGCACCAAAAT<br>CAACGGGACTTTCCAAAATGTCGTAACAACTCCGCCCCATTGACGCAAATGGGCGGTAGGCGTGTACGGTG<br>GGAGGTCTATATAAGCAGAGCTCGTTTAGTGAACCGTCAGATCTCTAGAgccgccaccATGGATTACAAAG<br>ACGATGACGATAAGATGGCGCCTAAGAAGAAACGCAAAGTGCGGGGCATGAAAAATGACTACCGGACCTTC<br>AAGCTGAGCCTGCTGACCCTGGCTCCTATCCACATCGGCAACGGCGAGAAGTACACCAGCAGAGAATTCAT<br>CTACGAGAACAAGAAGTTCTACTTCCCCGACATGGGCAAGTTCTACAACAAGATGGTGGAAAAGAGACTGG<br>CCGAGAAGTTCGAGGCCTTCCTGATCCAGACCAGACCCAACGCCAGAAACAACCGGCTGATTTCTTTTCTG<br>AACGACAACAGAATCGCCGAAAGATCTTTTGGCGGCTACAGCATCAGTGAAACCGGCCTGGAATCTGATAA<br>GAACCCTAACAGCGCCGGAGCTATCAACGAGGTGAACAAATTCATCCGGGACGCCTTCGGAAATCCTTACA<br>TCCCAGGCAGCAGCCTGAAGGGCGCCATCCGCACCATCCTGATGAACACCACACCTAAGTGGAACAACGAG<br>AACGCCGTGAACGACTTCGGCAGATTCCCAAAGGAAAACAAGAACCTGATCCCTTGGGGACCTAAGAAAGG<br>CAAGGAATACGACGACCTGTTCAACGCCATCAGAGTGTCCGACAGCAAGCCCTTCGACAACAAAAGCCTGA<br>TCCTCGTGCAGAAGTGGGACTACAGCGCCAAAACCAACAAGGCCAAGCCTCTGCCTCTGTACAGAGAGTCT<br>ATCAGCCCTCTGACCAAGATCGAGTTCGAGATAACAACAACCACTGATGAGGCCGGCAGACTGATCGAGGA<br>ACTGGGAAAGCGGGCCCAGGCCTTTTATAAGGACTACAAGGCCTTTTTCCTGTCTGAATTCCCTGATGATA<br>AGATCCAGGCTAATCTGCAATACCCCATCTACCTGGGCGCCGGCAGCGGCGCTTGGACAAAGACCCTGTTT<br>AAGCAGGCCGACGGCATCCTGCAGCGGAGATACTCCAGAATGAAAACCAAGATGGTCAAGAAGGGCGTGCT<br>GAAGCTGACAAAGGCCCCTCTGAAAACAGTGAAGATCCCCAGCGGCAACCACAGCCTGGTGAAGAATCACG<br>AGAGCTTCTACGAGATGGGCAAAGCCAACTTCATGATCAAGGAAATCGACAAGTGAcggcaataaaaagac<br>agaataaaacgcacggtgttgggtcgtttgttcATGTGTGCCGGCCGGAGAATAAAAGTCTAAGTGATGCG<br>GTTTTGGCAGTACATCAATGGGCGTGGATAGCGGTTTGACTCACGGGGATTTCCAAGTCTCCACCCCATTG<br>ACGTCAATGGGAGTTTGTTTTGGCACCAAAATCAACGGGACTTTCCAAAATGTCGTAACAACTCCGCCCCA<br>TTGACGCAAATGGGCGGTAGGCGTGTACGGTGGGAGGTCTATATAAGCAGAGCTCGTTTAGTGAACCGTCA<br>GATCTCTAGAgccgccaccATGGATTACAAAGACGATGACGATAAGATGGCGCCTAAGAAGAAACGCAAAG<br>TGCGGGGCATGAAAAAGCTCGTGTTCACCTTTAAGCGGATCGACCACCCTGCTCAGGACCTGGCCGTGAAA<br>TTCCACGGCTTCCTGATGGAACAGCTGGATAGCGACTACGTGGACTACCTGCACCAGCAGCAGACCAACCC<br>CTACGCCACAAAGGTGATCCAGGGCAAAGAGAACACCCAGTGGGTCGTGCATCTGCTGACAGACGACATCG<br>AGGACAAGGTGTTCATGACCCTGCTGCAGATCAAGGAAGTGTCCCTGAACGACCTGCCTAAGTTGTCTGTG<br>GAAAAGGTGGAAATCCAGGAGCTGGGCGCTGATAAGCTGCTCGAGATCTTCAACAGCGAGGAAAACCAGAC<br>CTACTTCAGCATCATCTTCGAGACACCTACAGGCTTTAAAAGCCAGGGCAGCTACGTGATCTTCCCCAGCA<br>TGCGGCTGATCTTTCAGAGCCTGATGCAGAAGTACGGCAGACTGGTGGAAAACCAGCCTGAGATCGAGGAA<br>GATACCCTGGACTACCTGAGCGAGCACAGCACCATCACCAATTACAGACTGGAAACAAGCTACTTCAGAGT<br>GCATAGACAGAGAATCCCCGCCTTCCGGGGCAAGCTGACCTTCAAGGTGCAGGGAGCCCAGACACTGAAGG<br>CCTACGTGAAGATGCTGCTGACCTTCGGCGAGTACAGCGGCCTGGGCATGAAAACCAGCCTGGGAATGGGC<br>GGCATCAAGCTGGAAGAAAGAAAGGACTGAcggcaataaaaagacagaataaaacgcacggtgttgggtcg<br>tttgttcGTGCGATAGAGGGATCCAGTGCACCGGTTGGAGGGCCTATTTCCCATGATTCCTTCATATTTGC<br>ATATACGATACAAGGCTGTTAGAGAGATAATTAGAATTAATTTGACTGTAAACACAAAGATATTAGTACAA<br>AATACGTGACGTAGAAAGTAATAATTTCTTGGGTAGTTTGCAGTTTTAAAATTATGTTTTAAAATGGACTA<br>TCATATGCTTACCGTAACTTGAAAGTATTTCGATTTCTTGGCTTTATATATCTTGTGGAAAGGACGAAACA<br>CCGATATAAACCTAATTACCTCGAGAGGGGACGGAAACCCGTCTTCGATGAAGCGATTCAGAAGACTTGAT<br>ATAAACCTAATTACCTCGAGAGGGGACTTTTTTACATGTGTCAGAGGTTTTCACCGTCATCACCGAAACGC<br>GCGAGACGAAAGGGCCTCGTGATACGCCTATTTTTATAGGTTAATGTCATGATAATAATGGTTTCTTAGAC<br>GTCAGGTGGCACTTTTCGGGGAAATGTGCGCGGAACCCCTATTTGTTTATTTTTCTAAATACATTCAAATA<br>TGTATCCGCTCATGAGACAATAACCCTGATAAATGCTTCAATAATATTGAAAAAGGAAGAGTATGAGTATT<br>CAACATTTCCGTGTCGCCCTTATTCCCTTTTTTGCGGCATTTTGCCTTCCTGTTTTTGCTCACCCAGAAAC<br>GCTGGTGAAAGTAAAAGATGCTGAAGATCAGTTGGGTGCACGAGTGGGTTACATCGAACTGGATCTCAACA<br>GCGGTAAGATCCTTGAGAGTTTTCGCCCCGAAGAACGTTTTCCAATGATGAGCACTTTTAAAGTTCTGCTA<br>TGTGGCGCGGTATTATCCCGTATTGACGCCGGGCAAGAGCAACTCGGTCGCCGCATACACTATTCTCAGAA<br>TGACTTGGTTGAGTACTCACCAGTCACAGAAAAGCATCTTACGGATGGCATGACAGTAAGAGAATTATGCA<br>GTGCTGCCATAACCATGAGTGATAACACTGCGGCCAACTTACTTCTGACAACGATCGGAGGACCGAAGGAG<br>CTAACCGCTTTTTTGCACAACATGGGGGATCATGTAACTCGCCTTGATCGTTGGGAACCGGAGCTGAATGA<br>AGCCATACCAAACGACGAGCGTGACACCACGATGCCTGTAGCAATGGCAACAACGTTGCGCAAACTATTAA<br>CTGGCGAACTACTTACTCTAGCTTCCCGGCAACAATTAATAGACTGGATGGAGGCGGATAAAGTTGCAGGA<br>CCACTTCTGCGCTCGGCCCTTCCGGCTGGCTGGTTTATTGCTGATAAATCTGGAGCCGGTGAGCGTGGGTC<br>TCGCGGTATCATTGCAGCACTGGGGCCAGATGGTAAGCCCTCCCGTATCGTAGTTATCTACACGACGGGGA<br>GTCAGGCAACTATGGATGAACGAAATAGACAGATCGCTGAGATAGGTGCCTCACTGATTAAGCATTGGTAA<br>CTGTCAGACCAAGTTTACTCATATATACTTTAGATTGATTTAAAACTTCATTTTTAATTTAAAAGGATCTA

	GGTGAAGATCCTTTTTGATAATCTCATGACCAAAATCCCTTAACGTGAGTTTTCGTTCCACTGAGCGTCAG<br>ACCCCGTAGAAAAGATCAAAGGATCTTCTTGAGATCCTTTTTTTCTGCGCGTAATCTGCTGCTTGCAAACA<br>AAAAAACCACCGCTACCAGCGGTGGTTTGTTTGCCGGATCAAGAGCTACCAACTCTTTTTCCGAAGGTAAC<br>TGGCTTCAGCAGAGCGCAGATACCAAATACTGTTCTTCTAGTGTAGCCGTAGTTAGGCCACCACTTCAAGA<br>ACTCTGTAGCACCGCCTACATACCTCGCTCTGCTAATCCTGTTACCAGTGGCTGCTGCCAGTGGCGATAAG<br>TCGTGTCTTACCGGGTTGGACTCAAGACGATAGTTACCGGATAAGGCGCAGCGGTCGGGCTGAACGGGGGG<br>TTCGTGCACACAGCCCAGCTTGGAGCGAACGACCTACACCGAACTGAGATACCTACAGCGTGAGCTATGAG<br>AAAGCGCCACGCTTCCCGAAGGGAGAAAGGCGGACAGGTATCCGGTAAGCGGCAGGGTCGGAACAGGAGAG<br>CGCACGAGGGAGCTTCCAGGGGGAAACGCCTGGTATCTTTATAGTCCTGTCGGGTTTCGCCACCTCTGACT<br>TGAGCGTCGATTTTTGTGATGCTCGTCAGGGGGGCGGAGCCTATGGAAAAACGCCAGCAACGCGGCCTTTT<br>TCTTAAGC

**Plasmid**	**pDAC689**
**Description**	Expression of Cas13; RFP backbone
**Utility**	RNA KD
**Features**	Pcmv-NLS-Cas13-NLS-pA; Pu6-crRNA-pT; Pcmv-RFP-pA
**Sequence**	GTGATGCGGTTTTGGCAGTACATCAATGGGCGTGGATAGCGGTTTGACTCACGGGGATTTCCAAGTCTCCA<br>CCCCATTGACGTCAATGGGAGTTTGTTTTGGCACCAAAATCAACGGGACTTTCCAAAATGTCGTAACAACT<br>CCGCCCCATTGACGCAAATGGGCGGTAGGCGTGTACGGTGGGAGGTCTATATAAGCAGAGCTCGTTTAGTG<br>AACCGTCAGATCTCTAGAgccgccaccATGcccaagaagaagagaaaggtggaggccagcatcgagaagaa<br>gaagagcttcgccaagggcatgggagtgaagagcaccctggtgtccggctctaaggtgtacatgaccacat<br>ttgctgagggaagcgacgccaggctggagaagatcgtggagggcgatagcatcagatccgtgaacgaggga<br>gaggctttcagcgccgagatggctgacaagaacgctggctacaagatcggaaacgccaagttttcccaccc<br>aaagggctacgccgtggtggctaacaacccactgtacaccggaccagtgcagcaggacatgctgggactga<br>aggagacactggagaagaggtacttcggcgagtccgccgacggaaacgataacatctgcatccaggtcatc<br>cacaacatcctggatatcgagaagatcctggctgagtacatcacaaacgccgcttacgccgtgaacaacat<br>ctccggcctggacaaggatatcatcggcttcggaaagttttctaccgtgtacacatacgacgagttcaagg<br>atccagagcaccaccgggccgcttttaacaacaacgacaagctgatcaacgccatcaaggctcagtacgac<br>gagttcgataactttctggataaccccaggctgggctacttcggacaggctttcttttctaaggagggcag<br>aaactacatcatcaactacggaaacgagtgttacgatatcctggcgctgctgagcggactgaggcactggg<br>tggtgcacaacaacgaggaggagtctcggatctctcgcacctggctgtacaacctggacaagaacctggat<br>aacgagtacatctccacactgaactacctgtacgacaggatcaccaacgagctgacaaacagcttctccaa<br>gaactctgccgctaacgtgaactacatcgctgagaccctgggcatcaacccagctgagttcgctgagcagt<br>acttcagattttccatcatgaaggagcagaagaacctgggcttcaacatcacaaagctgagagaagtgatg<br>ctggacagaaaggatatgtccgagatcaggaagaaccacaaggtgttcgattctatcagaaccaaggtgta<br>cacaatgatggactttgtgatctacaggtactacatcgaggaggatgccaaggtggccgctgccaacaaga<br>gcctgcccgacaacgagaagtctctgagcgagaaggatatcttcgtgatcaacctgagaggctcctttaac<br>gacgatcagaaggacgctctgtactacgatgaggccaacaggatctggagaaagctggagaacatcatgca<br>caacatcaaggagttccggggaaacaagacccgcgagtacaagaagaaggacgctccaaggctgcctagga<br>tcctgcctgctggaagggacgtgagcgccttcagcaagctgatgtacgccctgacaatgtttctggacgga<br>aaggagatcaacgatctgctgaccacactgatcaacaagttcgacaacatccagtcttttctgaaagtgat<br>gcctctgatcggcgtgaacgctaagttcgtggaggagtacgccttctttaaggacagcgccaagatcgctg<br>atgagctgcggctgatcaagtcctttgccaggatgggagagccaatcgctgacgctaggagagctatgtac<br>atcgatgccatccggatcctgggaaccaacctgtcttacgacgagctgaaggctctggccgacaccttcag<br>cctggatgagaacggcaacaagctgaagaagggcaagcacggaatgcgcaacttcatcatcaacaacgtga<br>tcagcaacaagcggtttcactacctgatcagatacggcgacccagctcacctgcacgagatcgctaagaac<br>gaggccgtggtgaagttcgtgctgggacggatcgccgatatccagaagaagcagggccagaacggaaagaa<br>ccagatcgaccgctactacgagacctgcatcggcaaggataagggaaagtccgtgtctgagaaggtggacg<br>ctctgaccaagatcatcacaggcatgaactacgaccagttcgataagaagagatctgtgatcgaggacacc<br>ggaagggagaacgccgagagagagaagtttaagaagatcatcagcctgtacctgacagtgatctaccacat<br>cctgaagaacatcgtgaacatcaacgctagatacgtgatcggcttccactgcgtggagcgcgatgcccagc<br>tgtacaaggagaagggatacgacatcaacctgaagaagctggaggagaagggctttagctccgtgaccaag<br>ctgtgcgctggaatcgacgagacagccccccgacaagaggaaggatgtggagaaggagatggccgagagagc<br>taaggagagcatcgactccctggagtctgctaaccctaagctgtacgccaactacatcaagtactccgatg<br>agaagaaggccgaggagtttaccaggcagatcaacagagagaaggccaagaccgctctgaacgcctacctg<br>aggaacacaaagtggaacgtgatcatccgggaggacctgctgcgcatcgataacaagacctgtacactgtt<br>ccggaacaaggctgtgcacctggaggtggctcgctacgtgcacgcctacatcaacgacatcgccgaggtga<br>actcctactttcagctgtaccactacatcatgcagaggatcatcatgaacgagagatacgagaagtctagc<br>ggcaaggtgtctgagtacttcgacgccgtgaacgatgagaagaagtacaacgatagactgctgaagctgct<br>gtgcgtgcctttcggatactgtatcccacggtttaagaacctgagcatcgaggccctgttcgaccgcaacg<br>aggctgccaagtttgataaggagaagaagaaggtgagcggcaactccggatccggacctaagaaaaagagg<br>aaggtgtgacggcaataaaaagacagaataaaacgcacggtgttgggtcgtttgttcGTGCGATAGAGGGA<br>TCCCGCATTGAATTATGTGATGCGGTTTTGGCAGTACATCAATGGGCGTGGATAGCGGTTTGACTCACGGG

	GATTTCCAAGTCTCCACCCCATTGACGTCAATGGGAGTTTGTTTTGGCACCAAAATCAACGGGACTTTCCA<br>AAATGTCGTAACAACTCCGCCCCATTGACGCAAATGGGCGGTAGGCGTGTACGGTGGGAGGTCTATATAAG<br>CAGAGCTCGTTTAGTGAACCGTCAGATCTCTAGAgccgccaccATGGTGAGCAAGGGCGAGGAGGATAACA<br>TGGCCATCATCAAGGAGTTCATGCGCTTCAAGGTGCACATGGAGGGCTCCGTGAACGGCCACGAGTTCGAG<br>ATCGAGGGCGAGGGCGAGGGCCGCCCCTACGAGGGCACCCAGACCGCCAAGCTGAAGGTGACCAAGGGTGG<br>CCCCCTGCCCTTCGCCTGGGACATCCTGTCCCCTCAGTTCATGTACGGCTCCAAGGCCTACGTGAAGCACC<br>CCGCCGACATCCCCGACTACTTGAAGCTGTCCTTCCCCGAGGGCTTCAAGTGGGAGCGCGTGATGAACTTC<br>GAGGACGGCGGCGTGGTGACCGTGACCCAGGACTCCTCCCTGCAGGACGGCGAGTTCATCTACAAGGTGAA<br>GCTGCGCGGCACCAACTTCCCCTCCGACGGCCCCGTAATGCAGAAGAAAACCATGGGCTGGGAGGCCTCCT<br>CCGAGCGGATGTACCCCGAGGACGGCGCCCTGAAGGGCGAGATCAAGCAGAGGCTGAAGCTGAAGGACGGC<br>GGCCACTACGACGCTGAGGTCAAGACCACCTACAAGGCCAAGAAGCCCGTGCAGCTGCCCGGCGCCTACAA<br>CGTCAACATCAAGTTGGACATCACCTCCCACAACGAGGACTACACCATCGTGGAACAGTACGAACGCGCCG<br>AGGGCCGCCACTCCACCGGCGGCATGGACGAGCTGTACAAGTAAcggcaataaaaagacagaataaaacgc<br>acggtgttgggtcgtttgttcGAGCAGATTGTACTGAGAGTGCACCGGTTGGAGGGCCTATTTCCCATGAT<br>TCCTTCATATTTGCATATACGATACAAGGCTGTTAGAGAGATAATTAGAATTAATTTGACTGTAAACACAA<br>AGATATTAGTACAAAATACGTGACGTAGAAAGTAATAATTTCTTGGGTAGTTTGCAGTTTTAAAATTATGT<br>TTTAAAATGGACTATCATATGCTTACCGTAACTTGAAAGTATTTCGATTTCTTGGCTTTATATATCTTGTG<br>GAAAGGACGAAACACCGcaagtaaacccctaccaactggtcggggtttgaaacgggtcttcgagaagaccc<br>TTTTTTACATGTGTCAGAGGTTTTCACCGTCATCACCGAAACGCGCGAGACGAAAGGGCCTCGTGATACGC<br>CTATTTTTATAGGTTAATGTCATGATAATAATGGTTTCTTAGACGTCAGGTGGCACTTTTCGGGGAAATGT<br>GCGCGGAACCCCTATTTGTTTATTTTTCTAAATACATTCAAATATGTATCCGCTCATGAGACAATAACCCT<br>GATAAATGCTTCAATAATATTGAAAAAGGAAGAGTATGAGTATTCAACATTTCCGTGTCGCCCTTATTCCC<br>TTTTTTGCGGCATTTTGCCTTCCTGTTTTTGCTCACCCAGAAACGCTGGTGAAAGTAAAAGATGCTGAAGA<br>TCAGTTGGGTGCACGAGTGGGTTACATCGAACTGGATCTCAACAGCGGTAAGATCCTTGAGAGTTTTCGCC<br>CCGAAGAACGTTTTCCAATGATGAGCACTTTTAAAGTTCTGCTATGTGGCGCGGTATTATCCCGTATTGAC<br>GCCGGGCAAGAGCAACTCGGTCGCCGCATACACTATTCTCAGAATGACTTGGTTGAGTACTCACCAGTCAC<br>AGAAAAGCATCTTACGGATGGCATGACAGTAAGAGAATTATGCAGTGCTGCCATAACCATGAGTGATAACA<br>CTGCGGCCAACTTACTTCTGACAACGATCGGAGGACCGAAGGAGCTAACCGCTTTTTTGCACAACATGGGG<br>GATCATGTAACTCGCCTTGATCGTTGGGAACCGGAGCTGAATGAAGCCATACCAAACGACGAGCGTGACAC<br>CACGATGCCTGTAGCAATGGCAACAACGTTGCGCAAACTATTAACTGGCGAACTACTTACTCTAGCTTCCC<br>GGCAACAATTAATAGACTGGATGGAGGCGGATAAAGTTGCAGGACCACTTCTGCGCTCGGCCCTTCCGGCT<br>GGCTGGTTTATTGCTGATAAATCTGGAGCCGGTGAGCGTGGGTCTCGCGGTATCATTGCAGCACTGGGGCC<br>AGATGGTAAGCCCTCCCGTATCGTAGTTATCTACACGACGGGGAGTCAGGCAACTATGGATGAACGAAATA<br>GACAGATCGCTGAGATAGGTGCCTCACTGATTAAGCATTGGTAACTGTCAGACCAAGTTTACTCATATATA<br>CTTTAGATTGATTTAAAACTTCATTTTTAATTTAAAAGGATCTAGGTGAAGATCCTTTTTGATAATCTCAT<br>GACCAAAATCCCTTAACGTGAGTTTTCGTTCCACTGAGCGTCAGACCCCGTAGAAAAGATCAAAGGATCTT<br>CTTGAGATCCTTTTTTTCTGCGCGTAATCTGCTGCTTGCAAACAAAAAAACCACCGCTACCAGCGGTGGTT<br>TGTTTGCCGGATCAAGAGCTACCAACTCTTTTTCCGAAGGTAACTGGCTTCAGCAGAGCGCAGATACCAAA<br>TACTGTTCTTCTAGTGTAGCCGTAGTTAGGCCACCACTTCAAGAACTCTGTAGCACCGCCTACATACCTCG<br>CTCTGCTAATCCTGTTACCAGTGGCTGCTGCCAGTGGCGATAAGTCGTGTCTTACCGGGTTGGACTCAAGA<br>CGATAGTTACCGGATAAGGCGCAGCGGTCGGGCTGAACGGGGGGTTCGTGCACACAGCCCAGCTTGGAGCG<br>AACGACCTACACCGAACTGAGATACCTACAGCGTGAGCTATGAGAAAGCGCCACGCTTCCCGAAGGGAGAA<br>AGGCGGACAGGTATCCGGTAAGCGGCAGGGTCGGAACAGGAGAGCGCACGAGGGAGCTTCCAGGGGGAAAC<br>GCCTGGTATCTTTATAGTCCTGTCGGGTTTCGCCACCTCTGACTTGAGCGTCGATTTTTGTGATGCTCGTC<br>AGGGGGGCGGAGCCTATGGAAAAACGCCAGCAACGCGGCCTTTTTCTTAAGC

**Plasmid**	**pDAC690**
**Description**	Expression of shRNA; RFP backbone
**Utility**	RNA KD
**Features**	Pu6-shRNA-pT; Pcmv-RFP-pA
**Sequence**	GTGATGCGGTTTTGGCAGTACATCAATGGGCGTGGATAGCGGTTTGACTCACGGGGATTTCCAAGTCTCCA<br>CCCCATTGACGTCAATGGGAGTTTGTTTTGGCACCAAAATCAACGGGACTTTCCAAAATGTCGTAACAACT<br>CCGCCCCATTGACGCAAATGGGCGGTAGGCGTGTACGGTGGGAGGTCTATATAAGCAGAGCTCGTTTAGTG<br>AACCGTCAGATCTCTAGAgccgccaccATGGTGAGCAAGGGCGAGGAGGATAACATGGCCATCATCAAGGA<br>GTTCATGCGCTTCAAGGTGCACATGGAGGGCTCCGTGAACGGCCACGAGTTCGAGATCGAGGGCGAGGGCG<br>AGGGCCGCCCCTACGAGGGCACCCAGACCGCCAAGCTGAAGGTGACCAAGGGTGGCCCCCTGCCCTTCGCC<br>TGGGACATCCTGTCCCCTCAGTTCATGTACGGCTCCAAGGCCTACGTGAAGCACCCCGCCGACATCCCCGA<br>CTACTTGAAGCTGTCCTTCCCCGAGGGCTTCAAGTGGGAGCGCGTGATGAACTTCGAGGACGGCGGCGTGG<br>TGACCGTGACCCAGGACTCCTCCCTGCAGGACGGCGAGTTCATCTACAAGGTGAAGCTGCGCGGCACCAAC<br>TTCCCCTCCGACGGCCCCGTAATGCAGAAGAAAACCATGGGCTGGGAGGCCTCCTCCGAGCGGATGTACCC<br>CGAGGACGGCGCCCTGAAGGGCGAGATCAAGCAGAGGCTGAAGCTGAAGGACGGCGGCCACTACGACGCTG<br>AGGTCAAGACCACCTACAAGGCCAAGAAGCCCGTGCAGCTGCCCGGCGCCTACAACGTCAACATCAAGTTG<br>GACATCACCTCCCACAACGAGGACTACACCATCGTGGAACAGTACGAACGCGCCGAGGGCCGCCACTCCAC

	CGGCGGCATGGACGAGCTGTACAAGTAAcggcaataaaaagacagaataaaacgcacggtgttgggtcgtt<br>tgttcGAGCAGATTGTACTGAGAGTGCACCGGTTGGAGGGCCTATTTCCCATGATTCCTTCATATTTGCAT<br>ATACGATACAAGGCTGTTAGAGAGATAATTAGAATTAATTTGACTGTAAACACAAAGATATTAGTACAAAA<br>TACGTGACGTAGAAAGTAATAATTTCTTGGGTAGTTTGCAGTTTTAAAATTATGTTTTAAAATGGACTATC<br>ATATGCTTACCGTAACTTGAAAGTATTTCGATTTCTTGGCTTTATATATCTTGTGGAAAGGACGAAACACC<br>GcgtcttcCCTGACCCAgaagacgCTTTTTTACATGTGTCAGAGGTTTTCACCGTCATCACCGAAACGCGC<br>GAGACGAAAGGGCCTCGTGATACGCCTATTTTTATAGGTTAATGTCATGATAATAATGGTTTCTTAGACGT<br>CAGGTGGCACTTTTCGGGGAAATGTGCGCGGAACCCCTATTTGTTTATTTTTCTAAATACATTCAAATATG<br>TATCCGCTCATGAGACAATAACCCTGATAAATGCTTCAATAATATTGAAAAAGGAAGAGTATGAGTATTCA<br>ACATTTCCGTGTCGCCCTTATTCCCTTTTTTGCGGCATTTTGCCTTCCTGTTTTTGCTCACCCAGAAACGC<br>TGGTGAAAGTAAAAGATGCTGAAGATCAGTTGGGTGCACGAGTGGGTTACATCGAACTGGATCTCAACAGC<br>GGTAAGATCCTTGAGAGTTTTCGCCCCGAAGAACGTTTTCCAATGATGAGCACTTTTAAAGTTCTGCTATG<br>TGGCGCGGTATTATCCCGTATTGACGCCGGGCAAGAGCAACTCGGTCGCCGCATACACTATTCTCAGAATG<br>ACTTGGTTGAGTACTCACCAGTCACAGAAAAGCATCTTACGGATGGCATGACAGTAAGAGAATTATGCAGT<br>GCTGCCATAACCATGAGTGATAACACTGCGGCCAACTTACTTCTGACAACGATCGGAGGACCGAAGGAGCT<br>AACCGCTTTTTTGCACAACATGGGGGATCATGTAACTCGCCTTGATCGTTGGGAACCGGAGCTGAATGAAG<br>CCATACCAAACGACGAGCGTGACACCACGATGCCTGTAGCAATGGCAACAACGTTGCGCAAACTATTAACT<br>GGCGAACTACTTACTCTAGCTTCCCGGCAACAATTAATAGACTGGATGGAGGCGGATAAAGTTGCAGGACC<br>ACTTCTGCGCTCGGCCCTTCCGGCTGGCTGGTTTATTGCTGATAAATCTGGAGCCGGTGAGCGTGGGTCTC<br>GCGGTATCATTGCAGCACTGGGGCCAGATGGTAAGCCCTCCCGTATCGTAGTTATCTACACGACGGGGAGT<br>CAGGCAACTATGGATGAACGAAATAGACAGATCGCTGAGATAGGTGCCTCACTGATTAAGCATTGGTAACT<br>GTCAGACCAAGTTTACTCATATATACTTTAGATTGATTTAAAACTTCATTTTTAATTTAAAAGGATCTAGG<br>TGAAGATCCTTTTTGATAATCTCATGACCAAAATCCCTTAACGTGAGTTTTCGTTCCACTGAGCGTCAGAC<br>CCCGTAGAAAAGATCAAAGGATCTTCTTGAGATCCTTTTTTTCTGCGCGTAATCTGCTGCTTGCAAACAAA<br>AAAACCACCGCTACCAGCGGTGGTTTGTTTGCCGGATCAAGAGCTACCAACTCTTTTTCCGAAGGTAACTG<br>GCTTCAGCAGAGCGCAGATACCAAATACTGTTCTTCTAGTGTAGCCGTAGTTAGGCCACCACTTCAAGAAC<br>TCTGTAGCACCGCCTACATACCTCGCTCTGCTAATCCTGTTACCAGTGGCTGCTGCCAGTGGCGATAAGTC<br>GTGTCTTACCGGGTTGGACTCAAGACGATAGTTACCGGATAAGGCGCAGCGGTCGGGCTGAACGGGGGGTT<br>CGTGCACACAGCCCAGCTTGGAGCGAACGACCTACACCGAACTGAGATACCTACAGCGTGAGCTATGAGAA<br>AGCGCCACGCTTCCCGAAGGGAGAAAGGCGGACAGGTATCCGGTAAGCGGCAGGGTCGGAACAGGAGAGCG<br>CACGAGGGAGCTTCCAGGGGGAAACGCCTGGTATCTTTATAGTCCTGTCGGGTTTCGCCACCTCTGACTTG<br>AGCGTCGATTTTTGTGATGCTCGTCAGGGGGGCGGAGCCTATGGAAAAACGCCAGCAACGCGGCCTTTTTC<br>TTAAGC
