# Supplementary material for: Precise transcript targeting by CRISPR-Csm complexes
Source: Nat Biotechnol. 2023 Jan 23;41(9):1256–64. doi: 10.1038/s41587-022-01649-9 (PMC10497410; doi:10.1038/s41587-022-01649-9)
Supplement: Supplementary file 2 — Reporting Summary [file 41587_2022_1649_MOESM2_ESM.pdf]

nature research

Corresponding author(s): Jennifer A. Doudna

Last updated by author(s): 2022/12/10

Reporting Summary

Nature Research wishes to improve the reproducibility of the work that we publish. This form provides structure for consistency and transparency in reporting. For further information on Nature Research policies, see our Editorial Policies and the Editorial Policy Checklist.

Statistics

For all statistical analyses, confirm that the following items are present in the figure legend, table legend, main text, or Methods section.

n/a	Confirmed	
☐	☒	The exact sample size (*n*) for each experimental group/condition, given as a discrete number and unit of measurement
☒	☐	A statement on whether measurements were taken from distinct samples or whether the same sample was measured repeatedly
☒	☐	The statistical test(s) used AND whether they are one- or two-sided<br>*Only common tests should be described solely by name; describe more complex techniques in the Methods section.*
☒	☐	A description of all covariates tested
☒	☐	A description of any assumptions or corrections, such as tests of normality and adjustment for multiple comparisons
☐	☒	A full description of the statistical parameters including central tendency (e.g. means) or other basic estimates (e.g. regression coefficient) AND variation (e.g. standard deviation) or associated estimates of uncertainty (e.g. confidence intervals)
☒	☐	For null hypothesis testing, the test statistic (e.g. *F*, *t*, *r*) with confidence intervals, effect sizes, degrees of freedom and *P* value noted<br>*Give P values as exact values whenever suitable.*
☒	☐	For Bayesian analysis, information on the choice of priors and Markov chain Monte Carlo settings
☒	☐	For hierarchical and complex designs, identification of the appropriate level for tests and full reporting of outcomes
☒	☐	Estimates of effect sizes (e.g. Cohen's *d*, Pearson's *r*), indicating how they were calculated

Our web collection on statistics for biologists contains articles on many of the points above.

Software and code

Policy information about availability of computer code

Data collection	ZEN (2012), Attune Cytometric Software (v5.1.1), Sony Cell Sorter Software (v2.1.5), Image Studio (v5.2)
Data analysis	FlowJo (v10.7.1), FIJI (ImageJ), GraphPad Prism (v9.3.1), BWA-MEM (v0.7.17), Picard Tools (v2.21.9), CutAdapt (v4.1), STAR (v2.7.10a), featureCounts (v2.0.3), EdgeR (v3.36.0), Samtools (v4.1.1), R (v4.1.1), Python (v3.9.12), Pysamstats (v1.1.2), FastQC (v0.11.9), matplotlib (v3.6.0), custom code available upon request

For manuscripts utilizing custom algorithms or software that are central to the research but not yet described in published literature, software must be made available to editors and reviewers. We strongly encourage code deposition in a community repository (e.g. GitHub). See the Nature Research guidelines for submitting code & software for further information.

Data

Policy information about availability of data

All manuscripts must include a data availability statement. This statement should provide the following information, where applicable:
- Accession codes, unique identifiers, or web links for publicly available datasets
- A list of figures that have associated raw data
- A description of any restrictions on data availability

RNA-seq and DNA-seq datasets have been deposited at GEO (accession number GSE###). Essential plasmids have been deposited at Addgene (plasmid IDs 195237-195242). Unprocessed blots have been included with this article as Source Data. Microscope image files have been deposited at Zenodo (doi ###). The GRCh38 reference genome is publicly available (GENCODE Release 39).

Field-specific reporting

Please select the one below that is the best fit for your research. If you are not sure, read the appropriate sections before making your selection.

☒ Life sciences ☐ Behavioural & social sciences ☐ Ecological, evolutionary & environmental sciences

For a reference copy of the document with all sections, see nature.com/documents/nr-reporting-summary-flat.pdf

Life sciences study design

All studies must disclose on these points even when the disclosure is negative.

Sample size	No sample size calculation was performed for our study. All experiments were performed with three biological replicates, except for RNA-seq and DNA-seq experiments, which were performed once due to time and cost constraints. 100 cells were counted for each microscopy experiment. Sample sizes were chosen based on the standard in the field and prior knowledge of experimental variation.
Data exclusions	No data were excluded.
Replication	All experiments were performed with three biological replicates, except for RNA-seq and DNA-seq experiments, which were performed once due to time and cost constraints. All reported data was reproducible. Means and standard deviations are provided in each figure.
Randomization	Randomization was unnecessary for our study because it is not a subjective trial and the results presented here were purely based on objective and quantitative measurements under controlled conditions. No animal or human subjects were used in this study. Randomization is not typically used for these types of experiments.
Blinding	Blinding was unnecessary for our study because it is not a subjective trial and the results presented here were purely based on objective and quantitative measurements under controlled conditions. No animal or human subjects were used in this study. Blinding is not typically used for these types of experiments.

Reporting for specific materials, systems and methods

We require information from authors about some types of materials, experimental systems and methods used in many studies. Here, indicate whether each material, system or method listed is relevant to your study. If you are not sure if a list item applies to your research, read the appropriate section before selecting a response.

Materials & experimental systems

n/a	Involved in the study	
☐	☒	Antibodies
☐	☒	Eukaryotic cell lines
☒	☐	Palaeontology and archaeology
☒	☐	Animals and other organisms
☒	☐	Human research participants
☒	☐	Clinical data
☒	☐	Dual use research of concern

Methods

n/a	Involved in the study	
☒	☐	ChIP-seq
☐	☒	Flow cytometry
☒	☐	MRI-based neuroimaging

Antibodies

Antibodies used	Primary antibodies: mouse anti-FLAG (Sigma, F1804, Lot# SLCC6485), rabbit anti-GAPDH (Cell Signaling Technology, 14C10, Lot# 14). Secondary antibodies: IRDye 680RD goat anti-mouse (LI-COR, 926-68070, Lot# C90910-21), IRDye 800CW goat anti-rabbit (LI-COR, 926-32211, Lot# C90723-19), Alexa Fluor 555 goat anti-mouse (Invitrogen, A21424, Lot# 2123594).
Validation	The anti-FLAG and anti-GAPDH primary antibodies have been extensively used in the field and validated by the manufacturer. Please see citations and data on each of the product pages demonstrating antibody validity for the purposes of Western blot and immunofluorescence.

Eukaryotic cell lines

Policy information about cell lines

Cell line source(s)	HEK293T, HEK293T-GFP, and HEK293T-GFP/RFP cells were obtained from the UC Berkeley Cell Culture Facility.
Authentication	All cell lines were authenticated by and purchased commercially from the UC Berkeley Cell Culture Facility.
Mycoplasma contamination	All cell lines were confirmed to be mycoplasma-free (abm, PCR mycoplasma detection kit).

Commonly misidentified lines (See ICLAC register)	No commonly misidentified cell lines were used in this study.

Flow Cytometry

Plots

Confirm that:

☒ The axis labels state the marker and fluorochrome used (e.g. CD4-FITC).

☒ The axis scales are clearly visible. Include numbers along axes only for bottom left plot of group (a 'group' is an analysis of identical markers).

☒ All plots are contour plots with outliers or pseudocolor plots.

☒ A numerical value for number of cells or percentage (with statistics) is provided.

Methodology

Sample preparation	HEK293T cells were trypsinized, quenched in medium, pelleted, resuspended in PBS, and filtered through a 40-micron cell strainer prior to flow cytometry or FACS.
Instrument	Attune NxT acoustic focusing cytometer (Thermo Fisher Scientific); Sony Cell Sorter SH800Z
Software	Attune Cytometric Software v5.1.1; Sony Cell Sorter Software v2.1.5; FlowJo v10.7.1.
Cell population abundance	mCherry-positive (transfected) cells were sorted with an efficiency of >95% prior to downstream analyses.
Gating strategy	1) FSC-A/SSC-A was used to remove debris; 2) FSC-A/FSC-H was used to define single cells; 3) FSC-A/GFP-A or RFP-A was used to determine fluorescence. Gating for RFP-positive (transfected) cells was based on RFP-negative (untransfected) cells. GFP/RFP knockdown was based on cells treated with non-targeting crRNA controls. We have included example gating strategies in Supplementary Fig. 1.

☒ Tick this box to confirm that a figure exemplifying the gating strategy is provided in the Supplementary Information.
