# Supplementary figures and images for: Precise transcript targeting by CRISPR-Csm complexes
Source: Nat Biotechnol. 2023 Jan 23;41(9):1256–64. doi: 10.1038/s41587-022-01649-9 (PMC10497410; doi:10.1038/s41587-022-01649-9)

Unprocessed Western blots

Fig. 1d

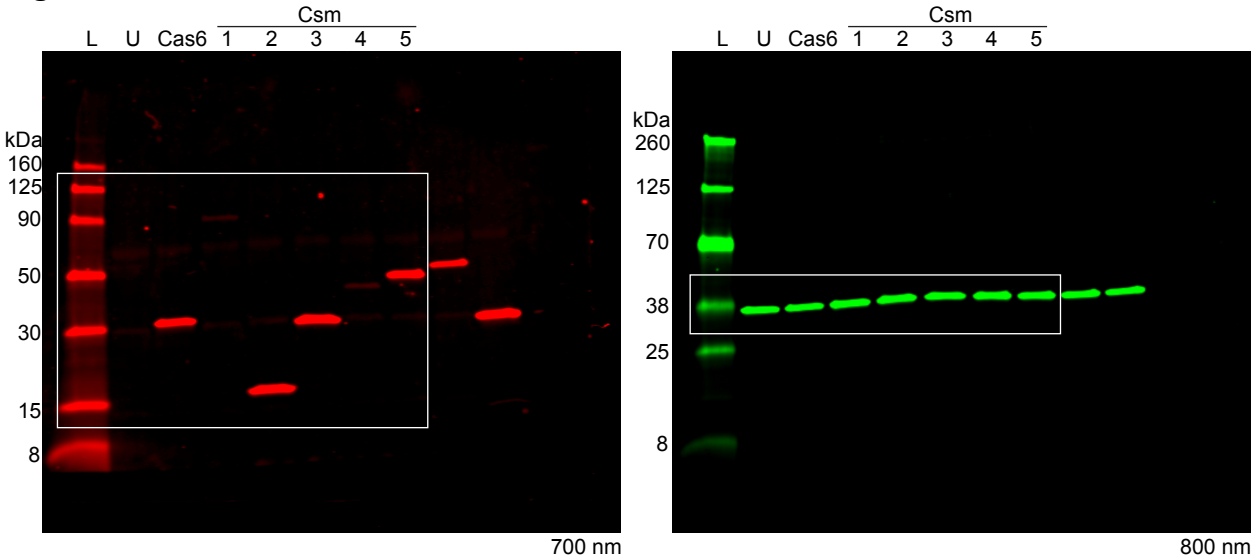

Fig. 1k

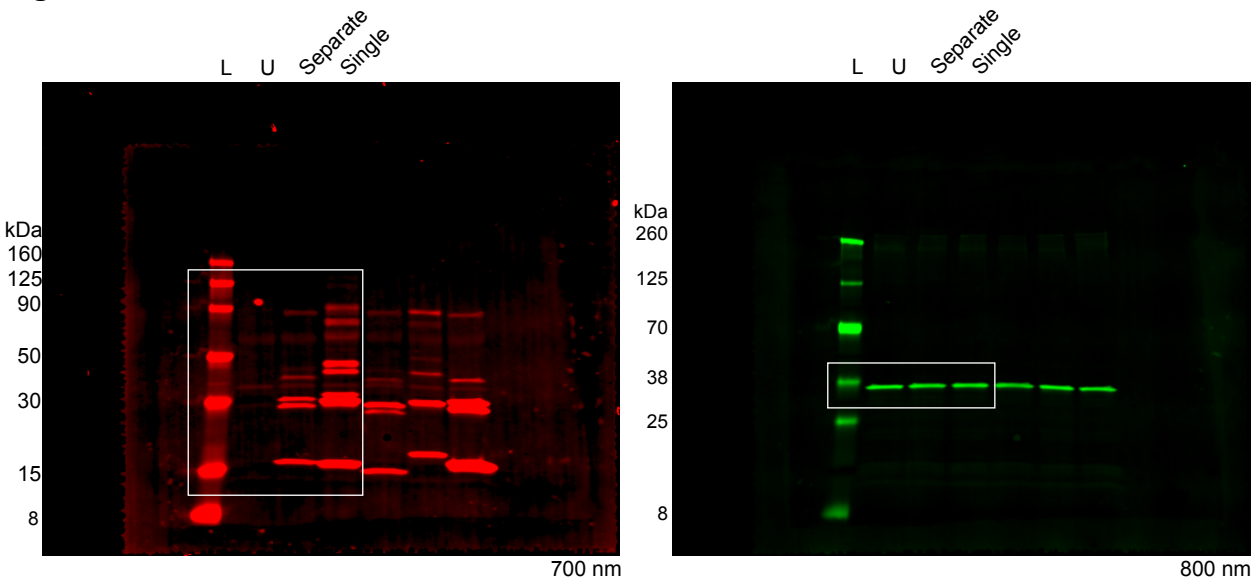

Supplement: Source Data Fig. 1 — Unprocessed western blots. [file 41587_2022_1649_MOESM3_ESM.pdf]
